# The role of *SAMM50* in non‐alcoholic fatty liver disease: from genetics to mechanisms

**DOI:** 10.1002/2211-5463.13146

**Published:** 2021-05-27

**Authors:** Zuyin Li, Weixing Shen, Gang Wu, Changjiang Qin, Yijie Zhang, Yupeng Wang, Guohe Song, Chao Xiao, Xin Zhang, Guilong Deng, Ruitao Wang, Xiaoliang Wang

**Affiliations:** ^1^ Department of General Surgery Shanghai General Hospital Shanghai Jiao Tong University School of Medicine China; ^2^ Department of General Surgery Qingpu Branch of Zhongshan Hospital Affiliated to Fudan University Shanghai China; ^3^ Department of General Surgery Henan Provincial People's Hospital People's Hospital of Zhengzhou University Henan China; ^4^ Department of General Surgery Huaihe Hospital of Henan University Kaifeng China; ^5^ Department of Medical Oncology Huaihe Hospital of Henan University Kaifeng China; ^6^ Department of Liver Surgery and Transplantation Liver Cancer Institute Zhongshan Hospital Fudan University Shanghai China; ^7^ Key Laboratory of Carcinogenesis and Cancer Invasion (Ministry of Education) Fudan University Shanghai China; ^8^ Department of General Surgery Huashan Hospital Fudan University Shanghai China

**Keywords:** fatty acid oxidation, NAFLD, *SAMM50*, SNPs

## Abstract

Non‐alcoholic fatty liver disease (NAFLD) is characterized by hepatic lipid accumulation. *SAMM50* encodes Sam50, a mitochondrial outer membrane protein involved in the removal of reactive oxygen species, mitochondrial morphology and regulation of mitophagy. Certain single nucleotide polymorphisms of *SAMM50* have been reported to be correlated with NAFLD. However, the contribution of *SAMM50* polymorphisms to the occurrence and severity of fatty liver in the Chinese Han cohort has rarely been reported. Here, we investigated the association between *SAMM50* polymorphisms (rs738491 and rs2073082) and NAFLD in a Chinese Han cohort, as well as the mechanistic basis of this association. Clinical information and blood samples were collected from 380 NAFLD cases and 380 normal subjects for the detection of genotypes and biochemical parameters. Carriers of the rs738491 T allele or rs2073082 G allele of *SAMM50* exhibit increased susceptibility to NAFLD [odds ratio (OR) = 1.39; 95% confidence interval (CI) = 1.14–1.71, *P* = 0.001; OR = 1.31; 95% CI = 1.05–1.62, *P* = 0.016, respectively] and are correlated with elevated serum triglyceride, alanine aminotransferase and aspartate aminotransferase levels. The presence of the T allele (TT + CT) of rs738491 (*P* < 0.01) or G allele (AG + GG) of rs2073082 (*P* = 0.03) is correlated with the severity of fatty liver in the NAFLD cohort. *In vitro* studies indicated that *SAMM50* gene polymorphisms decrease its expression and *SAMM50* deficiency results in increased lipid accumulation as a result of a decrease in fatty acid oxidation. Overexpression of *SAMM50* enhances fatty acid oxidation and mitigates intracellular lipid accumulation. Our results confirm the association between the *SAMM50* rs738491 and rs2073082 polymorphisms and the risk of fatty liver in a Chinese cohort. The underlying mechanism may be related to decreased fatty acid oxidation caused by *SAMM50* deficiency.

## Graphical abstract

The present study demonstrated that two single nucleotide polymorphismss (rs738491 and rs2073082) of the *SAMM50* gene are associated with susceptibility to and severity of fatty liver in a Chinese Han population. Genetic variants of *SAMM50* decreased its expression. *In vitro* studies indicated that *SAMM50* deficiency impaired fatty acid β‐oxidation and caused intracellular lipid accumulation.

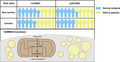

**Abbreviations**
β‐HB: β‐hydroxybutyrate
AcAc: acetoacetate
AKP: alkaline phosphatase
ALT: alanine aminotransferase
ANOVA: analysis of variance
AST: aspartate aminotransferase
BMI: body mass index
CI: confidence interval
DBIL: direct bilrubin
DMEM: Dulbecco’s modified Eagle’s medium
FAO: fatty acid oxidation
GA: glycated albumin
GLU: blood glucose
GWAS: genome‐wide association studies
HDL: high‐density lipoprotein
IHC: immunohistochemistry
LDL: low‐density lipoprotein
LSD: least significant difference
NAFLD: non‐alcoholic fatty liver disease
OA: oleic acid
OCR: oxygen consumption rate
OR: odds ratio
PA: palmitic acid
qRT‐PCR: quantitative real‐time PCR
SNP: single nucleotide polymorphism
TC: total cholesterol
TG: triglyceride

Non‐alcoholic fatty liver disease (NAFLD) is characterized by hepatic lipid accumulation and is strongly associated with obesity, diabetes and other features of the metabolic syndrome [1]. NAFLD has a wide spectrum, ranging from hepatic steatosis to histological evidence of necroinflammation and non‐alcoholic steatohepatitis, which may evolve into cirrhosis, final‐stage liver disease or hepatocellular carcinoma [2]. NAFLD has now been considered a serious worldwide health problem. Genome‐wide association studies (GWAS) confirm a bulk of genetic variants correlated with vulnerability to NAFLD, and post‐GWAS analyses have determined several loci with strong genetic associations with one or more phases of NAFLD. Several studies have revealed significant single nucleotide polymorphisms (SNPs) (e.g. *PNPLA3* rs738409, *TM6SF2* rs58542926 and *HSD17B13* rs72613567) correlated with susceptibility to NAFLD [3, 4, 5]. Many candidate genes have been investigated to depict the genetic background of NAFLD, including *SAMM50*.

*SAMM50* encodes Sam50, a mitochondrial outer membrane protein, and is of great significance in the removal of reactive oxygen species, mitochondrial morphology and regulation of mitophagy [6, 7, 8]. Previous studies have demonstrated that the rs738491 locus in *SAMM50* is one of the important SNPs strongly associated with NAFLD [9]. Kitamoto *et al*. [10] performed a GWAS in a Japanese cohort and identified several SNPs of *SAMM50*, including rs738491. These SNPs were correlated with the hepatic steatosis and cirrhosis, as well as NAFLD activity score. Chung *et al*. [11] reported other SNPs in different loci of *SAMM50* in a Korean population. However, the contribution of *SAMM50* polymorphisms to the occurrence and severity of fatty liver in the Chinese Han population has rarely been reported. The present study aims to determine whether *SAMM50* rs738491 and rs2073082 polymorphisms are associated with susceptibility to NAFLD in a Chinese cohort and to identify the mechanistic basis of this association. Accordingly, a case–control study was conducted in the well‐characterized Chinese Han population with NAFLD. In the *in vitro* part of the study, the function of *SAMM50* as a fatty acid metabolic regulator was assessed. The results revealed that a loss of *SAMM50* impaired fatty acid oxidation (FAO) and caused lipid accumulation within cells. These findings confirm the key role of *SAMM50* in lipid metabolism and provide a mechanistic basis for *SAMM50* polymorphisms associated with susceptibility to NAFLD.

## Materials and methods

### Subjects

In 2018, 380 patients and 380 healthy control subjects were recruited. Participants with viral hepatitis or other unrelated liver dysfunction were not included in this research. All eligible participants were examined through ultrasonographic examinations and denied alcohol abuse (male: < 140 g·week^−1^; female: < 70 g·week^−1^). Liver specimens were collected from patients undergoing liver biopsy. The study was approved by the Ethical Committees of the Shanghai Jiao Tong University, and all procedures were strictly compliant with the principles of the Declaration of Helsinki, and written informed consent has been obtained from each subject.

### Sample preparation and genomic DNA extraction

After fasting overnight, a total of blood samples of 5 mL were drawn from all participants for DNA extraction and biochemical assays, including aminotransferase (AST), aminotransferase (ALT), high‐density lipoprotein (HDL), low‐density lipoprotein (LDL) and triglyceride (TG), etc. Genomic DNA extraction was performed with a clot blood DNA kit (Beijing Cowin Biotech Co., Ltd, Beijing, China) in accordance with the manufacturer’s instructions.

### Genetic analysis

Genotyping of the two SNPs (rs738491 and rs2073082) was performed using the TaqMan SNP genotyping method. Details of the probes are presented in Table S1 (international serial numbers, gene names and chromosomal coordinates). An ABI ViiA™ 7 real‐time fluorescent quantitative PCR amplifier (Applied Biosystems, Foster City, CA, USA) was used for PCR amplification. The reaction comprised: denaturation step at 95 °C for 10 min and 40 cycles of denaturation at 95 °C for 15 s and annealing at 60 °C for 1 min; the samples contained 2.5 µL of 2 × TaqMan genotyping master mix, 5 pmol of 40 × TaqMan SNP genotyping assay and 20 ng of genomic DNA.

### Cell lines and cell culture

The hepatoma cell lines Hep3B, Huh7 and HepG2 were obtained from the American Type Culture Collection (ATCC, Manassas, VA, USA). QGY7703, L02, LM3, MHCC97H and Bel‐7402 cell lines were obtained from the laboratory of the Department of Clinical Pharmacology. Cells were cultured in Dulbecco’s modified Eagle’s medium (DMEM) containing 10% fetal bovine serum, with 1% of penicillin and streptomycin (NCM Biotech, Jiangsu, China) at 37 ºC in an incubator under 5% CO_2_.

### Plasmid constructs and generation of stable cell lines

Lentiviral vectors with a negative control (sh‐NC) and two shRNA‐SAMM50 were constructed. The targeting sequences were: negative control: 5ʹ‐AUCGAC CAGGACAUCACCUGCTT‐3ʹ; sh‐1#: 5ʹ‐AUAAGGUUCCUGUAGCCGACATT‐3ʹ; sh‐2#: 5ʹ‐CACACACCGUCUCUCGAGGAATT‐3’. The full length and shRNA‐resistant human *SAMM50* were inserted into the pLenti‐CMV‐3FLAG lentiviral vector. The empty pLenti‐CMV‐3FLAG lentiviral vector (Vector) was used as a control. All lentiviral vectors of *SAMM50* knockdown and overexpression were purchased from OBiO Technology (Shanghai, China). After attachment, the cells were transfected with lentivirus and screened using 2 μg·mL^−1^ puromycin for 7 days. The efficiency of *SAMM50* knockdown or overexpression was analyzed by immunoblotting.

### Preparation of fatty acid solution

The 5 mm stock solution of palmitic acid (PA) (Sigma‐Aldrich, St Louis, MO, USA) was obtained by dissolving PA in the DMEM medium containing 3% fatty acid‐free BSA with a water bath at 50 ºC . To prepare a 5 mm stock solution of PA and oleic acid (OA) (OA : PA) mixture (2 : 1; final concentration 5 mm), the sodium salt of palmitic acid was firstly dissolved in the DMEM containing 3% BSA and then sodium salt of OA was added [12]. The final concentration of 150 μm PA or 350 μm OA : PA mixture in 3% BSA was obtained by diluting the stock solution with DMEM.

### BODIPY 493/503 staining of neutral lipid droplets

The staining solution containing 2 μm BODIPY (D3922; Thermo Fisher Scientific, Waltham, MA, USA) was prepared in PBS. After PA or OA : PA treatment, cells were incubated with 4% paraformaldehyde for 15 min. Next, the fixed cells were incubated with 3 mL of staining solution for 15 min at 37 ºC in the dark. After washing in PBS, a drop of antifade polyvinylpyrrolidone mounting medium (Beyotime, Jiangsu, China) with 4ʹ,6‐diamidino‐2‐phenylindole was added onto the slides. Images were obtained with a fluorescence microscope (Leica, Wetzlar, Germany).

### Immunoblotting

Immunoblotting were performed as described previously [13]. The antibodies used were : SAMM50 (dilution 1 : 1000, ab133709; Abcam, Cambridge, UK), β‐tubulin (dilution 1 : 8000; 10094‐1‐AP; Protein Tech, Wuhan, China), CPT1A (dilution 1 : 1000; A5307, ABclonal, Wuhan, China), 3‐ketoacyl‐CoA thiolase (dilution 1 : 1000; A15778; ABclonal), enoyl‐CoA hydratase (dilution 1 : 1000; 66117‐1‐Ig; Protein Tech) and long‐chain specific acyl‐CoA dehydrogenase (dilution 1 : 1000; 17526‐1‐AP; Protein Tech).

### Cell viability test

Cells were seeded in a 96‐well plate (5000 cells per well) and tested for cell viability in the presence of various concentrations of PA or OA : PA as indicated. After incubation for 24 h, each well was supplemented with 10 µL of Cell Counting Kit‐8 solution (NCM Biotech, Jiangsu, China) for 2 h. The absorbance was measured at 450 nm using an Epoch 2 microplate reader (BioTek, Winooski, VT, USA).

### Quantitation of TG levels in Hep3B cells

After PA or OA : PA treatment, cells were lysed with a RIPA buffer for up to 20 min and centrifuged at 12 000 ***g*** for 20 min. A BCA kit was used for measuring the protein concentration of the supernatant and TG levels were detected using a TG kit (Nanjing Jiancheng, Jiangsu, China). The lipid content in Hep3B cells was expressed as mmol TG·g^–1^.

### Quantitative real‐time PCR (qRT‐PCR)

Total RNA of each sample was extracted by using RNAiso Plus reagent in accordance with the manufacturer’s instructions (Takara Bio, Shiga, Japan). The cDNA was generated from equal quantities of total RNA using PrimeScript™ RT master mix (Takara Bio). PCR was performed in a 30‐μL reaction system containing 6 μL of 5 × PrimeScript RT master mix and 6 μL of RNA supplemented with RNase‐free ddH_2_O made up to 30 μL. The reaction was performed on an ABI 7500 system. qRT‐PCR was performed using Hieff® qPCR SYBR® Green Master Mix (No Rox; Yeasen Biotech Co., Ltd, Shanghai, China) on a LightCycler® real‐time PCR system (Thermo Fisher Scientific) in accordance with the manufacturer’s instructions. The primers are listed in Table S2.

### Intracellular ketone body detection

Cells (approximately 18 × 10^6^) were collected by centrifugation (2000 ***g*** for 10 min at 4 °C). The cell pellet was resuspended in 1–2 mL of cold assay buffer. The cell suspension was sonicated 20× at bursts of 1 s and centrifuged at 10 000 ***g*** for 10 min at 4 °C. The supernatant was removed and stored on ice. After sample preparation, the colorimetric assay kits [β‐hydroxybutyrate (β‐HB), ab83390, Abcam; acetoacetate (AcAc), ab180875, Abcam] were used in accordance with the manufacturer’s instructions. The absorbance at 450 or 550 nm was recorded using a microplate spectrophotometer (BioTek).

### Bioinformatics analyses

Two expression microarrays, GSE130970 and GSE126848, containing liver samples with or without steatosis were available in the GEO database (https://www.ncbi.nlm.nih.gov/geo).

### Mitochondrial complex activity assay

In total, 5 × 10^6^ cells from each group (Sh‐NC, Sh‐1# and Sh‐2#) were used for mitochondria isolation using the Cell Mitochondria Isolation Kit (Beyotime). Next, the activities of mitochondrial complex I–IV were evaluated using a Mitochondrial Respiratory Chain Complex I–IV Activity Assay Kit (Solarbio, Beijing, China) in accordance with the manufacturer’s instructions. The activity of mitochondrial complex I–IV was expressed as μmol/(min × mg).

### Immunohistochemistry

The paraffin‐embedded liver tissue was deparaffinized with standard protocols and rehydrated. After antigen retrieval, the tissue was blocked and incubated with the anti‐SAMM50 (dilution 1 : 500) antibody overnight at 4 °C. Then, the slides were washed and incubated with biotin‐labeled goat anti‐mouse IgG (H + L) at 37 °C for 15 min and developed with 3,3ʹ‐diaminobenzidine work solution. Images were obtained using a Leica microscope.

### Palmitate‐driven oxygen consumption rate (OCR)

An equal amount of Hep3B cells (6000 per well) were seeded in a 96‐well plate and cultured in the substrate‐limited growth media [glucose 0.5 mm, glutamine or GlutaMAX 1.0 mm, serum (e.g. fetal bovine serum) 1.0%, XF l‐carnitine 0.5 mm] for 12 h. Before initiation, the media was substituted with substrate‐limited assay media (XF glucose 2.0 mm, XF l‐carnitine 0.5 mm) and the cell plate was placed in non‐CO_2_, 37 °C incubator for 60 min. Then, the rate of O_2_ changes in the immediate vicinity of the cells was analyzed using a Seahorse XFe96 Analyzer (Agilent Technologies, Santa Clara, CA, USA). The rates were calculated based on the ratio of concentration changes versus time measured during continuous 3‐min measurement periods followed by 3 min of mixing and 2 min of incubation. Injectors deliver the inhibitors (oligomycin 2 μm, FCCP 2 μm, and R/A 1.2 μm) into the chamber followed by mixing for 3 min and measuring for 3 min. The levels of O_2_ consumption rate in the presence of palmitate were considered as the capacity of intracellular FAO.

### Statistical analysis

Hardy–Weinberg equilibrium was examined using a chi‐squared test. A Student’s *t*‐test or the Mann–Whitney *U*‐test were used to evaluate the statistical difference between two groups. A chi‐squared test was performed to compare categorical variables. Differences with respect to quantitative clinical data in patients with different genotypes were analyzed by the Kruskal–Wallis test followed by the post‐hoc Dunn’s test. *P* < 0.05 was considered statistically significant. In *in vitro* studies, Student’s *t*‐test was applied to analyze the difference between two groups. For more than two groups, one‐way analysis of variance (ANOVA) followed by a least significant difference (LSD) test was applied. Statistical analyses were performed using spss, version 22.0 (IBM Corp., Armonk, NY, USA).

## Results

### Baseline characteristics of NAFLD patients and normal controls

The demographic and clinical characteristics of NAFLD patients and normal controls are presented in Table 1. Compared with the normal controls, NAFLD patients had significantly higher levels of serum TG, LDL, ALT, AST and alkaline phosphatase (AKP) (all *P* < 0.05). However, no significant differences were observed between the two groups in terms of body mass index (BMI), total cholestrol (TC), HDL, blood glucose (GLU) and glycated albumin (GA) (all *P* > 0.05, Table 1).

**Table 1:** Demographic and clinical characteristics of NAFLD patients and normal controls. SBP, systolic blood pressure; DBP, diastolic blood pressure; TBIL, total bilirubin; γ‐GT, gamma glutamyltransferase. Data are given as the mean ± SD or as the number of cases

	NAFLD (*n* = 380)	Normal controls (*n* = 380)	*P*
Gender (male/female)	226/154	224/156	0.883^a^
Age (years)	45.6 ± 13.1	45.3 ± 13.4	0.745
BMI (kg m^−2^)	23.3 ± 1.5	23.0 ± 2.4	0.065
SBP (mmHg)	116.2 ± 13.0	115.89 ± 13.2	0.718
DBP (mmHg)	76.3 ± 8.6	76.0 ± 8.5	0.554
TC (mmol·L^−1^)	4.2 ± 1.7	4.0 ± 1.7	0.236
TG (mmol·L^−1^)	1.7 ± 1.0	1.3 ± 0.6	0.000*
HDL (mmol·L^−1^)	1.4 ± 0.3	1.4 ± 0.3	0.703
LDL (mmol·L^−1^)	3.1 ± 1.0	2.8 ± 0.9	0.001*
ALT (IU·L^−1^)	26.2 ± 11.8	23.1 ± 10.6	0.000*
AST (IU·L^−1^)	27.8 ± 12.1	24.9 ± 10.1	0.000*
TBIL (μmol·L^−1^)	11.2 ± 4.6	11.3 ± 5.1	0.669
DBIL (μmol·L^−1^)	2.9 ± 1.2	2.9 ± 1.5	0.635
γ‐GT (IU·L^−1^)	37.9 ± 28.4	34.3 ± 27.3	0.071
AKP(IU·L^−1^)	84.6 ± 27.1	79.2 ± 25.6	0.005*
GLU (mmol·L^−1^)	5.1 ± 0.8	5.1 ± 0.7	0.084
GA (%)	13.8 ± 2.0	13.6 ± 1.8	0.207

* *P* < 0.05 indicates statistical significance.

^a^ Statistical significance was determined by chi‐squared analysis, as well as Student’s *t*‐test or the Mann–Whitney *U*‐test based on variable types.

### Allele and genotype frequency analysis

Allelic frequencies of *SAMM50* polymorphisms (rs738491 and rs2073082) were in Hardy–Weinberg equilibrium in both NAFLD and control groups (Table 2). The genotypes were evaluated in all individuals using the TaqMan SNP genotyping method. As summarized in Table 2, the T‐allele frequency of rs738491 and the G‐allele frequency of rs2073082 were more prevalent in the NAFLD group compared to those in the control group [T: 52.3% > 44.0%, odds ratio (OR) = 1.39, 95% confidence interval (CI) = 1.14–1.71, *P* = 0.001; G: 71.2% > 65.4%, OR = 1.31, 95% CI = 1.05–1.62, *P* = 0.016]. These results are consistent with a previous study performed in a Chinese Han population in 2015 [14]. Furthermore, the proportion of NAFLD patients in cohorts with different genotypes was compared using the Cochran–Armitage trend test. As shown in Table 3, compared with the CC genotype of rs738491, the proportion of NAFLD patients in the genotype TT or CT cohort was significantly higher. The OR exhibits a dose‐dependent effect at the rs738491 locus, indicating that each copy of rs738491 T allele increases the risk of fatty liver (TT, OR = 1.97, 95% CI = 1.30–2.98; CT, OR = 1.38, 95% CI = 0.98–1.96; *P* = 0.001). Similar findings were obtained when comparing the OR of GG or AG versus AA at rs2073082 locus (GG, OR = 1.60, 95% CI = 0.96–2.65; AG, OR = 1.14, 95% CI = 0.69–1.90; *P* = 0.015). Also, chi‐squared analysis showed that subjects carrying T allele of rs738491 were more likely to have NAFLD (TT + CT vs. CC, OR = 1.54, 95% CI = 1.11–2.14, *P* = 0.009). However, the correlation between the G‐allele carriers of rs2073082 and the occurrence of NAFLD was not statistically significant (GG + AG vs. AA, OR = 1.36, 95% CI = 0.84–2.20, *P* = 0.216).

**Table 2:** Correlation between frequencies of allele at loci rs738491 and rs2073082 and the risk of NAFLD. HWE, Hardy–Weinberg; NC, normal controls

	Groups	Cases, *n*	HWE, *P*‐value	Frequency of allele		*P*
				*C* (%)	*T* (%)	
rs738491	NAFLD	372	0.725	355 (47.7)	389 (52.3)	0.001*
	NC	378	0.625	423 (56.0)	333 (44.0)	
	OR (95% CI)			1.00 (Ref)	1.39 (1.14–1.71)	

	Groups	Cases, *n*	HWE, *P*‐value	Frequency of allele		*P*
				A (%)	G (%)	
rs2073082	NAFLD	378	0.887	218 (28.8)	538 (71.2)	0.016*
	NC	377	0.521	261 (34.6)	493 (65.4)	
	OR (95% CI)			1.00 (Ref)	1.31 (1.05–1.62)	

Data are presented as proportions [*n*, (%)] of *SAMM50* allelic frequency, *P*‐values were determined by a chi‐squared test.

* *P* < 0.05 indicates statistical significance.

**Table 3:** Association between rs738491 and rs2073082 genotypes and the risk of NAFLD

SNP	Genotypes	NAFLD	Controls	OR	*P*
		*n* (%)	*n* (%)	(95% CI)	
rs738491		372	378		
	CC	83 (41.7)	116 (58.3)	1.00 (Ref)	0.001*
	CT	189 (49.7)	191 (50.3)	1.38 (0.98–1.96)	
	TT	100 (58.5)	71 (41.5)	1.97 (1.30–2.98)	
	TT + CT vs. CC			1.54 (1.11–2.14)	0.009^a^
rs2073082		378	377		
	AA	32 (43.2)	42 (56.8)	1.00 (Ref)	0.015*
	AG	154 (46.5)	177 (53.5)	1.14 (0.69–1.90)	
	GG	192 (54.9)	158 (45.1)	1.60 (0.96–2.65)	
	GG + AG vs. AA			1.36 (0.84–2.20)	0.216^a^

Data are presented as proportions [*n*, (%)] of *SAMM50* genotypes (rs738491 and rs2073082).

*P* < 0.05 indicates statistical significance.

* *P*‐values were determined by the Cochran–Armitage trend test comparing the mild fatty liver group with the moderate to severe fatty liver group.

^a^ Statistical significance was determined by chi‐squared analysis.

### Clinical and biochemical features of NAFLD patients with different genotypes of the rs738491 and rs2073082 SNPs

As summarized in Table 4, patients with different rs738491 genotypes showed significant differences in TG, LDL, ALT and AST levels (all *P* < 0.05). Dunn’s test was additionally performed for multiple comparisons of genotypes to determine the pattern of the differences. The result showed the TG levels in patients carrying the TT or CT genotype were higher than in those carrying the CC genotype (CC vs. CT; TT vs. CC, all *P* < 0.05) (Table 4). The serum levels of LDL, ALT and AST in patients carrying the TT or CC genotypes were also compared and these parameters were higher in patients with TT genotypes. Among these parameters, some were also higher in those with the heterozygous CT genotype compared with the CC homozygous genotype (e.g. TG and ALT) (Table 4). Similarly, serum TG, AST, ALT and direct bilirubin (DBIL) levels were different among rs2073082 genotypes (all *P* < 0.05) (Table 4), with all parameters being higher (except for DBIL) in the GG genotype group. The results from multiple comparisons showed that serum AST was significantly elevated in patients carrying the GG genotype compared to those with the AA genotype (Table 4). These results suggest that the genotypes of rs738491 (TT and CT) and rs2073082 (GG) are associated with higher levels of TG, ALT and AST, implying that abnormal lipid metabolism and liver dysfunction were related to patients with *SAMM50* polymorphisms.

**Table 4:** Correlation between genotypes at loci rs738491and rs2073082, as well as baseline clinical information in the NAFLD group. TBIL, total bilirubin; γ‐GT, gamma glutamyltransferase.

	rs738491				rs2073082			
	Genotypes			*P*	Genotypes			*P*
	CC	CT	TT		AA	AG	GG	
Cases	83	189	100		32	154	192	
Gender (male/female)	46/37	118/71	60/40	0.553 ^a^	22/10	93/61	109/83	0.413^a^
Age (years)	44.1 ± 13.5	45.8 ± 13.7	46.3 ± 11.7	0.413	42.6 ± 14.0	44.3 ± 13.4	47.1 ± 12.6	0.038*
BMI (kg m^−2^)	23.2 ± 1.3	23.4 ± 1.7	23.2 ± 1.2	0.572	23.1 ± 1.6	23.4 ± 1.5	23.2 ± 1.5	0.342
SBP (mmHg)	115.1 ± 14.8	117.2 ± 12.8	114.9 ± 11.9	0.246	120.3 ± 15.5	115.9 ± 12.8	115.9 ± 12.8	0.220
DBP (mmHg)	75.3 ± 8.5	76.8 ± 8.5	75.8 ± 8.0	0.285	77.8 ± 8.7	75.9 ± 8.2	76.5 ± 8.9	0.427
TC (mmol·L^−1^)	3.9 ± 1.7	4.2 ± 1.7	4.3 ± 1.8	0.201	3.8 ± 1.7	4.2 ± 1.7	4.2 ± 1.7	0.414
TG (mmol·L^−1^)	1.5 ± 1.0^a^	1.7 ± 1.0	1.8 ± 1.0^c^	0.009*	1.5 ± 1.0	1.6 ± 0.8	1.8 ± 1.1	0.035*
HDL (mmol·L^−1^)	1.3 ± 0.3	1.4 ± 0.3	1.4 ± 0.3	0.133	1.3 ± 0.3	1.4 ± 0.3	1.4 ± 0.3	0.063
LDL (mmol·L^−1^)	2.8 ± 0.9	3.0 ± 1.0^b^	3.3 ± 1.0^c^	0.001*	2.8 ± 1.0	3.0 ± 1.0	3.1 ± 1.0	0.136
ALT (IU·L^−1^)	21.8 ± 7.7^a^	26.4 ± 11.7	29.0 ± 13.8^c^	0.000*	22.9 ± 8.6	25.0 ± 11.3	27.7 ± 12.5	0.034*
AST (IU·L^−1^)	24.8 ± 9.1	27.7 ± 11.3	31.2 ± 14.8^c^	0.013*	22.4 ± 8.6	27.3 ± 11.1	29.3 ± 13.0^c^	0.011*
TBIL (μmol·L^−1^)	10.5 ± 4.2	11.4 ± 5.0	11.3 ± 4.2	0.506	10.4 ± 4.4	10.8 ± 4.8	11.6 ± 4.5	0.191
DBIL (μmol·L^−1^)	2.8 ± 1.1	3.0 ± 1.3	2.7 ± 1.1	0.152	2.6 ± 1.0	3.1 ± 1.2^b^	2.7 ± 1.2	0.003*
γ‐GT (IU·L^−1^)	33.0 ± 19.8	40.0 ± 29.3	39.2 ± 33.0	0.145	32.8 ± 18.1	35.8 ± 27.1	40.2 ± 30.6	0.462
AKP (IU·L^−1^)	86.2 ± 28.0	82.6 ± 26.5	87.4 ± 27.2	0.314	90.1 ± 26.0	84.0 ± 26.2	83.9 ± 28.0	0.407
GLU (mmol·L^−1^)	5.0 ± 0.8	5.2 ± 0.8	5.2 ± 1.0	0.279	5.0 ± 0.8	5.1 ± 0.8	5.3 ± 0.9	0.101
GA (%)	13.5 ± 1.6	13.6 ± 1.8	14.2 ± 2.6	0.156	13.6 ± 1.2	13.6 ± 1.9	13.8 ± 2.3	0.845

Data are presented as the mean ± SD of *SAMM50* genotypes (rs738491 and rs2073082).

* *P* < 0.05 indicates statistical significance.

^a^ Statistical significance was determined by chi‐squared analysis, as well as by the Kruskal–Wallis test with Dunn’s test for multiple comparisons. Statistical significance is indicated (^a^C/C vs. C/T in rs738491, A/A vs. A/G in rs2073082, ^b^C/T vs. T/T in rs738491, A/G vs. G/G in rs2073082, ^C^C/C vs. T/T in rs738491, A/A vs. G/G in rs2073082.

### Associations between *SAMM50* polymorphisms and severity of NAFLD

To assess whether the *SAMM50* gene polymorphisms exhibited association with the severity of NAFLD, we divided the NAFLD patients into two groups (mild fatty liver and moderate to severe fatty liver) (Table 5) according to ultrasonic diagnostic criteria and compared the proportion of moderate to severe fatty liver among the different genotypes. As summarized in Table 5, rs738491 genotypes demonstrated a strong association with the severity of fatty liver and, with an increase in the number of T alleles, the risk of moderate to severe fatty liver increased (TT, OR = 3.08, 95% CI = 1.67–5.68; CT, OR = 2.11, 95% CI = 1.22–3.65; *P* < 0.001) (Table 5). Additionally, T‐allele carriers had a higher risk of moderate to severe fatty liver (TT + CT vs. CC, OR = 2.40, 95% CI = 1.42–4.05, *P* = 0.001) in the NAFLD cohort. Similar results were noted in the rs2073082 locus in terms of the association with the severity of fatty liver (GG, OR = 2.61, 95% CI = 1.15–5.93; AG, OR = 2.02, 95% CI = 0.88–4.65; *P* = 0.021) (Table 5). Additionally, a higher risk of having moderate to severe fatty liver was seen in patients carrying the G allele (GG + AG vs. AA, OR = 2.33, 95% CI = 1.04–5.18, *P* = 0.034) (Table 5). Meanwhile, rs738491 T‐allele carriers (TT + CT) had higher levels of ALT and AST (all *P* < 0.05) (Table S3). Patients harboring the G allele (GG + AG) of rs2073082 only had a higher serum AST level (AST, GG + AG: 28.4 ± 12.3 IU·L^−1^; AA: 22.4 ± 8.6 IU·L^−1^, *P* < 0.05) (Table S3). However, G‐allele carriers and noncarriers showed no differences in ALT level. In addition, we also noted that being a risk allele carrier (T or G alleles) did not affect the levels of AST and ALT in the healthy control group or all participants as compared to noncarriers (Table S3). Taken together, these results suggest that *SAMM50* rs738491 and rs2073082 were highly correlated with the severity of NAFLD.

**Table 5:** Association between the genotypes of rs738491 and rs2073082 and the severity of NAFLD. Ultrasonic diagnosis criteria were: (1) diffuse enhancement of near‐field echo in the hepatic region (stronger than in the kidney and spleen region) and gradual attenuation of the far‐field echo; (2) unclear display of the intra‐hepatic lacuna structure; (3) presentation of mild to moderate hepatomegaly with a round and blunt border; (4) color Doppler ultrasonography shows reduced blood flow in the liver, which may be difficult to detect, but the distribution of blood flow is normal; and (5) unclear or non‐intact display of the envelope of the right liver lobe and diaphragm. Patients with a mild degree of fatty liver disease demonstrate item 1 and any one of items 2–4; patients with moderate fatty liver disease demonstrate item 1 and any two of items 2–4; patients with severe degree of fatty liver disease demonstrate items 1 and 5 and any two of items 2–4. Data are presented as proportions [*n*, (%)] of *SAMM50* genotypes (rs738491 and rs2073082). *P*‐values were determined by the Cochran–Armitage test comparing the mild fatty liver group with the moderate to severe fatty liver group

SNPs	Genotype	Mild fatty liver, *n* (%)	Moderate to severe fatty liver, *n* (%)	OR (95% CI)	*P*
rs738491	CC	58 (69.9%)	25 (30.1%)	1.00 (Ref)	< 0.001
	CT	99 (52.4%)	90 (47.6%)	2.11 (1.22–3.65)	
	TT	43 (43.0%)	57 (57.0%)	3.08 (1.67–5.68)	
	TT + CT vs. CC			2.40 (1.42–4.05)	0.001^a^
rs2073082	AA	23 (71.9%)	9 (28.1%)	1.00 (Ref)	0.021
	AG	86 (55.8%)	68 (44.2%)	2.02 (0.88–4.65)	
	GG	95 (49.5%)	97 (50.5%)	2.61 (1.15–5.93)	
	GG + AG vs. AA			2.33 (1.04–5.18)	0.034^a^

* *P* < 0.05 indicates statistical significance.

^a^ Statistical significance was determined by chi‐squared analysis.

### SAMM50 deficiency enhances lipid accumulation in cells

**Fig. 1:**
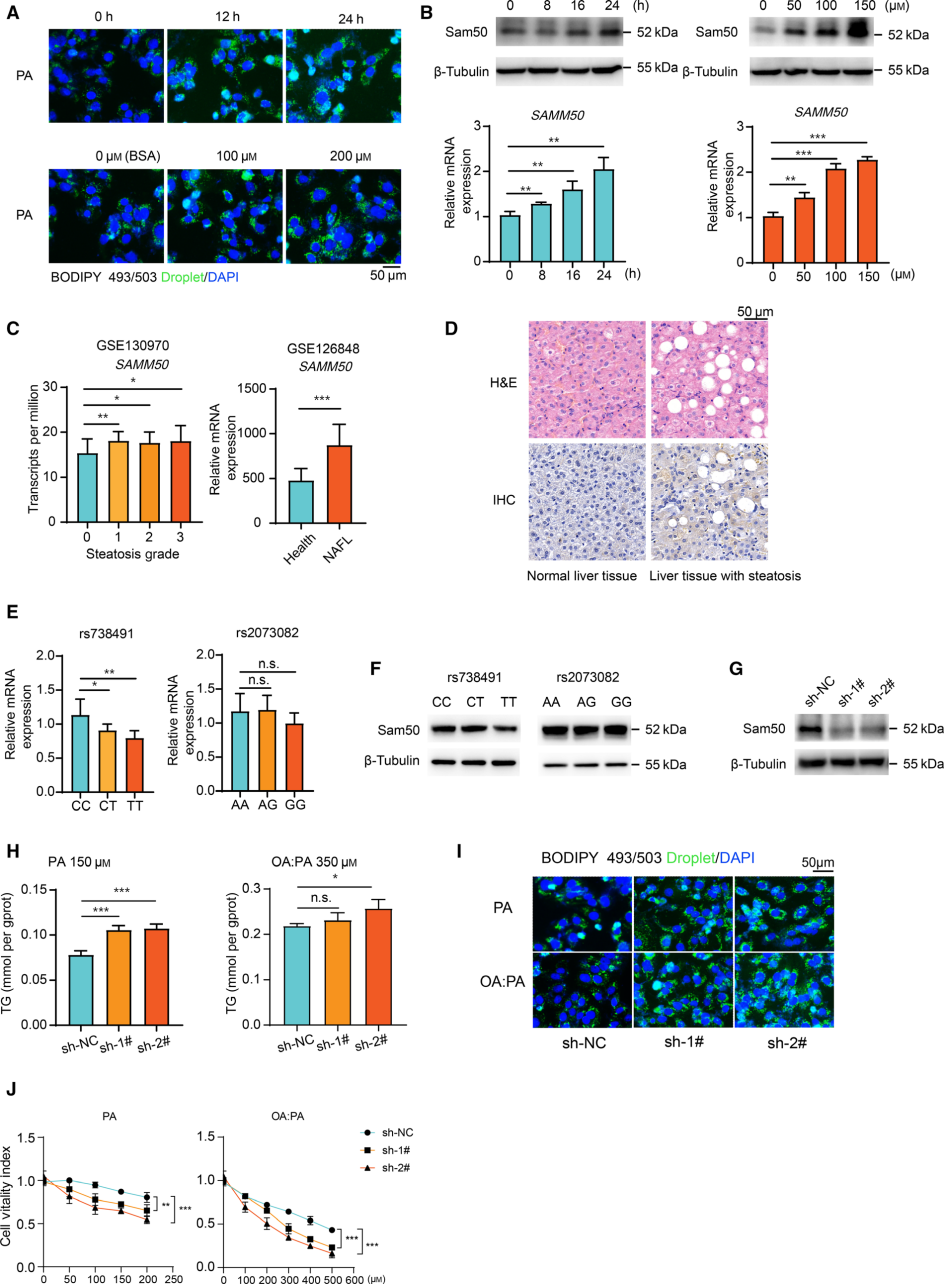
*SAMM50* knockdown caused lipid accumulation in human hepatoma cells under fatty acid treatment. (A) Staining of lipid droplets (green) by BODIPY 493/503 in Hep3B cells with PA stimulation. Scale bars = 50 μm. (B) Protein and mRNA levels (*n* = 3) of *SAMM50* in Hep3B cells after PA treatment. (C) The mRNA levels of *SAMM50* in patients with or without hepatic steatosis from the GEO database. (D) Representative photos of immunohistochemical staining with *SAMM50* on liver tissues with or without steatosis. Scale bars = 50 μm. (E) Hepatic mRNA levels of *SAMM50* among genotypes (CC = 8, CT = 5, TT = 4; AA = 8, AG = 5, GG = 6). (F) Hepatic *SAMM50* protein levels among genotypes were detected by immunoblotting. (G) Verification of *SAMM50* knockdown by immunoblotting. (H,I) Intracellular TG content in *SAMM50* knockdown cells with fatty acid stimulation for 24 h. Scale bars = 50 μm. (J) The cell viabilities are compared between *SAMM50* knockdown and control groups after fatty acid stimulation (*n* = 5). Statistical analyses were performed using Student’s *t*‐test between two groups or one‐way ANOVA followed by a LSD test for more than two groups. Data are expressed as the mean ± SD, *P < 0.05, **P < 0.01, ***P < 0.001, n.s., not significant

Consistent with previous studies [11], our result further confirm that *SAMM50* polymorphisms (rs738491 and rs2073082) are associated with an elevated risk of NAFLD. However, the mechanistic basis of this association is poorly defined. To this aim, Hep3B cells stimulated with PA or a mixture of PA and OA (OA : PA) were used to investigate the endogenous change of *SAMM50* because these *in vitro* models are known to mimic NAFLD *in vivo* [15]. The cells demonstrated increased lipid accumulation in a time‐ and dose‐dependent manner after PA stimulation (Fig. 1A). Concomitant to lipid accumulation, PA stimulation markedly enhanced *SAMM50* expression at the protein and mRNA levels (Fig. 1B). Similarly, the RNA‐seq data (GSE130970 and GSE126848) from the GEO database confirmed that the transcript levels of *SAMM50* were higher in NAFLD patients (Fig. 1C). Immunohistochemistry (IHC) staining analysis indicated that *SAMM50* expression has an upregulation trend in the liver of patients with steatosis compared with that in the normal liver (Fig. 1D). Taken together, these results suggest lipid overload would cause the upregulation of *SAMM50* expression.

To explore the impact of gene polymorphisms on *SAMM50* expression, we found lower mRNA levels of *SAMM50* in subjects harboring a homozygous TT genotype of rs738491 and a GG genotype of rs2073082 (Fig. 1E), which was also confirmed by the protein levels in the liver of patients carrying the TT genotype (Fig. 1F, left), although *SAMM50* protein expression in the GG genotype was not significantly different from that in the AA genotype (Fig. 1F, right). To explore the effect of *SAMM50* deficiency on intracellular lipid accumulation, a cell line with the highest expression of *SAMM50*, Hep3B, was selected (Fig. S1A and S1B). *SAMM50* knockdown Hep3B cells were generated using *SAMM50*‐targeting shRNAs (sh‐1#, sh‐2# and sh‐NC as a control) (Fig. 1G and S1C). We found that the intracellular TG content was higher in *SAMM50* knockdown Hep3B cells treated with PA or OA : PA (Fig. 1H). BODIPY 493/503 staining of neutral lipid droplets demonstrated that knockdown of *SAMM50* increased lipid content in Hep3B cells (Fig. 1I). Additionally, cell viability assay showed that *SAMM50* knockdown caused fatty acid intolerance after PA or OA : PA treatment (Fig. 1J) because lipid overload within cells would induce endoplasmic reticulum stress and cause subsequent cell death [16]. Overall, these results reveal that the expression of *SAMM50* is upregulated in fatty liver and that *SAMM50* knockdown enhances lipid accumulation in cells.

### Decreased FAO in *SAMM50* knockdown cells

**Fig. 2:**
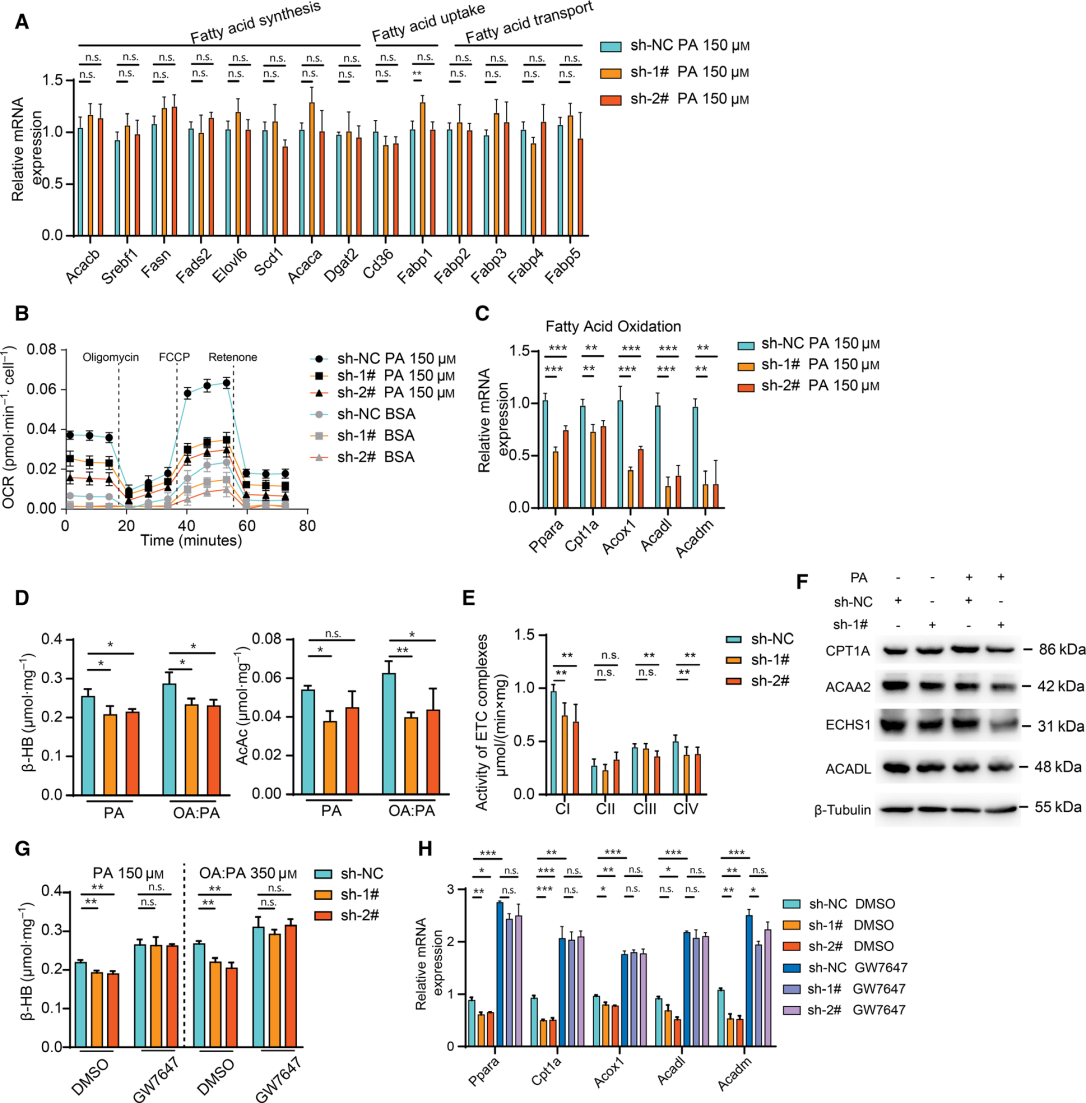
FAO is impaired as a result of *SAMM50* deficiency. (A) The mRNA levels of the fatty acid metabolism‐related genes in both *SAMM50* knockdown and control groups (*n* = 3). Gene expression levels were normalized for Actb. (B) The OCR using palmitate (or BSA) as the only substrate was measured in *SAMM50* knockdown and control groups (*n* = 6). (C) The expression of FAO‐related genes in both *SAMM50* knockdown and control groups after PA treatment. (D) Levels of ketone bodies (β‐HB and AcAc) within cells were tested and normalized to protein levels in *SAMM50* knockdown cells after PA stimulation (*n* = 3). (E) The activity of electron transport chain complexes was compared between *SAMM50* knockdown and control groups. (F) The levels of mitochondrial proteins involved in β‐oxidation were measured in *SAMM50* knockdown group with or without PA treatment. (G,H) The β‐HB levels and the expression of FAO‐related genes between the two groups are shown after GW7647 (10 μm) treatment. Statistical analyses were performed using one‐way ANOVA followed by a LSD test. Data are expressed as the mean ± SD, *P < 0.05, **P < 0.01, ***P < 0.001, n.s., not significant

Intrahepatic TG accumulation occurs partially as a result of the imbalance of fatty acid uptake, synthesis and insufficient disposal (e.g. FAO) [17]; however, it is not clear which metabolic process changes in the absence of *SAMM50*. NAFLD is characterized in part by an excessive accumulation of TG in cells (also known as hepatic steatosis) as a result of enhanced hepatic fatty acid synthesis. Several genes are involved in this process (e.g. *Acacb, Srebf1, Fasn*, etc.). However, our results indicated that the expression of these genes was not influenced by *SAMM50* deficiency after PA treatment, nor did genes involved in fatty acid uptake [18, 19], such as *Cd36* and *Fabp1* (Fig. 2A). *Fabps* (*Fabp2, Fabp3, Fabp4* and *Fabp5*) play a key role in the intracellular transport of long‐chain fatty acids (such as PA or OA) and their acyl‐CoA esters [20, 21]. Nevertheless, the expression of *Fabps* was unchanged in *SAMM50* knockdown cells (Fig. 2A). Taken together, these findings indicate that that *SAMM50* deficiency is unlikely to promote intracellular lipid accumulation by enhancing these processes.

**Fig. 3:**
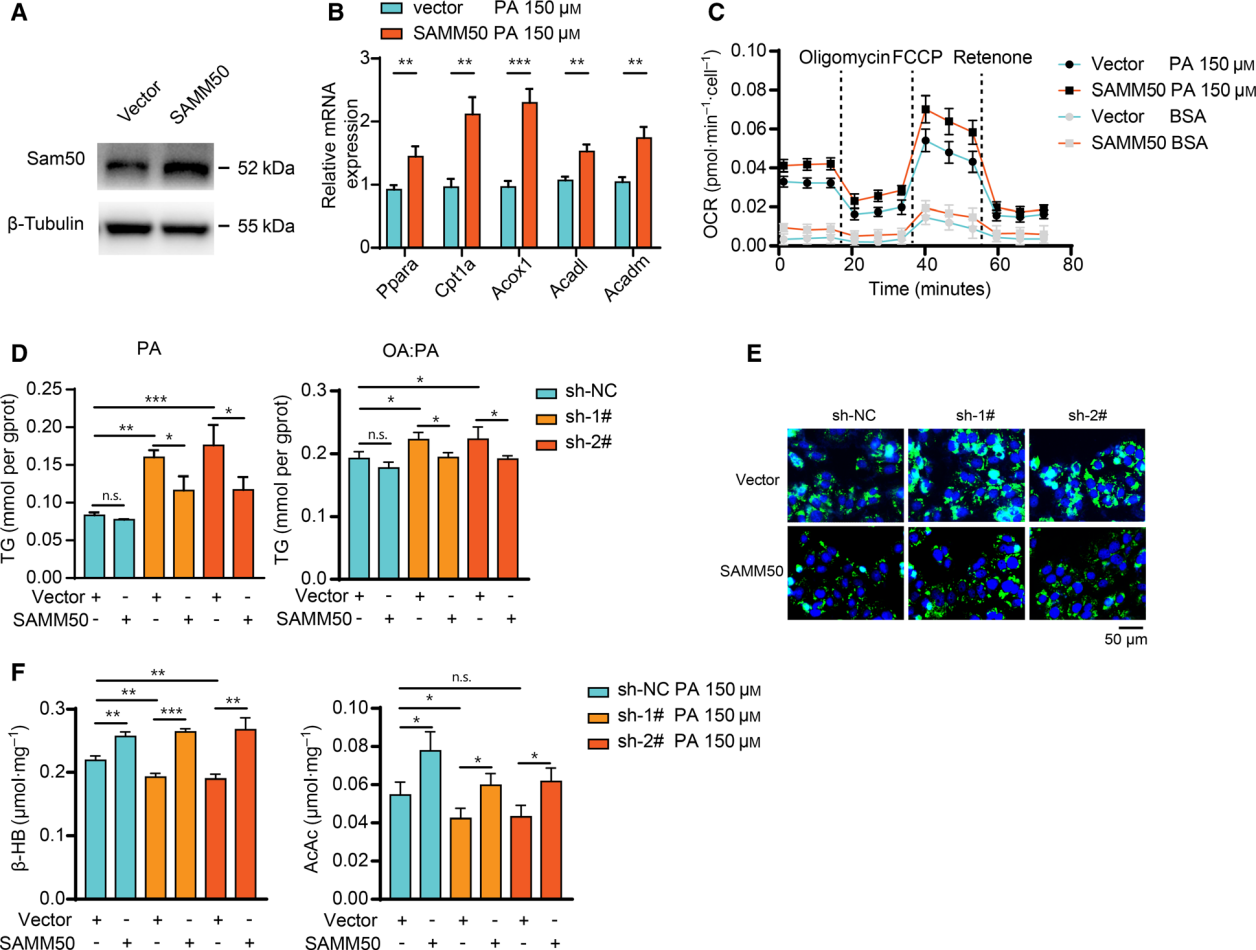
Overexpression of *SAMM50* mitigates lipid accumulation and enhances FAO. (A) Overexpression of *SAMM50* was verified by immunoblotting. (B) Overexpression of *SAMM50* increased the expression of FAO‐related genes after PA stimulation. (C) Overexpression of *SAMM50* increased the palmitate‐dependent OCR in Hep3B cells. (D,E) Overexpression of *SAMM50* in *SAMM50* knockdown cells mitigates lipid accumulation, as indicated by staining of lipid droplets with BODIPY 493/503 and intracellular TG detection. Scale bars = 50 μm. (F) Overexpression of *SAMM50* elevated the levels of ketone bodies (β‐HB and AcAc) in *SAMM50* knockdown cells after PA challenge for 48 h, *n* = 3. Statistical analyses were performed using one‐way ANOVA followed by a LSD test. Data are expressed as the mean ± SD, *P < 0.05, **P < 0.01, ***P < 0.001, n.s., not significant

FAO, a significant process of energy output, usually occurs in mitochondria when there is excessive fatty acid [22, 23]. *SAMM50* is indispensable with respect to maintaining the structure of mitochondrial cristae for proper function. Therefore, we determined whether FAO is disrupted in a *SAMM50*‐deficient condition. The OCR with palmitate as substrate was measured to assess the performance of FAO in *SAMM50* knockdown cells. As shown in Fig. 2B, *SAMM50* knockdown cells had lower palmitate‐dependent OCR than the NC group. The expression of relevant genes in FAO (*Ppara, Cpt1a*, *Acox1, Acadl* and *Acadm*) was decreased in *SAMM50* knockdown cells when treated with PA or OA : PA for 48 h (Fig. 2C and S2A). The levels of ketone bodies (β‐HB and AcAc), the surrogate marker of FAO [24], were also decreased after fatty acid challenge (Fig. 2D). In addition, the activity of the electron transport chain complexes was compromised to some extent (Fig. 2E). We also noted that the levels of mitochondrial proteins involved in β‐oxidation were also lower in the *SAMM50* knockdown group after PA treatment (Fig. 2F). These results indicate that the decrease of FAO level caused by *SAMM50* knockdown is partially a result of mitochondrial disfunction. Furthermore, the decreased expression of FAO‐related genes and the lower levels of ketone bodies in *SAMM50* knockdown cells could be reversed after using GW7647, the agonist of PPARα (Fig. 2G, S2B and 2H) because PPARα activation would enhance the FAO process. Moreover, overexpression of *SAMM50* increased the expression of FAO‐related genes (Fig. 3A, 3B and S2C), as well as the palmitate‐dependent OCR levels (Fig. 3C). We also found that *SAMM50* overexpression reduced intracellular lipid accumulation in *SAMM50* knockdown cells (Fig. 3D,E) and elevated ketone body levels (Fig. 3F and S2D) in the PA‐ or OA : PA‐induced lipid accumulation model. Taken together, we concluded that *SAMM50* deficiency decreased the FAO process and caused lipid accumulation.

## Discussion

NAFLD is a general pathology that comprises a continuum of liver diseases varying from mild liver injury to serious inflammatory processes, such as non‐alcoholic steatohepatitis, which result in fibrosis and even NAFLD‐related hepatocellular carcinoma [25]. By 2030, the number of NAFLD cases is expected to reach 100.9 million [26]. Overnutrition and sedentary lifestyle have been considered as the main causes of NAFLD [27] and genetic variants, including *PNPLA3* rs738409, *TM6SF2* rs58542926 and *HSD17B13* rs72613567, in patients may accelerate or decelerate the progression of NAFLD [28, 29, 30]. The present study focused on another candidate gene, *SAMM50*, aiming to investigate its association with NAFLD in a Chinese cohort (Table S4). Our results indicated that the T allele of rs738491 or G allele of rs2073082 was highly correlated with the occurrence and progression of NAFLD. Additionally, our data showed that patients with the T allele or G allele had elevated serum TG, AST and ALT levels, suggesting that genetic variants of *SAMM50* are associated with abnormal lipid metabolism and liver injury.

Previous studies have reported that the T allele of rs738491 showed a strong association with NAFLD in a Japanese population [10], which is in agreement with our results. However, T‐allele carriers had a decreased level of serum TG in the Japanese cohort. This finding is opposite to our results, which may be a result of differences in the populations or the phenomenon that SNPs in the other gene had a stronger impact on TG levels than the SNP in the *SAMM50* gene. Further investigations in a large cohort are warranted to address this discrepancy. In addition, it should be noted that rs738491 T‐allele carriers tend to have a risk of moderate to severe fatty liver disease in terms of NAFLD. These results demonstrate that rs738491 may play an essential role in the progression of NAFLD. Furthermore, the G allele of rs2073082 is also associated with a higher incidence of NAFLD. To the best of our knowledge, this is the first report to evaluate the association of rs2073082 locus with NAFLD in the Chinese Han population. Our results suggest an association between the rs2073082 G allele and the occurrence and progression of NAFLD, and also that the carriers had higher levels of serum TG and AST.

Although *SAMM50* is implicated in the risk of NAFLD, the underlying mechanisms are poorly defined. The *SAMM50* gene, which encodes Sam50, is critical for the stability of mitochondrial structure and the function of mitochondria [31]. Therefore, we hypothesized that *SAMM50* deficiency as a result of genetic change may be involved in mitochondrial dysfunction. Experiments conducted *in vitro* showed that the expression of FAO‐related genes was significantly lower in *SAMM50* knockdown cells. The OCR is an important index for directly assessing the ability of FAO using palmitate as the only substrate. Our results showed that this rate was reduced in *SAMM50* knockdown groups. Meanwhile, the result of FAO is the production of ketone bodies, including β‐HB and AcAc. Therefore, the ketone bodies produced from cells were measured, confirming that *SAMM50* knockdown cells did produce less ketone bodies. Additionally, overexpression of *SAMM50* reversed the phenotype caused by *SAMM50* deficiency, which further bolsters our hypothesis. Taken together, these results suggest a pivotal role of *SAMM50* in FAO, and the loss of the gene would impair FAO and cause lipid accumulation in cells.

Except for FAO, we noted no significant differences in terms of the expression of fatty liver synthesis‐, uptake‐ and transport‐related genes after *SAMM50* knockdown. Identification of the changed processes involved in fatty acid metabolism in *SAMM50* knockdown cells is essential for understanding of the occurrence of NAFLD because the liver is the pivotal hub for lipid metabolism. The input of fatty acids did not increase; however, disposal was impaired. The imbalance between the input and output behavior causes excessive fatty acid accumulation [32] and the formation of fatty acid pools [26], thus inducing intracellular lipid accumulation. This phenomenon may explain why *SAMM50* knockdown causes lipid accumulation.

In conclusion, the present study has demonstrated that SNPs (rs738491 and rs2073082) of the *SAMM50* gene are associated with susceptibility to and severity of NAFLD in a sample of Chinese Han population. The underlying mechanism may be attributed to the impaired FAO caused by *SAMM50* deficiency.

## Conflict of interests

The authors declare that they have no conflicts of interest.

## Author’s contributions

XW designed the study. XZ, YW, GS and CX acquired the clinical and laboratory data. ZL, WS and GW analyzed the data and drafted the manuscript.

## Supporting information


**Fig S1.** *SAMM50* knockdown caused lipid accumulation in human hepatoma cells under fatty acid treatment.

**Fig S2.** Decreased fatty acid oxidation in *SAMM50*‐knockdown cells.

**Table S1.** International serial number, gene name and location of SNPs (ABI), as well as the sequence of detection probe.

**Table S2.** The primer pairs used in the present study.

**Table S3.** Comparison of liver function markers among patients with different genotypes at loci rs738491 and rs2073082.

**Table S4.** SNP profiling results of 760 individuals.

## Data Availability

The data that support the findings of this study are available in the supplementary material of this article. Table S4 SNP profiling results of 760 individuals.
