# Supplementary figures and images for: The role of SAMM50 in non‐alcoholic fatty liver disease: from genetics to mechanisms
Source: FEBS Open Bio. 2021 May 27;11(7):1893–906. doi: 10.1002/2211-5463.13146 (PMC8255833; doi:10.1002/2211-5463.13146)

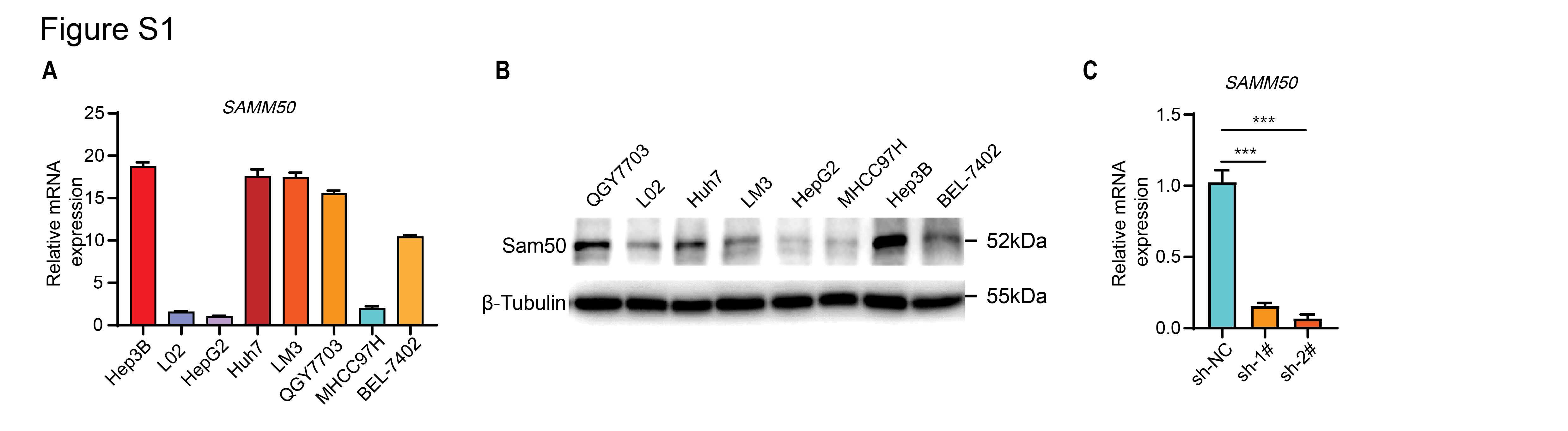

Supplement: Supplementary file 1 — Fig S1. SAMM50 knockdown caused lipid accumulation in human hepatoma cells under fatty acid treatment. [file FEB4-11-1893-s004.tif]

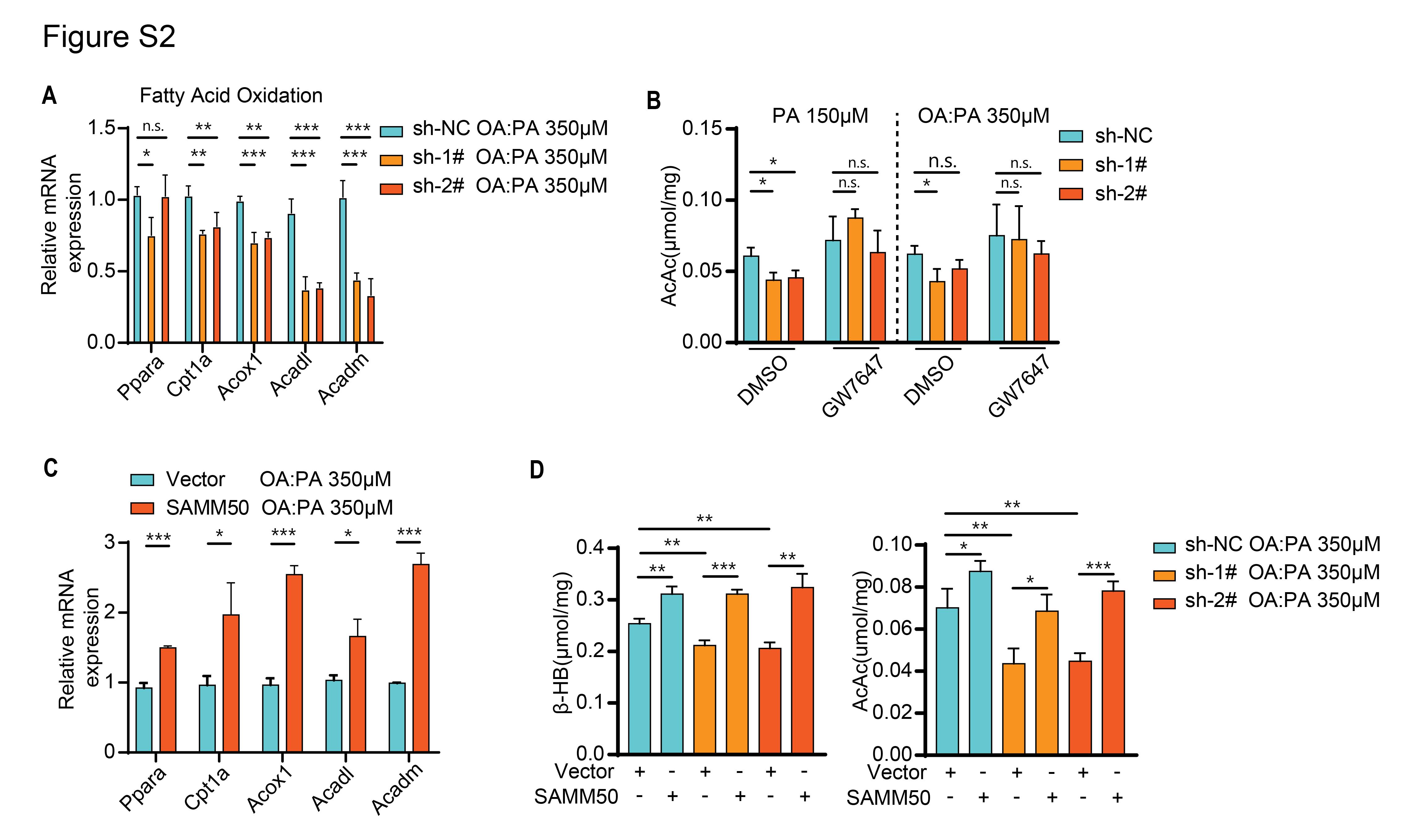

Supplement: Supplementary file 2 — Fig S2. Decreased fatty acid oxidation in SAMM50‐knockdown cells. [file FEB4-11-1893-s006.tif]
