# Supplementary material for: The role of SAMM50 in non‐alcoholic fatty liver disease: from genetics to mechanisms
Source: FEBS Open Bio. 2021 May 27;11(7):1893–906. doi: 10.1002/2211-5463.13146 (PMC8255833; doi:10.1002/2211-5463.13146)
Supplement: Supplementary file 3 — Table S1. International serial number, gene name and location of SNPs (ABI), as well as the sequence of detection probe. [file FEB4-11-1893-s005.docx]

Supplementary Table 1. International serial number, gene name, and location of SNPs (ABI) and the sequence of detection probe.

| SNP | Gene name | Location | Sequence of detection probe |
| --- | --- | --- | --- |
| rs738491 | SAMM50 | Chr.22: 43958231 | GGGCTTCGCCTTTCCGTGCCATTTC[C/T]  TTTGGTGCCATTTACATTTTGGCTT |
| rs2073082 | SAMM50 | Chr.22: 43964127 | GGGTCATGGCCAGCATTCATTCACA[A/G]GCCAGTGCTCAGCATGCATGTTAGG |
