# Supplementary material for: The role of SAMM50 in non‐alcoholic fatty liver disease: from genetics to mechanisms
Source: FEBS Open Bio. 2021 May 27;11(7):1893–906. doi: 10.1002/2211-5463.13146 (PMC8255833; doi:10.1002/2211-5463.13146)
Supplement: Supplementary file 4 — Table S2. The primer pairs used in the present study. [file FEB4-11-1893-s001.docx]

Supplementary Table 2. The primer pairs used in this study

SAMM50-F: AGACGGACAGAGGAATGTCAGC

SAMM50-R: GCAAATGACGCCGTCCTTGAGA

Acacb-F: GACGAGCTGATCTCCATCCTCA

Acacb-R ATGGACTCCACCTGGTTATGCC

Fasn-F: TTCTACGGCTCCACGCTCTTCC

Fasn-R: GAAGAGTCTTCGTCAGCCAGGA

Fads2-F: TGCAACGTGGAGCAGTCCTTCT

Fads2-R: GGCACATAGAGACTTCACCAGC

Srebf1-F: ACTTCTGGAGGCATCGCAAGCA

Srebf1-R: AGGTTCCAGAGGAGGCTACAAG

Elovl6-F: CCATCCAATGGATGCAGGAAAAC

Elovl6-R: CCAGAGCACTAATGGCTTCCTC

Scd-F: CCTGGTTTCACTTGGAGCTGTG

Scd-R: TGTGGTGAAGTTGATGTGCCAGC

Acaca-F: TTCACTCCACCTTGTCAGCGGA

Acaca-R: GTCAGAGAAGCAGCCCATCACT

Dgat2-F: GCTACAGGTCATCTCAGTGCTC

Dgat2-R: GTGAAGTAGAGCACAGCGATGAG

Cd36-F: CAGGTCAACCTATTGGTCAAGCC

Cd36-R: GCCTTCTCATCACCAATGGTCC

Fabp1-F: GGAGGAATGTGAGCTGGAGACA

Fabp1-R: TATGTCGCCGTTGAGTTCGGTC

Fabp2-F: CAATCTAGCAGACGGAACTGAAC

Fabp2-R: CGGACAGTATTCAGTTCGTTTCC

Fabp3-F: GTGGAGTTCGATGAGACAACAGC

Fabp3-R: TGGTCTCTTGCCCGTCCCATTT

Fabp4-F: ACGAGAGGATGATAAACTGGTGG

Fabp4-R: GCGAACTTCAGTCCAGGTCAAC

Fabp5-F: GGTGCATTGGTTCAGCATCAGG

Fabp5-R: TCATAGATCCGAGTACAGGTGAC

Pparα-F: TCGGCGAGGATAGTTCTGGAAG
Pparα-R: GACCACAGGATAAGTCACCGAG

Cpt1a -F: GATCCTGGACAATACCTCGGAG

Cpt1a -R: CTCCACAGCATCAAGAGACTGC

Acox1-F: GGCGCATACATGAAGGAGACCT

Acox1-R: AGGTGAAAGCCTTCAGTCCAGC

Acadl-F: AGGGGATCTGTACTCCGCAG

Acadl-R: CGCAACTACAATCACAACATCAC

Acadm-F: ACAGGGGTTCAGACTGCTATT

Acadm-R:TCCTCCGTTGGTTATCCACAT

Actb-F: CACCATTGGCAATGAGCGGTTC

Actb-R: AGGTCTTTGCGGATGTCCACGT
