# Supplementary material for: The role of SAMM50 in non‐alcoholic fatty liver disease: from genetics to mechanisms
Source: FEBS Open Bio. 2021 May 27;11(7):1893–906. doi: 10.1002/2211-5463.13146 (PMC8255833; doi:10.1002/2211-5463.13146)
Supplement: Supplementary file 5 — Table S3. Comparison of liver function markers among patients with different genotypes at loci rs738491 and rs2073082. [file FEB4-11-1893-s003.docx]

Supplementary Table 3. Comparison of liver function markers among patients with different genotypes at loci rs738491 and rs2073082.

| SNP | ALT(IU/L) | | | AST(IU/L) | | |
| --- | --- | --- | --- | --- | --- | --- |
| genotype | NAFLD | NC | Total | NAFLD | NC | Total |
| rs738491 |  |  |  |  |  |  |
| CC | 21.8±7.7 | 24.2±11.3 | 23.2±10.0 | 24.8±9.1 | 25.2±11.0 | 25.1±10.2 |
| CT+TT | 27.3±12.5* | 22.7±10.3 | 25.1±11.7 | 28.9±12.7* | 24.7±9.7 | 26.9±11.6 |
| rs2073082 |  |  |  |  |  |  |
| AA | 22.9±8.6 | 23.9±10.7 | 23.5±9.8 | 22.4±8.6 | 25.7±10.3 | 24.3±9.7 |
| AG+GG | 26.5±12.0 | 23.0±10.6 | 24.8±11.5 | 28.4±12.3* | 24.9±10.1 | 26.7±11.4 |

Abbreviations: NC: normal controls, ALT: alanine transaminase, AST: aspartate transaminase

Data are presented as mean±SD.

*P*-values was determined by Mann–Whitney U-test.

**P* < 0.05 indicates statistical significance.
