# Supplementary material for: The role of SAMM50 in non‐alcoholic fatty liver disease: from genetics to mechanisms
Source: FEBS Open Bio. 2021 May 27;11(7):1893–906. doi: 10.1002/2211-5463.13146 (PMC8255833; doi:10.1002/2211-5463.13146)
Supplement: Supplementary file 6 — Table S4. SNP profiling results of 760 individuals. [file FEB4-11-1893-s002.docx]

| blood  sample | group  1= NAFLD 2=NC | gender 1=male 2=  female | age | BMI | rs738491  1=CC  2=CT  3=TT | rs2073082  1=AA  2=AG  3=GG | SBP  (mmHg) | DBP  (mmHg) | Severity 1=Mild  2=Mod  to sev | TC  (mmol/L) | TG  (mmol/L) | HDL  (mmol/L) | LDL  (mmol/L) | ALT  (IU/L) | AST  (IU/L) | TBIL  (μmol/L) | DBIL  (μmol/L) | γ-GT  (IU/L) | AKP | GLU (mmol/L) | GA (%) |
| --- | --- | --- | --- | --- | --- | --- | --- | --- | --- | --- | --- | --- | --- | --- | --- | --- | --- | --- | --- | --- | --- |
| 506 | 1 | 2 | 48 | 23.26 | 2 | 2 | 100.0 | 70 | 2 | 5.91 | 1.43 | 1.70 | 1.56 | 24.00 | 16 | 12.7 | 2.9 | 48 | 86 | 6.0 | 15.8 |
| 509 | 1 | 1 | 53 | 24.00 | 1 | 1 | 140.0 | 90 | 1 | 2.32 | .99 | 1.21 | 2.62 | 23.00 | 21 | 16.1 | 3.0 | 31 | 115 | 5.8 | 15.4 |
| 712 | 1 | 2 | 49 | 24.00 | 2 | 2 | 110.0 | 70 | 2 | 2.34 | .83 | 1.03 | 4.15 | 28.00 | 20 | 10.2 | 3.6 | 40 | 109 | 5.3 | 15.4 |
| 715 | 1 | 1 | 35 | 23.31 | 2 | 2 | 120.0 | 80 | 2 | 3.74 | 1.69 | 1.83 | 4.27 | 37.00 | 48 | 14.9 | 4.0 | 47 | 91 | 4.4 | 9.4 |
| 747 | 1 | 2 | 41 | 21.78 | 1 | 2 | 130.0 | 80 | 1 | 6.48 | 2.49 | 1.45 | 2.69 | 28.00 | 32 | 6.3 | 4.3 | 41 | 124 | 5.9 | 14.7 |
| 867 | 1 | 2 | 54 | 23.39 | 1 | 1 | 110.0 | 70 | 1 | 4.30 | .72 | 1.06 | 4.00 | 27.00 | 19 | 9.4 | 2.4 | 77 | 100 | 5.3 | 12.9 |
| 882 | 1 | 2 | 39 | 23.41 | 1 | 1 | 110.0 | 70 | 1 | 4.84 | .82 | 1.02 | 2.02 | 30.00 | 11 | 6.5 | 1.4 | 46 | 62 | 3.9 | 11.6 |
| 897 | 1 | 2 | 49 | 23.34 |  | 2 | 110.0 | 70 | 2 | 3.73 | 1.44 | 1.60 | 4.20 | 39.00 | 18 | 8.7 | 1.5 | 27 | 53 | 4.1 | 12.1 |
| 913 | 1 | 1 | 58 | 22.72 | 1 | 2 | 110.0 | 70 | 2 | 2.47 | 2.81 | 1.73 | 2.04 | 11.00 | 14 | 8.4 | 2.0 | 34 | 125 | 5.2 | 15.1 |
| 914 | 1 | 1 | 58 | 23.12 | 1 | 2 | 110.0 | 70 | 1 | 1.48 | 3.56 | 1.10 | 2.22 | 16.00 | 29 | 9.5 | 1.3 | 12 | 45 | 5.7 | 12.0 |
| 956 | 1 | 1 | 56 | 24.44 | 2 | 2 | 114.0 | 70 | 1 | 2.05 | 1.71 | 1.79 | 1.85 | 31.00 | 12 | 17.1 | 2.2 | 43 | 41 | 5.3 | 12.4 |
| 961 | 1 | 2 | 25 | 25.00 | 2 | 2 | 110.0 | 80 | 1 | 4.04 | .81 | 1.47 | 1.80 | 36.00 | 34 | 12.2 | 1.2 | 18 | 46 | 4.9 | 14.5 |
| 964 | 1 | 1 | 36 | 26.00 | 2 | 2 | 130.0 | 90 | 2 | 2.74 | 1.48 | .99 | 1.53 | 21.00 | 23 | 8.8 | 3.2 | 34 | 122 | 4.0 | 14.6 |
| 975 | 1 | 1 | 34 | 25.00 | 2 | 2 | 100.0 | 70 | 2 | 2.26 | 1.24 | 1.34 | 3.91 | 39.00 | 20 | 3.8 | 2.6 | 11 | 112 | 4.6 | 13.1 |
| 978 | 1 | 1 | 54 | 23.39 | 2 | 2 | 110.0 | 70 | 2 | 3.33 | 2.39 | 1.15 | 4.44 | 35.00 | 15 | 14.0 | 2.8 | 30 | 70 | 4.7 | 15.9 |
| 980 | 1 | 1 | 30 | 22.58 | 2 | 2 | 100.0 | 70 | 1 | 5.92 | 1.20 | 1.30 | 5.25 | 12.00 | 14 | 13.1 | 3.2 | 30 | 104 | 5.9 | 12.0 |
| 983 | 1 | 2 | 43 | 20.82 | 2 | 2 | 110.0 | 80 | 2 | 4.26 | 1.29 | 1.18 | 4.62 | 14.00 | 15 | 15.3 | 1.7 | 51 | 125 | 6.1 | 12.0 |
| 997 | 1 | 2 | 40 | 22.31 | 2 | 2 | 120.0 | 80 | 2 | 6.41 | 2.57 | 1.31 | 4.17 | 37.00 | 21 | 15.4 | 2.1 | 30 | 78 | 5.2 | 11.3 |
| 998 | 1 | 1 | 34 | 23.80 | 2 | 2 | 130.0 | 85 | 2 | 3.48 | 3.13 | 1.17 | 3.54 | 14.00 | 33 | 11.9 | 3.3 | 21 | 102 | 5.0 | 15.3 |
| 1000 | 1 | 1 | 53 | 20.28 | 2 | 2 | 110.0 | 70 | 2 | 1.12 | 1.19 | .97 | 3.61 | 16.00 | 30 | 12.7 | 4.1 | 31 | 59 | 4.9 | 14.8 |
| 1008 | 1 | 1 | 45 | 26.00 | 1 | 1 | 135.0 | 85 | 1 | 4.92 | 2.08 | 1.38 | 2.03 | 12.00 | 12 | 14.5 | 3.7 | 13 | 112 | 4.2 | 13.5 |
| 1217 | 1 | 2 | 57 | 21.71 | 2 | 2 | 110.0 | 70 | 2 | 3.85 | 5.63 | 1.67 | 4.39 | 22.00 | 21 | 17.0 | 3.0 | 23 | 104 | 7.4 | 11.6 |
| 1218 | 1 | 1 | 78 | 22.27 | 2 | 2 | 130.0 | 80 | 2 | 2.93 | .99 | 1.25 | 4.01 | 21.00 | 16 | 7.7 | 2.0 | 13 | 100 | 7.8 | 17.9 |
| 1220 | 1 | 1 | 68 | 21.97 | 2 | 2 | 112.0 | 80 | 2 | 4.84 | 1.84 | 1.30 | 5.45 | 35.00 | 26 | 16.1 | 4.0 | 45 | 115 | 7.4 | 13.5 |
| 1224 | 1 | 2 | 41 | 25.90 | 2 | 2 | 106.0 | 70 | 1 | 4.14 | 1.72 | 1.30 | 4.99 | 18.00 | 21 | 5.8 | 2.6 | 12 | 81 | 5.6 | 13.7 |
| 1613 | 1 | 1 | 60 | 24.68 | 1 | 2 | 120.0 | 80 | 1 | 5.22 | 4.13 | 1.74 | 2.81 | 29.00 | 21 | 12.1 | 3.4 | 26 | 116 | 5.8 | 11.9 |
| 1621 | 1 | 1 | 69 | 23.88 | 2 | 2 | 120.0 | 70 | 1 | 5.88 | 1.78 | 1.19 | 1.91 | 17.00 | 21 | 17.4 | 2.7 | 35 | 119 | 5.9 | 13.2 |
| 1636 | 1 | 1 | 31 | 22.84 | 1 | 2 | 100.0 | 72 | 1 | 2.18 | .95 | 1.15 | 2.62 | 17.00 | 39 | 7.7 | 1.1 | 20 | 51 | 4.4 | 11.2 |
| 1651 | 1 | 1 | 28 | 23.24 | 1 | 1 | 120.0 | 80 | 1 | 1.35 | .71 | 1.65 | 2.47 | 25.00 | 20 | 16.2 | 3.7 | 20 | 77 | 5.8 | 13.4 |
| 1658 | 1 | 2 | 57 | 25.00 | 2 | 2 | 110.0 | 78 | 2 | 5.83 | 2.20 | 1.29 | 3.19 | 26.00 | 17 | 14.1 | 2.5 | 34 | 45 | 4.4 | 15.2 |
| 1664 | 1 | 1 | 48 | 20.45 | 2 | 2 | 112.0 | 80 | 1 | 4.78 | 1.48 | 1.22 | 5.25 | 12.00 | 34 | 6.4 | 2.6 | 38 | 57 | 4.9 | 12.8 |
| 2153 | 1 | 2 | 47 | 22.43 | 1 | 2 | 110.0 | 70 | 1 | 2.41 | .80 | 1.09 | 1.79 | 28.00 | 39 | 14.8 | 3.1 | 14 | 55 | 4.0 | 11.2 |
| 2157 | 1 | 1 | 57 | 22.64 | 2 | 2 | 114.0 | 70 | 2 | 5.97 | 1.89 | 1.69 | 1.59 | 24.00 | 21 | 7.7 | 4.0 | 37 | 102 | 3.9 | 14.1 |
| 2158 | 1 | 1 | 35 | 22.66 | 1 | 1 | 110.0 | 70 | 1 | 5.52 | 1.05 | 1.43 | 3.35 | 41.00 | 25 | 10.8 | 2.9 | 36 | 124 | 4.0 | 13.8 |
| 2161 | 1 | 1 | 53 | 24.00 | 2 | 2 | 120.0 | 80 | 1 | 3.31 | 5.01 | 1.24 | 2.61 | 35.00 | 26 | 7.1 | 3.3 | 79 | 114 | 7.7 | 6.3 |
| 2166 | 1 | 2 | 34 | 22.84 | 2 | 2 | 120.0 | 70 | 2 | 2.81 | 2.23 | 1.71 | 2.19 | 17.00 | 53 | 15.8 | 1.6 | 13 | 73 | 4.9 | 13.9 |
| 2179 | 1 | 1 | 55 | 25.00 | 2 | 2 | 140.0 | 55 | 2 | 2.50 | 1.06 | 1.14 | 1.99 | 14.00 | 18 | 10.7 | 6.1 | 39 | 122 | 4.5 | 14.9 |
| 2189 | 1 | 2 | 52 | 23.89 | 2 | 2 | 110.0 | 80 | 1 | 5.22 | 2.04 | 1.70 | 1.73 | 14.00 | 38 | 11.1 | 1.9 | 25 | 112 | 4.0 | 14.5 |
| 2195 | 1 | 2 | 55 | 19.88 | 2 | 2 | 120.0 | 70 | 1 | 5.91 | 1.72 | 1.29 | 1.70 | 22.00 | 37 | 5.1 | 3.7 | 12 | 87 | 3.9 | 15.2 |
| 2196 | 1 | 2 | 51 | 20.96 |  | 2 | 120.0 | 80 | 2 | 5.37 | 1.73 | 1.13 | 2.39 | 23.00 | 14 | 13.6 | 2.4 | 23 | 74 | 4.3 | 11.3 |
| 2198 | 1 | 2 | 30 | 21.80 | 2 | 2 | 120.0 | 80 | 2 | 5.99 | 1.06 | 1.46 | 4.35 | 43.00 | 48 | 12.4 | 1.3 | 28 | 119 | 5.5 | 13.3 |
| 2204 | 1 | 1 | 58 | 23.89 | 2 | 2 | 140.0 | 80 | 1 | 5.65 | 3.59 | 1.60 | 3.46 | 39.00 | 41 | 9.4 | 3.9 | 75 | 56 | 5.4 | 11.5 |
| 2208 | 1 | 2 | 45 | 22.84 | 2 | 2 | 120.0 | 70 | 2 | 2.23 | 1.19 | 1.20 | 2.57 | 24.00 | 20 | 22.0 | 2.9 | 34 | 99 | 5.7 | 14.7 |
| 2209 | 1 | 2 | 29 | 25.00 |  | 2 | 112.0 | 70 | 1 | 5.93 | 2.89 | 1.34 | 2.68 | 20.00 | 27 | 8.1 | 2.0 | 21 | 121 | 4.2 | 15.3 |
| 2214 | 1 | 2 | 49 | 23.63 | 2 | 2 | 100.0 | 70 | 2 | 5.53 | 2.79 | 1.73 | 2.39 | 31.00 | 29 | 22.0 | 3.5 | 67 | 50 | 5.8 | 12.7 |
| 2216 | 1 | 1 | 47 | 25.00 | 2 | 2 | 140.0 | 100 | 1 | 4.58 | .97 | 1.78 | 2.45 | 15.00 | 26 | 3.8 | 3.0 | 35 | 67 | 5.8 | 12.9 |
| 2220 | 1 | 2 | 57 | 20.07 | 2 | 2 | 130.0 | 80 | 1 | 5.62 | .88 | 1.60 | 2.61 | 40.00 | 17 | 22.0 | 2.9 | 44 | 41 | 5.5 | 13.2 |
| 2224 | 1 | 1 | 62 | 23.15 | 2 | 2 | 140.0 | 90 | 2 | 1.27 | 1.86 | 1.41 | 1.84 | 42.00 | 52 | 19.0 | 2.4 | 66 | 77 | 4.7 | 14.0 |
| 2231 | 1 | 1 | 48 | 24.00 | 1 | 2 | 110.0 | 70 | 1 | 3.45 | .66 | .96 | 1.46 | 11.00 | 29 | 5.2 | 1.9 | 14 | 79 | 5.7 | 11.8 |
| 2233 | 1 | 1 | 55 | 22.96 | 2 | 2 | 120.0 | 70 | 2 | 5.39 | 2.06 | .95 | 3.43 | 20.00 | 21 | 10.7 | 2.2 | 43 | 94 | 7.2 | 14.7 |
| 2236 | 1 | 2 | 49 | 22.41 |  | 2 | 110.0 | 80 | 1 | 4.06 | .74 | 1.49 | 4.06 | 41.00 | 16 | 15.8 | 3.2 | 32 | 81 | 5.2 | 11.2 |
| 2238 | 1 | 2 | 29 | 26.00 | 2 | 2 | 120.0 | 80 | 1 | 6.02 | .88 | 1.75 | 1.80 | 10.00 | 35 | 8.4 | 3.7 | 37 | 95 | 4.4 | 12.5 |
| 2274 | 1 | 1 | 51 | 23.05 | 2 | 2 | 120.0 | 80 | 1 | 6.34 | 1.86 | .99 | 4.46 | 43.00 | 19 | 7.9 | 2.4 | 9 | 100 | 5.9 | 11.9 |
| 2278 | 1 | 1 | 31 | 25.00 | 2 | 2 | 100.0 | 70 | 2 | 5.51 | 2.03 | 1.07 | 3.03 | 28.00 | 40 | 18.0 | 1.1 | 13 | 43 | 4.9 | 15.0 |
| 2284 | 1 | 2 | 32 | 23.31 | 1 | 2 | 110.0 | 70 | 1 | 5.19 | .85 | 1.05 | 1.47 | 21.00 | 33 | 12.7 | 2.1 | 18 | 44 | 5.6 | 14.0 |
| 2285 | 1 | 2 | 50 | 23.38 | 2 | 2 | 120.0 | 80 | 1 | 3.08 | .88 | 1.57 | 2.55 | 28.00 | 32 | 7.4 | 1.9 | 38 | 50 | 4.8 | 15.2 |
| 2287 | 1 | 1 | 59 | 20.81 | 2 | 2 | 110.0 | 70 | 2 | 3.92 | 1.10 | 1.72 | 3.34 | 35.00 | 49 | 10.2 | 2.9 | 7 | 78 | 5.4 | 13.2 |
| 2292 | 1 | 2 | 58 | 22.41 | 1 | 2 | 120.0 | 80 | 2 | 1.00 | .79 | 1.43 | 2.87 | 20.00 | 14 | 7.5 | 4.2 | 11 | 59 | 4.6 | 15.4 |
| 2293 | 1 | 2 | 49 | 23.44 | 2 | 2 | 130.0 | 80 | 2 | 5.41 | 1.88 | 1.29 | 4.99 | 39.00 | 61 | 15.8 | 3.7 | 30 | 89 | 4.7 | 15.6 |
| 2298 | 1 | 2 | 43 | 22.84 | 2 | 2 | 110.0 | 70 | 1 | 3.94 | 1.78 | 1.59 | 3.67 | 18.00 | 16 | 17.0 | 1.2 | 18 | 111 | 5.0 | 12.3 |
| 2307 | 1 | 2 | 50 | 22.79 | 2 | 2 | 87.0 | 76 | 2 | 5.40 | 1.53 | 1.64 | 3.39 | 15.00 | 13 | 12.0 | 1.6 | 42 | 80 | 4.8 | 11.8 |
| 2321 | 1 | 2 | 62 | 20.52 |  | 2 | 120.0 | 80 | 1 | 6.37 | 2.12 | 1.20 | 4.71 | 47.00 | 16 | 16.3 | 1.3 | 19 | 38 | 6.0 | 13.2 |
| 2334 | 1 | 1 | 42 | 24.00 | 1 | 2 | 130.0 | 80 | 1 | 4.21 | 1.33 | 1.80 | 4.00 | 37.00 | 16 | 13.9 | 1.1 | 56 | 106 | 4.5 | 11.5 |
| 2336 | 1 | 2 | 26 | 22.86 | 1 | 1 | 100.0 | 60 | 1 | 2.71 | .64 | 1.76 | 2.17 | 17.00 | 15 | 9.5 | 1.3 | 32 | 123 | 4.4 | 15.9 |
| 2338 | 1 | 2 | 50 | 26.00 | 2 | 2 | 120.0 | 80 | 2 | 3.35 | 1.46 | 1.63 | 2.88 | 21.00 | 18 | 3.7 | 3.3 | 18 | 77 | 4.6 | 12.4 |
| 2344 | 1 | 2 | 47 | 23.71 | 1 | 2 | 105.0 | 70 | 1 | 2.89 | .93 | 1.10 | 2.89 | 19.00 | 30 | 4.0 | 2.9 | 14 | 89 | 5.2 | 11.8 |
| 2348 | 1 | 2 | 50 | 23.99 | 2 | 2 | 110.0 | 80 | 1 | 4.35 | 1.87 | 1.68 | 2.10 | 12.00 | 41 | 15.5 | 3.7 | 34 | 104 | 4.2 | 14.6 |
| 2351 | 1 | 1 | 46 | 22.49 | 1 | 2 | 88.0 | 78 | 1 | 5.21 | 1.80 | 1.48 | 3.36 | 15.00 | 40 | 5.2 | 3.2 | 45 | 103 | 5.8 | 15.1 |
| 2356 | 1 | 2 | 36 | 23.32 | 2 | 2 | 105.0 | 70 | 2 | 5.66 | 1.67 | 1.64 | 1.85 | 25.00 | 33 | 16.7 | 1.3 | 16 | 108 | 5.3 | 11.6 |
| 2359 | 1 | 1 | 49 | 23.34 | 2 | 2 | 112.0 | 80 | 1 | 5.00 | 2.09 | 1.09 | 3.69 | 27.00 | 24 | 6.3 | 3.4 | 27 | 45 | 5.9 | 16.2 |
| 2360 | 1 | 2 | 49 | 22.38 | 2 | 2 | 120.0 | 80 | 1 | 5.88 | 1.38 | 1.21 | 2.12 | 15.00 | 40 | 6.6 | 2.3 | 37 | 99 | 4.8 | 13.3 |
| 2362 | 1 | 1 | 35 | 19.94 | 2 | 2 | 114.0 | 70 | 2 | 3.84 | .71 | 1.50 | 1.88 | 16.00 | 14 | 5.2 | 2.4 | 25 | 92 | 4.6 | 12.8 |
| 2364 | 1 | 2 | 48 | 23.01 | 2 | 2 | 110.0 | 60 | 2 | 2.20 | 1.17 | 1.82 | 1.61 | 11.00 | 30 | 8.7 | 2.4 | 12 | 102 | 4.8 | 15.9 |
| 2473 | 1 | 2 | 22 | 19.84 | 2 | 2 | 120.0 | 80 | 1 | 2.51 | 1.63 | 1.35 | 3.23 | 30.00 | 11 | 15.5 | 1.7 | 55 | 125 | 5.6 | 12.4 |
| 2474 | 1 | 2 | 47 | 24.00 | 2 | 2 | 100.0 | 70 | 1 | 4.09 | 1.49 | 1.69 | 1.40 | 20.00 | 19 | 8.6 | 3.3 | 31 | 134 | 4.2 | 13.1 |
| 2476 | 1 | 1 | 49 | 22.59 | 2 | 2 | 114.0 | 70 | 2 | 4.00 | 1.21 | 1.01 | 3.95 | 36.00 | 17 | 7.2 | 1.9 | 13 | 112 | 5.2 | 15.2 |
| 2484 | 1 | 1 | 31 | 23.62 | 2 | 2 | 110.0 | 70 | 1 | 2.61 | .81 | 1.36 | 3.73 | 10.00 | 44 | 14.1 | 1.1 | 28 | 59 | 4.0 | 14.4 |
| 2487 | 1 | 2 | 45 | 21.83 | 1 | 2 | 110.0 | 70 | 2 | 4.81 | 5.16 | 1.34 | 2.73 | 28.00 | 26 | 15.7 | 1.0 | ## | 117 | 4.7 | 11.9 |
| 2497 | 1 | 2 | 49 | 20.98 | 2 | 2 | 105.0 | 60 | 2 | 5.24 | 1.58 | 1.04 | 2.90 | 37.00 | 18 | 11.9 | 4.0 | 43 | 63 | 5.0 | 15.0 |
| 2502 | 1 | 2 | 32 | 22.48 | 1 | 2 | 114.0 | 70 | 1 | 3.31 | 2.02 | 1.62 | 4.12 | 17.00 | 34 | 5.2 | 4.1 | 48 | 72 | 4.8 | 13.2 |
| 2535 | 1 | 2 | 64 | 22.68 | 2 | 2 | 120.0 | 70 | 2 | 1.16 | 1.71 | 1.63 | 3.20 | 23.00 | 20 | 4.5 | 3.5 | 38 | 44 | 4.0 | 12.6 |
| 2541 | 1 | 1 | 57 | 25.47 | 2 | 2 | 120.0 | 80 | 2 | 4.24 | 1.44 | 1.08 | 3.05 | 38.00 | 21 | 8.3 | 3.3 | 11 | 106 | 4.4 | 14.2 |
| 2544 | 1 | 2 | 28 | 24.00 | 2 | 2 | 100.0 | 70 | 1 | 3.29 | .87 | 1.29 | 2.32 | 35.00 | 22 | 12.2 | 3.7 | 37 | 125 | 5.9 | 12.4 |
| 2549 | 1 | 1 | 44 | 24.00 | 1 | 1 | 130.0 | 80 | 2 | 1.23 | 3.42 | 1.34 | 3.66 | 44.00 | 19 | 12.4 | 1.2 | 12 | 105 | 7.0 | 12.1 |
| 2559 | 1 | 2 | 54 | 24.00 | 2 | 2 | 130.0 | 80 | 2 | 5.58 | 2.53 | 1.76 | 3.11 | 33.00 | 36 | 13.2 | 3.2 | 47 | 90 | 5.0 | 11.9 |
| 2569 | 1 | 1 | 54 | 25.00 | 2 | 2 | 140.0 | 90 | 2 | 3.20 | 1.84 | 1.57 | 2.22 | 21.00 | 13 | 3.6 | 2.8 | ## | 93 | 7.6 | 14.6 |
| 2572 | 1 | 1 | 23 | 33.00 | 2 | 2 | 120.0 | 70 | 1 | 2.59 | .63 | 1.15 | 3.78 | 22.00 | 38 | 9.3 | 1.7 | 34 | 53 | 6.0 | 13.0 |
| 2579 | 1 | 1 | 39 | 21.99 | 2 | 2 | 112.0 | 60 | 2 | 1.72 | .87 | 1.50 | 4.10 | 36.00 | 24 | 7.2 | 4.4 | 54 | 99 | 3.9 | 12.6 |
| 2924 | 1 | 2 | 47 | 23.71 | 2 | 2 | 112.0 | 70 | 1 | 1.68 | 1.14 | 1.19 | 3.58 | 34.00 | 30 | 16.3 | 2.8 | 11 | 61 | 4.9 | 14.0 |
| 2925 | 1 | 1 | 63 | 22.76 | 2 | 2 | 120.0 | 80 | 2 | 6.96 | 2.12 | 1.18 | 5.08 | 43.00 | 37 | 5.1 | 1.4 | 37 | 67 | 5.8 | 15.3 |
| 2926 | 1 | 1 | 24 | 25.00 | 1 | 1 | 120.0 | 80 | 1 | 2.94 | .69 | 1.35 | 2.15 | 23.00 | 30 | 13.7 | 2.4 | 30 | 58 | 5.0 | 12.9 |
| 2930 | 1 | 2 | 63 | 20.43 | 2 | 2 | 130.0 | 80 | 1 | 2.99 | 2.38 | 1.18 | 3.92 | 16.00 | 29 | 8.0 | 3.0 | 19 | 116 | 5.7 | 13.8 |
| 2931 | 1 | 2 | 48 | 23.05 | 2 | 2 | 120.0 | 80 | 2 | 5.62 | 2.31 | 1.77 | 1.64 | 12.00 | 55 | 6.6 | 2.3 | 12 | 104 | 6.6 | 13.2 |
| 2932 | 1 | 2 | 50 | 22.86 | 1 | 2 | 120.0 | 80 | 1 | 5.82 | 1.21 | 1.21 | 3.75 | 17.00 | 39 | 9.9 | 3.0 | 36 | 40 | 5.8 | 11.1 |
| 2934 | 1 | 2 | 37 | 23.88 | 2 | 2 | 114.0 | 70 | 1 | 3.35 | 1.39 | 1.73 | 4.10 | 27.00 | 33 | 14.1 | 1.6 | 12 | 107 | 5.7 | 15.7 |
| 2935 | 1 | 1 | 65 | 24.00 | 2 | 2 | 140.0 | 111 | 2 | 6.22 | 1.15 | 1.13 | 4.68 | 45.00 | 31 | 10.1 | 3.6 | 24 | 86 | 5.5 | 11.9 |
| 2937 | 1 | 2 | 54 | 21.22 | 2 | 2 | 100.0 | 60 | 1 | 1.65 | .80 | 1.73 | 2.41 | 35.00 | 38 | 3.7 | 1.4 | 27 | 106 | 5.0 | 14.1 |
| 2939 | 1 | 1 | 62 | 20.31 | 2 | 2 | 120.0 | 80 | 2 | 1.04 | .95 | 1.21 | 1.58 | 18.00 | 30 | 16.8 | 1.5 | 16 | 53 | 5.4 | 15.1 |
| 2941 | 1 | 2 | 43 | 23.88 | 2 | 2 | 110.0 | 70 | 2 | 2.55 | 1.64 | 1.26 | 2.95 | 25.00 | 23 | 12.7 | 1.1 | 16 | 126 | 4.2 | 14.4 |
| 2942 | 1 | 1 | 52 | 21.79 | 2 | 2 | 100.0 | 70 | 2 | 5.59 | 2.47 | 1.14 | 2.54 | 20.00 | 49 | 11.4 | 2.0 | 21 | 70 | 5.2 | 15.7 |
| 2943 | 1 | 2 | 54 | 21.79 | 2 | 2 | 120.0 | 80 | 2 | 6.70 | 2.01 | 1.44 | 4.98 | 43.00 | 14 | 10.9 | 1.4 | 12 | 54 | 5.3 | 15.6 |
| 2944 | 1 | 1 | 60 | 23.12 | 2 | 2 | 130.0 | 80 | 2 | 4.26 | 1.45 | 1.82 | 2.77 | 16.00 | 40 | 12.9 | 4.3 | 99 | 71 | 5.9 | 13.2 |
| 2948 | 1 | 2 | 65 | 25.00 | 1 | 2 | 120.0 | 80 | 2 | 5.82 | 1.21 | 1.44 | 2.24 | 13.00 | 40 | 13.1 | 4.2 | 23 | 40 | 8.1 | 21.4 |
| 2950 | 1 | 1 | 53 | 18.00 | 1 | 1 | 120.0 | 70 | 2 | 5.84 | .60 | 1.33 | 2.31 | 11.00 | 32 | 4.0 | 3.8 | 47 | 106 | 5.3 | 14.6 |
| 2953 | 1 | 2 | 47 | 22.43 | 1 | 1 | 120.0 | 80 | 1 | 4.12 | 1.10 | 1.67 | 2.09 | 15.00 | 21 | 6.9 | 3.4 | 14 | 125 | 4.4 | 11.6 |
| 2954 | 1 | 1 | 57 | 23.37 | 2 | 2 | 120.0 | 80 | 2 | 1.55 | 1.13 | 1.20 | 3.41 | 13.00 | 39 | 10.3 | 3.1 | 42 | 67 | 4.5 | 17.9 |
| 2958 | 1 | 1 | 77 | 23.78 | 2 | 2 | 105.0 | 70 | 2 | 3.93 | 1.71 | 1.57 | 2.62 | 30.00 | 26 | 7.5 | 3.9 | 10 | 55 | 5.5 | 11.8 |
| 2959 | 1 | 2 | 55 | 23.15 | 2 | 2 | 100.0 | 70 | 2 | 5.64 | 2.93 | 1.57 | 2.68 | 15.00 | 38 | 13.5 | 3.0 | 45 | 114 | 4.5 | 12.8 |
| 2960 | 1 | 2 | 54 | 21.10 | 1 | 2 | 110.0 | 70 | 2 | 1.09 | .89 | 1.30 | 1.89 | 22.00 | 36 | 9.0 | 1.2 | 20 | 42 | 4.1 | 13.2 |
| 2964 | 1 | 1 | 56 | 24.03 | 2 | 2 | 130.0 | 80 | 2 | 6.09 | 2.65 | 1.70 | 3.99 | 14.00 | 38 | 11.8 | 1.5 | ## | 81 | 4.2 | 15.2 |
| 2965 | 1 | 1 | 53 | 22.68 | 1 | 1 | 140.0 | 90 | 1 | 1.81 | 1.17 | .97 | 3.83 | 13.00 | 21 | 3.9 | 4.2 | 16 | 82 | 3.9 | 14.0 |
| 2969 | 1 | 1 | 36 | 23.83 | 2 | 2 | 110.0 | 70 | 2 | 1.29 | 3.06 | 1.14 | 1.47 | 39.00 | 11 | 9.6 | 1.8 | 10 | 117 | 4.9 | 13.4 |
| 2971 | 1 | 1 | 74 | 23.53 | 2 | 2 | 120.0 | 80 | 2 | 1.90 | .82 | 1.45 | 1.96 | 51.00 | 34 | 12.5 | 1.3 | 62 | 105 | 5.3 | 12.3 |
| 2976 | 1 | 1 | 60 | 23.67 | 2 | 2 | 110.0 | 70 | 2 | 5.69 | 1.75 | 1.50 | 3.67 | 13.00 | 22 | 7.8 | 3.3 | 46 | 99 | 4.8 | 13.3 |
| 2977 | 1 | 2 | 53 | 25.00 | 1 | 2 | 100.0 | 70 | 1 | 5.08 | .83 | 1.50 | 1.44 | 12.00 | 21 | 6.1 | 2.5 | 35 | 124 | 6.1 | 13.2 |
| 2981 | 1 | 2 | 33 | 24.00 | 2 | 2 | 110.0 | 80 | 1 | 1.01 | 3.44 | 1.80 | 2.53 | 13.00 | 32 | 8.9 | 3.8 | 7 | 94 | 5.7 | 15.0 |
| 2982 | 1 | 2 | 36 | 19.05 | 2 | 2 | 100.0 | 70 | 2 | 3.99 | .82 | 1.12 | 4.14 | 26.00 | 40 | 9.8 | 3.3 | 17 | 120 | 4.4 | 15.6 |
| 2983 | 1 | 2 | 23 | 23.01 | 1 | 2 | 100.0 | 70 | 1 | 1.98 | .61 | 1.48 | 2.48 | 22.00 | 34 | 15.3 | 2.5 | 48 | 115 | 5.0 | 16.0 |
| 2992 | 1 | 2 | 51 | 24.00 | 2 | 2 | 120.0 | 70 | 2 | 4.82 | 1.94 | 1.42 | 3.31 | 21.00 | 40 | 6.2 | 3.3 | 16 | 83 | 4.6 | 11.6 |
| 2993 | 1 | 1 | 46 | 22.89 | 2 | 2 | 112.0 | 70 | 2 | 2.52 | 1.15 | 1.49 | 3.83 | 38.00 | 23 | 3.9 | 4.2 | 28 | 93 | 4.9 | 13.5 |
| 2995 | 1 | 1 | 56 | 26.00 | 2 | 2 | 120.0 | 70 | 2 | 1.69 | 1.10 | 1.20 | 4.06 | 14.00 | 36 | 16.0 | 4.2 | 22 | 126 | 5.4 | 19.2 |
| 3000 | 1 | 1 | 45 | 23.94 | 2 | 2 | 114.0 | 70 | 2 | 5.40 | 1.73 | 1.38 | 2.65 | 21.00 | 39 | 5.5 | 4.1 | 54 | 39 | 4.1 | 14.0 |
| 3003 | 1 | 2 | 25 | 22.86 |  | 2 | 150.0 | 114 | 2 | 5.65 | 1.77 | 1.51 | 2.08 | 24.00 | 36 | 14.0 | 3.6 | 17 | 51 | 4.1 | 14.7 |
| 3005 | 1 | 1 | 23 | 21.93 | 1 | 1 | 130.0 | 80 | 2 | 5.66 | .90 | 1.10 | 3.64 | 24.00 | 20 | 16.1 | 4.2 | 25 | 38 | 4.1 | 13.5 |
| 3006 | 1 | 1 | 49 | 24.00 | 2 | 2 | 110.0 | 70 | 1 | 5.57 | 1.48 | 1.82 | 3.26 | 39.00 | 14 | 5.7 | 4.2 | 34 | 39 | 7.1 | 17.4 |
| 3008 | 1 | 1 | 54 | 24.00 | 2 | 2 | 114.0 | 70 | 1 | 2.75 | 1.47 | .72 | 4.03 | 50.00 | 19 | 8.5 | 1.9 | 42 | 113 | 4.8 | 16.4 |
| 3013 | 1 | 1 | 24 | 22.50 | 2 | 2 | 120.0 | 80 | 2 | 2.73 | 2.28 | 1.59 | 2.55 | 40.00 | 23 | 3.5 | 2.5 | 37 | 103 | 5.7 | 15.6 |
| 3017 | 1 | 2 | 49 | 22.84 | 2 | 2 | 100.0 | 60 | 1 | 3.90 | 1.38 | 1.28 | 1.46 | 26.00 | 19 | 10.9 | 2.0 | 18 | 109 | 5.0 | 12.5 |
| 3031 | 1 | 1 | 32 | 19.38 | 2 | 2 | 110.0 | 70 | 2 | 2.39 | 1.23 | 1.57 | 2.61 | 13.00 | 32 | 19.0 | 2.6 | 29 | 103 | 4.0 | 11.8 |
| 3042 | 1 | 2 | 52 | 22.32 | 2 | 2 | 120.0 | 70 | 1 | 7.01 | 2.95 | .91 | 4.97 | 42.00 | 35 | 8.1 | 2.4 | ## | 51 | 5.4 | 13.8 |
| 3048 | 1 | 1 | 47 | 22.95 | 2 | 2 | 130.0 | 70 | 1 | 4.29 | 1.50 | 1.74 | 2.90 | 25.00 | 41 | 8.1 | 2.7 | 49 | 44 | 7.3 | 21.9 |
| 3049 | 1 | 1 | 46 | 23.44 | 2 | 2 | 120.0 | 80 | 2 | 3.03 | 1.48 | 1.49 | 2.30 | 30.00 | 17 | 14.9 | 3.3 | 17 | 113 | 5.7 | 13.2 |
| 3052 | 1 | 1 | 45 | 22.21 | 2 | 2 | 130.0 | 80 | 2 | 5.23 | 2.56 | 1.50 | 3.41 | 28.00 | 16 | 11.4 | 1.2 | 29 | 75 | 5.4 | 12.9 |
| 3053 | 1 | 1 | 57 | 19.61 | 2 | 2 | 120.0 | 70 | 2 | 3.59 | .70 | 1.52 | 4.12 | 27.00 | 31 | 6.4 | 1.8 | 33 | 110 | 5.0 | 16.0 |
| 3596 | 1 | 1 | 60 | 19.81 | 2 | 2 | 130.0 | 70 | 1 | 2.95 | 1.64 | .90 | 3.39 | 24.00 | 21 | 19.0 | 2.7 | 49 | 119 | 6.1 | 14.2 |
| 3620 | 1 | 1 | 77 | 25.00 | 1 | 1 | 160.0 | 70 | 2 | 3.38 | 1.10 | 1.36 | 3.90 | 23.00 | 18 | 5.9 | 2.4 | 61 | 95 | 5.2 | 14.6 |
| 3626 | 1 | 1 | 51 | 24.00 | 2 | 2 | 110.0 | 80 | 2 | 6.52 | 3.31 | .97 | 4.28 | 47.00 | 32 | 10.3 | 1.4 | 77 | 114 | 5.3 | 12.1 |
| 3628 | 1 | 1 | 64 | 25.00 | 2 | 2 | 140.0 | 85 | 2 | 4.32 | 1.10 | 1.20 | 2.52 | 39.00 | 24 | 15.9 | 2.7 | 27 | 43 | 4.2 | 14.9 |
| 3630 | 1 | 1 | 81 | 23.39 | 2 | 2 | 110.0 | 70 | 2 | 2.48 | 1.30 | 1.02 | 4.07 | 34.00 | 27 | 27.0 | ### | 38 | 48 | 4.7 | 14.9 |
| 3632 | 1 | 1 | 58 | 25.00 | 1 | 2 | 150.0 | 98 | 2 | 1.86 | 1.24 | 1.50 | 2.54 | 17.00 | 19 | 4.0 | 1.6 | 19 | 103 | 4.5 | 12.4 |
| 3637 | 1 | 2 | 34 | 23.66 | 2 | 2 | 105.0 | 70 | 1 | 4.77 | 1.66 | 1.10 | 2.91 | 22.00 | 30 | 15.0 | 3.8 | 11 | 41 | 5.8 | 14.9 |
| 3638 | 1 | 1 | 53 | 23.59 | 2 | 2 | 150.0 | 90 | 2 | 1.36 | 1.81 | 1.22 | 2.72 | 27.00 | 40 | 10.2 | 4.3 | 44 | 71 | 5.9 | 15.9 |
| 3643 | 1 | 1 | 84 | 24.02 | 1 | 2 | 140.0 | 80 | 1 | 6.40 | 1.42 | 1.15 | 4.20 | 21.00 | 36 | 15.3 | 4.2 | 24 | 63 | 5.3 | 11.5 |
| 3644 | 1 | 2 | 37 | 23.63 | 1 | 2 | 90.0 | 60 | 1 | 2.75 | 1.41 | 1.57 | 2.10 | 25.00 | 33 | 15.7 | 2.8 | 26 | 105 | 6.0 | 15.3 |
| 3649 | 1 | 1 | 30 | 23.18 | 2 | 2 | 110.0 | 80 | 1 | 4.42 | 1.60 | 1.81 | 5.12 | 8.00 | 58 | 14.8 | 2.2 | 18 | 98 | 6.8 | 18.0 |
| 3656 | 1 | 1 | 56 | 23.92 | 1 | 2 | 116.0 | 70 | 1 | 1.29 | .85 | 1.61 | 2.67 | 21.00 | 15 | 8.9 | 3.1 | 22 | 115 | 5.2 | 15.1 |
| 3657 | 1 | 1 | 58 | 25.00 | 2 | 2 | 106.0 | 70 | 1 | 6.58 | 2.99 | .91 | 2.68 | 37.00 | 16 | 8.5 | 2.9 | 67 | 94 | 5.4 | 15.2 |
| 3728 | 1 | 2 | 55 | 20.96 | 2 | 2 | 110.0 | 70 | 2 | 5.75 | 2.47 | 1.24 | 2.46 | 18.00 | 34 | 14.5 | 1.1 | 75 | 39 | 5.4 | 15.8 |
| 3737 | 1 | 2 | 59 | 21.45 | 2 | 2 | 150.0 | 70 | 1 | 1.59 | 1.29 | 1.15 | 3.71 | 17.00 | 24 | 15.5 | 3.1 | 40 | 41 | 4.3 | 11.7 |
| 3739 | 1 | 1 | 27 | 22.76 | 2 | 2 | 120.0 | 85 | 2 | 4.45 | 1.22 | 1.47 | 2.13 | 36.00 | 31 | 5.8 | 4.0 | 41 | 84 | 4.2 | 13.9 |
| 3740 | 1 | 1 | 53 | 25.00 |  | 2 | 130.0 | 90 | 1 | 3.34 | 1.88 | 1.83 | 2.30 | 11.00 | 30 | 15.5 | 4.0 | 25 | 108 | 4.4 | 15.8 |
| 3743 | 1 | 2 | 40 | 25.00 | 2 | 2 | 100.0 | 70 | 1 | 6.00 | 1.77 | 1.10 | 2.78 | 11.00 | 14 | 10.6 | 4.0 | 28 | 66 | 5.6 | 13.5 |
| 3744 | 1 | 2 | 39 | 22.22 | 2 | 2 | 110.0 | 70 | 1 | 6.77 | .86 | 1.78 | 5.03 | 42.00 | 31 | 10.3 | 3.2 | 38 | 75 | 5.6 | 11.6 |
| 4005 | 1 | 2 | 43 | 22.95 | 2 | 2 | 90.0 | 60 | 2 | 6.06 | 3.16 | 1.12 | 4.28 | 46.00 | 57 | 18.1 | 3.4 | 42 | 104 | 4.2 | 13.1 |
| 4021 | 1 | 2 | 40 | 26.00 | 2 | 2 | 100.0 | 70 | 1 | 3.28 | .89 | 1.71 | 3.34 | 17.00 | 35 | 14.6 | 2.6 | 21 | 94 | 5.3 | 15.7 |
| 4027 | 1 | 1 | 51 | 25.00 | 2 | 2 | 120.0 | 84 | 2 | 3.00 | .81 | 1.37 | 3.96 | 6.00 | 20 | 6.1 | 3.4 | 60 | 123 | 5.0 | 13.0 |
| 4033 | 1 | 1 | 54 | 22.49 | 2 | 2 | 120.0 | 84 | 2 | 5.26 | 4.01 | 1.13 | 3.46 | 32.00 | 37 | 10.5 | 2.3 | 43 | 176 | 5.8 | 13.0 |
| 4035 | 1 | 2 | 30 | 22.86 | 2 | 2 | 120.0 | 70 | 2 | 6.21 | 2.06 | 1.19 | 4.52 | 45.00 | 17 | 13.2 | 3.3 | 90 | 87 | 6.5 | 17.0 |
| 4038 | 1 | 2 | 54 | 25.00 | 2 | 2 | 120.0 | 80 | 2 | 1.67 | 3.70 | 1.84 | 2.05 | 16.00 | 12 | 14.8 | 1.8 | 43 | 49 | 5.2 | 12.2 |
| 4039 | 1 | 2 | 58 | 24.00 | 2 | 2 | 106.0 | 70 | 1 | 6.36 | 1.63 | 1.09 | 4.48 | 42.00 | 47 | 12.3 | 3.6 | 21 | 57 | 5.6 | 15.3 |
| 4040 | 1 | 2 | 55 | 24.44 | 1 | 2 | 100.0 | 66 | 1 | 5.95 | 1.06 | 1.70 | 3.09 | 25.00 | 18 | 12.9 | 3.7 | 30 | 113 | 4.4 | 12.8 |
| 4044 | 1 | 2 | 57 | 25.00 | 2 | 2 | 120.0 | 78 | 2 | 7.84 | 3.55 | 1.48 | 5.57 | 44.00 | 17 | 16.4 | 2.6 | 28 | 57 | 4.8 | 12.0 |
| 4047 | 1 | 2 | 48 | 20.55 | 2 | 2 | 110.0 | 80 | 1 | 4.13 | 1.68 | 1.21 | 2.58 | 12.00 | 38 | 10.1 | 4.2 | 14 | 118 | 4.5 | 15.5 |
| 4071 | 1 | 2 | 29 | 23.42 | 2 | 2 | 120.0 | 80 | 2 | 5.05 | 2.26 | 1.05 | 3.19 | 26.00 | 32 | 13.4 | 4.1 | 28 | 49 | 5.2 | 13.8 |
| 4078 | 1 | 1 | 55 | 22.05 | 2 | 2 | 120.0 | 80 | 2 | 6.09 | 1.27 | 1.11 | 4.66 | 43.00 | 23 | 27.5 | 7.3 | 48 | 90 | 5.9 | 12.2 |
| 4079 | 1 | 1 | 53 | 22.04 | 2 | 2 | 100.0 | 64 | 1 | 2.60 | 1.50 | 1.08 | 4.05 | 38.00 | 57 | 12.2 | 3.5 | 50 | 86 | 6.0 | 12.1 |
| 4085 | 1 | 2 | 56 | 24.00 | 2 | 2 | 120.0 | 80 | 2 | 5.31 | 1.92 | 1.84 | 3.31 | 33.00 | 20 | 15.9 | 1.6 | 29 | 88 | 4.1 | 12.8 |
| 4087 | 1 | 2 | 47 | 23.71 | 2 | 2 | 120.0 | 70 | 1 | 5.54 | .98 | 1.68 | 2.20 | 37.00 | 36 | 5.5 | 2.0 | 34 | 104 | 4.6 | 11.1 |
| 4088 | 1 | 2 | 56 | 22.72 | 2 | 2 | 100.0 | 74 | 2 | 5.00 | 3.05 | 1.02 | 1.31 | 30.00 | 14 | 16.0 | 4.2 | 17 | 117 | 5.9 | 11.0 |
| 4094 | 1 | 1 | 55 | 23.12 | 1 | 2 | 114.0 | 80 | 1 | 6.61 | 2.51 | 1.24 | 4.97 | 11.00 | 14 | 14.2 | 3.8 | 77 | 121 | 4.7 | 15.6 |
| 4105 | 1 | 1 | 31 | 24.01 | 2 |  | 100.0 | 70 | 1 | 2.56 | 1.45 | 1.03 | 3.51 | 13.00 | 20 | 6.0 | 3.1 | 55 | 114 | 4.0 | 15.5 |
| 4111 | 1 | 2 | 59 | 22.04 | 2 | 2 | 140.0 | 90 | 1 | 5.75 | .69 | 1.56 | 2.54 | 35.00 | 13 | 16.8 | 1.8 | 50 | 71 | 4.0 | 15.0 |
| 4112 | 1 | 2 | 55 | 23.23 | 1 | 1 | 114.0 | 70 | 2 | 6.95 | 2.76 | 1.69 | 5.16 | 20.00 | 14 | 4.0 | 4.2 | 18 | 81 | 5.9 | 14.3 |
| 4117 | 1 | 1 | 34 | 23.38 | 1 | 1 | 112.0 | 80 | 1 | 4.32 | .68 | 1.75 | 4.06 | 11.00 | 33 | 12.9 | 2.7 | 44 | 89 | 4.4 | 13.0 |
| 4118 | 1 | 2 | 32 | 22.49 | 2 | 2 | 130.0 | 90 | 2 | 3.44 | .63 | 1.77 | 3.87 | 15.00 | 40 | 10.9 | 1.6 | 46 | 61 | 5.2 | 13.4 |
| 4127 | 1 | 2 | 36 | 24.00 | 2 | 2 | 100.0 | 60 | 1 | 3.67 | 1.24 | .99 | 3.75 | 15.00 | 32 | 7.7 | 1.3 | 38 | 96 | 4.8 | 15.6 |
| 4130 | 1 | 1 | 28 | 24.07 | 1 | 2 | 100.0 | 66 | 2 | 1.65 | .71 | 1.27 | 1.72 | 17.00 | 19 | 6.6 | 1.0 | 21 | 102 | 4.8 | 13.1 |
| 4146 | 1 | 2 | 52 | 22.65 | 1 | 2 | 112.0 | 70 | 1 | 5.66 | 1.15 | 1.72 | 1.96 | 14.00 | 16 | 4.7 | 4.1 | 26 | 75 | 4.5 | 13.2 |
| 4157 | 1 | 2 | 46 | 31.00 | 2 | 2 | 110.0 | 70 | 1 | 4.25 | 1.01 | 1.53 | 3.77 | 26.00 | 32 | 15.1 | 4.4 | 28 | 83 | 4.6 | 12.8 |
| 4162 | 1 | 1 | 54 | 24.00 | 2 | 2 | 130.0 | 90 | 1 | 3.40 | 1.52 | 1.26 | 2.78 | 31.00 | 35 | 3.6 | 3.0 | 45 | 95 | 5.9 | 11.7 |
| 4178 | 1 | 2 | 52 | 22.77 | 2 | 2 | 130.0 | 94 | 2 | 5.67 | 4.56 | .65 | 2.87 | 38.00 | 20 | 10.8 | 1.8 | 49 | 111 | 5.0 | 11.1 |
| 4184 | 1 | 1 | 60 | 25.00 | 2 | 2 | 130.0 | 95 | 2 | 4.01 | 1.09 | 1.33 | 1.37 | 26.00 | 15 | 19.1 | 7.5 | 11 | 82 | 4.8 | 14.9 |
| 4189 | 1 | 1 | 33 | 25.00 | 2 | 2 | 100.0 | 65 | 1 | 4.43 | 1.03 | 1.17 | 1.76 | 30.00 | 13 | 8.9 | 3.0 | 36 | 82 | 4.2 | 14.4 |
| 4190 | 1 | 1 | 37 | 22.86 | 1 | 1 | 120.0 | 86 | 2 | 2.42 | .68 | 1.02 | 1.55 | 23.00 | 18 | 3.5 | 3.4 | 30 | 114 | 5.9 | 12.4 |
| 4394 | 1 | 1 | 31 | 20.52 | 2 | 2 | 112.0 | 70 | 2 | 2.34 | 1.45 | .94 | 4.14 | 36.00 | 26 | 4.7 | 1.2 | 15 | 63 | 5.9 | 13.8 |
| 4400 | 1 | 2 | 58 | 25.00 | 2 | 2 | 100.0 | 70 | 1 | 5.44 | 3.65 | 1.34 | 3.47 | 28.00 | 21 | 9.5 | 3.7 | 16 | 98 | 4.2 | 13.7 |
| 4405 | 1 | 1 | 30 | 25.00 | 2 | 2 | 120.0 | 80 | 2 | 5.56 | 2.38 | 1.16 | 3.15 | 26.00 | 40 | 5.1 | 4.1 | 27 | 126 | 5.2 | 11.5 |
| 4416 | 1 | 1 | 27 | 24.00 | 2 | 2 | 118.0 | 70 | 1 | 5.45 | .94 | 1.84 | 3.64 | 10.00 | 33 | 20.9 | 7.5 | 34 | 91 | 4.8 | 14.4 |
| 4443 | 1 | 1 | 30 | 22.60 | 2 | 2 | 100.0 | 60 | 1 | 3.78 | 2.45 | 1.26 | 2.28 | 40.00 | 16 | 4.7 | 3.7 | ## | 45 | 6.0 | 12.6 |
| 4450 | 1 | 1 | 24 | 22.86 | 2 | 2 | 100.0 | 72 | 1 | 1.21 | .67 | 1.59 | 4.08 | 38.00 | 22 | 15.7 | 2.4 | 19 | 47 | 5.2 | 16.0 |
| 4452 | 1 | 2 | 27 | 25.00 | 2 | 2 | 140.0 | 80 | 2 | 6.26 | 2.28 | 1.83 | 4.29 | 44.00 | 81 | 9.5 | 3.8 | ## | 43 | 4.5 | 15.8 |
| 4466 | 1 | 2 | 53 | 23.12 | 1 | 1 | 90.0 | 72 | 1 | 5.50 | 2.15 | 1.04 | 1.56 | 27.00 | 17 | 11.1 | 1.4 | 49 | 77 | 3.9 | 12.6 |
| 4475 | 1 | 1 | 40 | 23.88 | 1 | 1 | 120.0 | 80 | 1 | 3.66 | 3.00 | 1.71 | 3.79 | 31.00 | 35 | 5.6 | 1.9 | 58 | 102 | 5.0 | 14.8 |
| 4487 | 1 | 1 | 49 | 24.00 | 2 | 2 | 130.0 | 85 | 2 | 5.92 | 1.36 | 1.32 | 3.50 | 32.00 | 45 | 15.3 | 2.9 | 14 | 95 | 4.7 | 15.6 |
| 4490 | 1 | 2 | 33 | 20.58 | 2 | 2 | 100.0 | 70 | 2 | 3.53 | 1.45 | .95 | 3.28 | 23.00 | 37 | 16.7 | 3.1 | 49 | 61 | 5.8 | 11.1 |
| 4498 | 1 | 2 | 51 | 22.10 | 1 | 2 | 130.0 | 90 | 1 | 5.25 | 3.98 | 1.20 | 3.64 | 13.00 | 33 | 13.5 | 3.6 | 14 | 54 | 5.2 | 15.0 |
| 4500 | 1 | 1 | 36 | 22.78 | 2 | 2 | 120.0 | 80 | 2 | 5.70 | 2.95 | 1.48 | 3.27 | 38.00 | 31 | 4.0 | 1.4 | ## | 102 | 5.2 | 12.0 |
| 4504 | 1 | 1 | 40 | 23.24 | 1 | 1 | 120.0 | 80 | 1 | 2.13 | 1.85 | 1.13 | 2.32 | 30.00 | 16 | 9.3 | 1.1 | 37 | 98 | 5.2 | 14.9 |
| 4505 | 1 | 2 | 49 | 23.61 | 1 | 2 | 110.0 | 70 | 1 | 6.07 | 2.04 | 1.50 | 4.17 | 11.00 | 14 | 19.4 | 4.4 | 50 | 61 | 5.8 | 15.6 |
| 4531 | 1 | 2 | 43 | 22.86 | 2 | 2 | 112.0 | 70 | 2 | 6.37 | 4.82 | 1.84 | 1.59 | 24.00 | 36 | 25.7 | 2.6 | 38 | 60 | 6.0 | 12.0 |
| 4540 | 1 | 1 | 60 | 25.00 | 2 | 2 | 105.0 | 70 | 1 | 2.99 | .95 | 1.03 | 1.99 | 11.00 | 16 | 3.7 | 3.8 | 52 | 67 | 4.5 | 14.0 |
| 4576 | 1 | 1 | 29 | 25.00 | 2 | 2 | 130.0 | 90 | 2 | 5.39 | 3.16 | 1.08 | 3.62 | 27.00 | 21 | 5.1 | 4.0 | 54 | 93 | 4.4 | 14.9 |
| 4580 | 1 | 1 | 44 | 21.46 | 2 | 2 | 112.0 | 80 | 1 | 3.84 | 3.53 | 1.54 | 3.17 | 34.00 | 23 | 7.1 | 1.6 | 43 | 78 | 4.8 | 13.9 |
| 4594 | 1 | 2 | 28 | 22.10 | 1 | 1 | 110.0 | 70 | 1 | 2.89 | .71 | .99 | 1.34 | 27.00 | 18 | 9.3 | 2.1 | 49 | 106 | 4.2 | 11.8 |
| 4605 | 1 | 1 | 54 | 23.66 | 2 | 2 | 110.0 | 75 | 1 | 6.21 | 1.91 | 1.11 | 4.31 | 44.00 | 52 | 16.4 | 1.6 | 55 | 58 | 5.7 | 15.5 |
| 4607 | 1 | 1 | 28 | 25.00 | 2 | 2 | 120.0 | 80 | 2 | 5.20 | 4.67 | 1.45 | 3.79 | 23.00 | 35 | 14.3 | 3.2 | ## | 42 | 5.2 | 14.3 |
| 4654 | 1 | 2 | 43 | 26.00 | 2 | 2 | 120.0 | 80 | 2 | 5.65 | .40 | 1.42 | 2.45 | 30.00 | 18 | 8.6 | 3.8 | 11 | 69 | 5.3 | 12.1 |
| 4657 | 1 | 1 | 51 | 23.59 | 2 | 2 | 120.0 | 80 | 2 | 9.75 | 2.47 | 1.27 | 2.23 | 52.00 | 51 | 20.0 | 2.4 | 48 | 109 | 7.5 | 20.0 |
| 4658 | 1 | 1 | 54 | 24.00 | 2 | 2 | 160.0 | 90 | 1 | 6.65 | .86 | 1.39 | 4.46 | 44.00 | 34 | 5.4 | 2.6 | 13 | 91 | 5.0 | 15.1 |
| 4659 | 1 | 1 | 22 | 22.04 | 2 | 2 | 120.0 | 80 | 1 | 4.95 | .90 | 1.32 | 2.35 | 17.00 | 30 | 14.5 | 2.4 | 25 | 54 | 4.2 | 14.7 |
| 4677 | 1 | 2 | 55 | 23.05 | 2 | 2 | 110.0 | 70 | 2 | 3.70 | 1.70 | 1.68 | 1.95 | 25.00 | 24 | 19.7 | 6.1 | 77 | 100 | 5.0 | 14.7 |
| 4678 | 1 | 1 | 54 | 23.67 | 2 | 2 | 110.0 | 70 | 2 | 5.60 | 1.52 | 1.13 | 3.26 | 20.00 | 36 | 19.6 | 2.2 | 36 | 63 | 5.3 | 11.2 |
| 4681 | 1 | 2 | 45 | 21.16 | 2 | 2 | 120.0 | 85 | 1 | 6.96 | 5.69 | 1.73 | 4.14 | 28.00 | 14 | 11.6 | 2.4 | 33 | 88 | 4.8 | 14.2 |
| 4682 | 1 | 1 | 58 | 23.18 | 2 | 2 | 130.0 | 85 | 1 | 1.96 | 1.03 | 1.43 | 3.95 | 25.00 | 37 | 11.0 | 3.4 | 27 | 86 | 5.2 | 15.6 |
| 4688 | 1 | 1 | 56 | 23.88 | 2 | 2 | 120.0 | 80 | 2 | 2.88 | .65 | 1.38 | 3.80 | 18.00 | 18 | 13.7 | 1.7 | 29 | 88 | 4.4 | 15.4 |
| 4703 | 1 | 2 | 53 | 24.00 | 2 | 2 | 120.0 | 80 | 2 | 6.31 | 1.13 | 1.18 | 2.96 | 11.00 | 19 | 14.3 | 2.4 | 13 | 46 | 4.3 | 15.9 |
| 4705 | 1 | 1 | 64 | 24.00 | 2 | 2 | 110.0 | 70 | 2 | 5.39 | 2.04 | .99 | 4.10 | 28.00 | 32 | 21.0 | 3.5 | 40 | 51 | 4.6 | 13.8 |
| 4736 | 1 | 2 | 57 | 20.81 | 1 | 2 | 130.0 | 80 | 1 | 5.25 | 1.25 | .98 | 3.72 | 31.00 | 30 | 12.5 | 4.4 | 40 | 93 | 5.3 | 14.1 |
| 4751 | 1 | 2 | 60 | 22.77 | 2 | 2 | 120.0 | 76 | 2 | 5.67 | 1.78 | 1.26 | 2.29 | 15.00 | 35 | 14.8 | 3.2 | 32 | 103 | 5.3 | 14.6 |
| 4755 | 1 | 2 | 70 | 22.04 | 2 | 2 | 135.0 | 90 | 1 | 6.67 | 1.85 | 1.18 | 4.60 | 45.00 | 31 | 6.0 | 3.9 | 20 | 87 | 6.3 | 16.9 |
| 4762 | 1 | 1 | 26 | 25.00 | 2 | 2 | 120.0 | 70 | 1 | 1.23 | 1.83 | 1.83 | 2.24 | 11.00 | 37 | 11.2 | 1.5 | 47 | 108 | 5.9 | 12.8 |
| 4774 | 1 | 2 | 49 | 23.59 | 2 | 2 | 110.0 | 80 | 1 | 3.12 | 1.47 | 1.15 | 2.35 | 13.00 | 12 | 14.9 | 1.8 | 46 | 63 | 4.5 | 15.1 |
| 4828 | 1 | 2 | 26 | 23.44 | 2 | 2 | 96.0 | 70 | 1 | 4.06 | 1.71 | 1.72 | 3.07 | 40.00 | 36 | 5.6 | 3.1 | 41 | 40 | 4.9 | 15.8 |
| 4845 | 1 | 1 | 23 | 21.45 | 2 | 2 | 100.0 | 70 | 1 | 3.32 | 1.69 | 1.62 | 1.48 | 33.00 | 29 | 10.3 | 2.8 | 14 | 97 | 5.3 | 12.5 |
| 4881 | 1 | 1 | 58 | 23.94 | 2 | 2 | 120.0 | 70 | 1 | 2.95 | .80 | 1.26 | 3.35 | 27.00 | 50 | 8.9 | 3.3 | 39 | 109 | 5.7 | 15.2 |
| 4887 | 1 | 1 | 27 | 23.85 | 2 | 2 | 120.0 | 80 | 2 | 2.07 | 2.31 | 1.29 | 2.23 | 48.00 | 38 | 6.7 | 3.4 | 71 | 92 | 5.5 | 11.9 |
| 4888 | 1 | 1 | 38 | 24.00 | 2 | 2 | 110.0 | 70 | 2 | 5.42 | 2.17 | 1.54 | 2.07 | 15.00 | 31 | 7.3 | 1.5 | 12 | 103 | 4.7 | 12.9 |
| 4890 | 1 | 1 | 39 | 23.05 | 2 | 2 | 100.0 | 70 | 1 | 1.87 | 1.05 | 1.87 | 4.04 | 23.00 | 11 | 10.2 | 4.2 | 25 | 119 | 5.4 | 15.5 |
| 4891 | 1 | 1 | 26 | 25.00 | 2 | 2 | 110.0 | 82 | 2 | 4.83 | 3.03 | 1.77 | 2.89 | 37.00 | 17 | 8.0 | 3.1 | 8 | 81 | 4.5 | 14.7 |
| 4897 | 1 | 1 | 49 | 24.00 | 2 | 2 | 120.0 | 80 | 2 | 6.40 | 2.06 | 1.23 | 4.46 | 43.00 | 21 | 5.4 | 1.2 | 65 | 104 | 5.9 | 13.3 |
| 4899 | 1 | 1 | 55 | 25.00 | 2 | 2 | 106.0 | 70 | 1 | 2.08 | 1.24 | 1.52 | 3.95 | 27.00 | 41 | 16.7 | 1.0 | 50 | 117 | 6.1 | 13.2 |
| 4902 | 1 | 1 | 56 | 21.71 | 2 | 2 | 112.0 | 80 | 1 | 3.12 | .80 | 1.84 | 3.98 | 13.00 | 37 | 11.2 | 3.8 | 24 | 41 | 5.2 | 13.7 |
| 4903 | 1 | 1 | 53 | 23.32 | 2 | 2 | 110.0 | 80 | 2 | 3.98 | 1.79 | 1.67 | 3.81 | 20.00 | 31 | 16.2 | 3.7 | 33 | 38 | 5.9 | 15.3 |
| 4908 | 1 | 1 | 39 | 24.00 | 2 | 2 | 110.0 | 82 | 2 | 2.24 | 1.69 | 1.26 | 2.32 | 12.00 | 19 | 7.7 | 3.2 | 37 | 103 | 5.3 | 13.2 |
| 4917 | 1 | 1 | 33 | 25.00 | 1 | 2 | 100.0 | 60 | 1 | 5.87 | 1.52 | 1.51 | 4.14 | 21.00 | 34 | 11.0 | 2.4 | 43 | 95 | 4.0 | 13.0 |
| 4919 | 1 | 1 | 37 | 23.62 | 2 | 2 | 120.0 | 80 | 1 | 1.78 | .87 | 1.34 | 1.78 | 33.00 | 11 | 13.5 | 3.6 | 29 | 105 | 5.7 | 14.5 |
| 4920 | 1 | 1 | 74 | 22.15 | 2 | 2 | 114.0 | 70 | 1 | 1.97 | 1.20 | 1.53 | 3.30 | 38.00 | 24 | 5.8 | 3.5 | 26 | 63 | 4.3 | 11.9 |
| 4921 | 1 | 1 | 38 | 21.89 | 2 | 2 | 100.0 | 60 | 2 | 3.08 | 1.22 | 1.70 | 2.00 | 35.00 | 20 | 9.4 | 4.2 | 13 | 39 | 5.9 | 15.0 |
| 4925 | 1 | 1 | 29 | 24.00 | 2 | 2 | 130.0 | 84 | 2 | 5.69 | 3.39 | 1.61 | 4.05 | 24.00 | 36 | 11.1 | 1.9 | 38 | 109 | 6.2 | 13.9 |
| 4927 | 1 | 1 | 49 | 21.72 | 2 | 2 | 122.0 | 90 | 1 | 5.24 | 1.44 | 1.00 | 1.67 | 27.00 | 41 | 14.1 | 3.2 | 35 | 121 | 4.1 | 11.8 |
| 4928 | 1 | 1 | 29 | 22.57 | 2 | 2 | 120.0 | 84 | 1 | 3.29 | 1.88 | 1.00 | 1.31 | 38.00 | 24 | 11.6 | 3.9 | 50 | 114 | 5.3 | 15.3 |
| 4929 | 1 | 1 | 27 | 21.67 | 1 | 2 | 100.0 | 70 | 1 | 5.43 | 2.40 | 1.54 | 3.01 | 22.00 | 17 | 7.5 | 1.8 | 43 | 115 | 5.8 | 13.8 |
| 4935 | 1 | 2 | 58 | 22.03 | 2 | 2 | 118.0 | 80 | 1 | 5.32 | 1.46 | 1.58 | 2.85 | 30.00 | 21 | 4.0 | 3.7 | 29 | 54 | 5.7 | 14.8 |
| 4939 | 1 | 2 | 48 | 23.63 | 1 | 2 | 100.0 | 70 | 2 | 5.75 | 2.11 | 1.62 | 4.03 | 35.00 | 22 | 16.2 | 3.4 | 15 | 94 | 6.0 | 13.4 |
| 4940 | 1 | 2 | 57 | 21.87 | 2 | 2 | 116.0 | 80 | 1 | 6.12 | .71 | 1.75 | 2.54 | 27.00 | 33 | 16.1 | 2.4 | 40 | 79 | 5.7 | 12.1 |
| 4944 | 1 | 1 | 42 | 24.00 | 2 | 2 | 110.0 | 76 | 1 | 3.70 | .78 | 1.63 | 3.13 | 13.00 | 35 | 16.4 | 3.1 | 46 | 73 | 5.3 | 14.1 |
| 4945 | 1 | 1 | 37 | 24.00 | 2 | 2 | 120.0 | 80 | 2 | 4.98 | .69 | 1.84 | 3.65 | 14.00 | 12 | 11.8 | 2.7 | 41 | 40 | 5.0 | 14.4 |
| 4947 | 1 | 1 | 27 | 24.00 | 2 | 2 | 90.0 | 60 | 1 | 5.29 | .92 | 1.68 | 3.67 | 27.00 | 16 | 15.6 | 3.0 | 25 | 58 | 4.9 | 11.5 |
| 4952 | 1 | 1 | 54 | 24.00 | 2 | 2 | 110.0 | 80 | 2 | 5.66 | 1.75 | 1.13 | 2.26 | 12.00 | 12 | 13.0 | 2.2 | 96 | 82 | 5.8 | 14.1 |
| 4953 | 1 | 1 | 49 | 23.99 | 2 | 2 | 120.0 | 80 | 1 | 5.63 | 1.69 | 1.37 | 3.29 | 35.00 | 14 | 3.9 | 3.1 | 12 | 52 | 5.4 | 14.7 |
| 4954 | 1 | 1 | 54 | 25.00 | 2 | 2 | 110.0 | 80 | 1 | 6.68 | .62 | 1.74 | 4.66 | 50.00 | 49 | 16.4 | 3.6 | 48 | 56 | 4.1 | 11.2 |
| 4957 | 1 | 1 | 57 | 20.24 | 2 | 2 | 150.0 | 70 | 2 | 1.66 | 1.14 | 1.09 | 2.61 | 10.00 | 12 | 14.4 | 2.7 | 24 | 41 | 5.4 | 11.9 |
| 4958 | 1 | 2 | 51 | 23.05 | 2 | 2 | 120.0 | 80 | 2 | 3.50 | 1.09 | 1.17 | 3.47 | 12.00 | 26 | 13.7 | 3.7 | 33 | 42 | 4.1 | 11.6 |
| 4959 | 1 | 2 | 30 | 21.99 | 2 | 2 | 114.0 | 80 | 2 | 6.80 | 2.08 | 1.61 | 4.89 | 43.00 | 12 | 12.4 | 1.6 | 46 | 104 | 4.5 | 14.0 |
| 4965 | 1 | 1 | 58 | 24.00 | 2 | 2 | 108.0 | 80 | 1 | 3.80 | 1.98 | 1.64 | 2.35 | 29.00 | 36 | 4.4 | 4.0 | 50 | 64 | 4.1 | 11.7 |
| 4967 | 1 | 2 | 51 | 23.88 | 2 | 2 | 110.0 | 70 | 1 | 5.26 | 1.63 | 1.07 | 2.03 | 27.00 | 47 | 12.8 | 1.6 | 38 | 120 | 5.3 | 12.2 |
| 4970 | 1 | 1 | 60 | 22.32 | 1 | 2 | 120.0 | 70 | 1 | 5.40 | 1.00 | 1.20 | 1.50 | 24.00 | 37 | 10.4 | 3.8 | 15 | 51 | 4.3 | 13.4 |
| 5068 | 1 | 1 | 40 | 21.37 | 2 | 2 | 130.0 | 90 | 1 | 4.58 | .69 | 1.63 | 2.24 | 12.00 | 12 | 11.5 | 3.7 | 12 | 108 | 4.2 | 13.1 |
| 5071 | 1 | 1 | 34 | 24.00 | 1 | 2 | 90.0 | 60 | 1 | 3.45 | .80 | 1.17 | 2.15 | 23.00 | 36 | 4.7 | 3.7 | 27 | 102 | 4.3 | 13.0 |
| 5082 | 1 | 1 | 66 | 25.00 | 1 | 2 | 126.0 | 90 | 1 | 1.20 | 1.41 | 1.75 | 2.78 | 17.00 | 22 | 8.5 | 3.4 | 65 | 100 | 4.0 | 14.7 |
| 5089 | 1 | 1 | 23 | 23.92 | 2 | 2 | 120.0 | 80 | 1 | 1.24 | 1.52 | 1.05 | 3.50 | 18.00 | 11 | 9.6 | 3.6 | 43 | 107 | 4.7 | 11.8 |
| 5094 | 1 | 1 | 52 | 22.23 | 2 | 2 | 100.0 | 80 | 1 | 3.19 | 1.35 | 1.02 | 2.16 | 19.00 | 38 | 6.9 | 3.5 | 22 | 119 | 5.3 | 15.6 |
| 5096 | 1 | 1 | 45 | 25.00 | 2 | 2 | 130.0 | 86 | 1 | 1.16 | 1.83 | .88 | 2.51 | 41.00 | 35 | 15.5 | 3.7 | 43 | 94 | 4.8 | 11.2 |
| 5097 | 1 | 2 | 56 | 21.91 | 2 | 2 | 114.0 | 70 | 1 | 5.92 | 1.56 | 1.04 | 2.82 | 24.00 | 39 | 11.7 | 1.6 | 27 | 43 | 5.2 | 13.8 |
| 5109 | 1 | 1 | 45 | 23.24 | 2 | 2 | 120.0 | 94 | 2 | 1.96 | 1.66 | 1.84 | 4.03 |  | 17 | 10.8 | 2.9 | 16 | 101 | 4.4 | 11.8 |
| 5114 | 1 | 1 | 50 | 23.48 | 2 | 2 | 130.0 | 82 | 2 | 6.15 | 1.93 | 1.82 | 3.16 | 31.00 | 33 | 15.7 | 1.0 | 51 | 62 | 5.3 | 11.4 |
| 5117 | 1 | 1 | 25 | 24.00 | 2 | 2 | 120.0 | 84 | 1 | 5.05 | 2.09 | .96 | 3.25 | 35.00 | 20 | 7.4 | 2.9 | 16 | 57 | 4.3 | 14.2 |
| 5118 | 1 | 2 | 49 | 22.91 | 2 | 2 | 100.0 | 70 | 2 | 5.86 | .78 | 1.32 | 3.80 | 39.00 | 14 | 13.6 | 2.6 | 31 | 74 | 5.8 | 11.9 |
| 5119 | 1 | 1 | 72 | 22.02 | 1 | 1 | 100.0 | 76 | 1 | 5.64 | 1.57 | 1.64 | 3.75 | 13.00 | 29 | 12.5 | 2.9 | 9 | 121 | 5.2 | 13.2 |
| 5123 | 1 | 1 | 69 | 23.57 | 2 | 2 | 130.0 | 80 | 1 | 4.98 | 3.56 | 1.61 | 2.35 | 12.00 | 47 | 5.9 | 2.6 | 35 | 115 | 6.7 | 18.0 |
| 5128 | 1 | 1 | 32 | 23.72 | 2 | 2 | 120.0 | 80 | 1 | 2.50 | 1.20 | 1.34 | 3.34 | 7.00 | 15 | 4.3 | 2.9 | 46 | 64 | 4.8 | 11.8 |
| 5135 | 1 | 1 | 26 | 23.25 | 2 | 2 | 100.0 | 78 | 2 | 5.71 | 1.72 | 1.30 | 3.82 | 6.00 | 26 | 18.2 | 2.5 | 41 | 123 | 5.0 | 14.7 |
| 5137 | 1 | 1 | 43 | 22.98 | 1 | 1 | 118.0 | 80 | 1 | 1.73 | 1.06 | 1.34 | 2.74 | 13.00 | 35 | 14.1 | 2.8 | 45 | 118 | 5.2 | 16.0 |
| 5163 | 1 | 1 | 56 | 23.72 | 2 | 2 | 100.0 | 70 | 1 | 2.55 | 1.23 | 1.65 | 1.96 | 11.00 | 40 | 10.6 | 2.9 | 29 | 103 | 4.8 | 15.0 |
| 5164 | 1 | 1 | 30 | 20.24 | 1 | 1 | 120.0 | 90 | 1 | 5.56 | 1.85 | 1.11 | 2.04 | 16.00 | 40 | 5.1 | 1.6 | 43 | 81 | 5.8 | 12.2 |
| 5166 | 1 | 1 | 35 | 25.00 | 1 | 1 | 120.0 | 80 | 2 | 1.60 | 1.86 | 1.04 | 1.76 | 28.00 | 20 | 16.9 | 1.3 | 35 | 49 | 5.5 | 13.3 |
| 5185 | 1 | 1 | 26 | 26.00 | 2 | 2 | 125.0 | 92 | 1 | 5.14 | 1.00 | 1.21 | 3.53 | 15.00 | 34 | 14.0 | 3.1 | ## | 98 | 5.7 | 13.8 |
| 5235 | 1 | 1 | 32 | 25.00 | 2 | 2 | 120.0 | 84 | 2 | 4.92 | 2.65 | .95 | 3.65 | 17.00 | 12 | 5.3 | 4.4 | 40 | 94 | 5.0 | 13.7 |
| 5237 | 1 | 1 | 34 | 25.00 | 1 | 1 | 140.0 | 92 | 2 | 6.93 | 4.74 | .81 | 4.28 | 36.00 | 34 | 8.4 | 2.7 | 64 | 54 | 4.0 | 14.4 |
| 5241 | 1 | 1 | 32 | 23.12 | 2 | 2 | 110.0 | 80 | 2 | 5.86 | 2.83 | 1.53 | 3.73 | 57.00 | 19 | 9.1 | 2.4 | ## | 147 | 4.7 | 11.7 |
| 5253 | 1 | 1 | 37 | 24.00 | 2 | 2 | 120.0 | 80 | 2 | 1.38 | 1.67 | 1.63 | 2.60 | 37.00 | 29 | 16.8 | 1.9 | 57 | 69 | 5.6 | 13.3 |
| 5312 | 1 | 1 | 55 | 23.80 | 2 | 2 | 130.0 | 85 | 2 | 7.62 | 2.06 | 1.66 | 5.86 | ##### | 78 | 7.9 | 1.0 | 97 | 108 | 6.9 | 19.0 |
| 5313 | 1 | 2 | 23 | 22.04 | 1 | 2 | 114.0 | 70 | 1 | 2.53 | 1.01 | 1.19 | 2.70 | 39.00 | 34 | 10.4 | 4.0 | 28 | 146 | 4.4 | 11.3 |
| 5314 | 1 | 1 | 61 | 23.85 | 2 | 2 | 120.0 | 80 | 2 | 5.84 | 1.75 | 1.76 | 2.99 | 53.00 | 54 | 4.7 | 3.9 | 68 | 38 | 4.5 | 12.6 |
| 5318 | 1 | 2 | 48 | 24.00 | 2 | 2 | 90.0 | 60 | 2 | 5.59 | 2.46 | 1.69 | 3.88 | 11.00 | 39 | 10.5 | 3.8 | 42 | 69 | 4.0 | 12.4 |
| 5330 | 1 | 1 | 31 | 24.00 | 1 | 2 | 110.0 | 70 | 2 | 5.41 | 1.38 | 1.02 | 3.08 | 16.00 | 20 | 12.9 | 3.3 | 10 | 43 | 4.3 | 14.9 |
| 5337 | 1 | 2 | 48 | 24.00 | 2 | 2 | 100.0 | 60 | 1 | 4.09 | .81 | 1.79 | 3.72 | 6.00 | 35 | 8.8 | 3.1 | 39 | 98 | 6.1 | 15.7 |
| 5343 | 1 | 2 | 43 | 23.23 | 1 | 2 | 140.0 | 95 | 1 | 4.50 | .68 | 1.22 | 3.66 | 22.00 | 40 | 13.7 | 3.3 | 32 | 114 | 4.4 | 13.8 |
| 5353 | 1 | 2 | 24 | 20.80 | 2 | 2 | 100.0 | 60 | 1 | 4.27 | .76 | 1.84 | 3.53 | 10.00 | 20 | 6.4 | 4.0 | 23 | 78 | 6.0 | 13.8 |
| 5356 | 1 | 1 | 53 | 23.60 | 2 | 2 | 100.0 | 70 | 1 | 3.45 | 2.08 | 1.72 | 1.87 | 11.00 | 45 | 13.6 | 2.1 | 26 | 107 | 5.2 | 16.0 |
| 5362 | 1 | 1 | 25 | 23.96 | 2 | 2 | 110.0 | 70 | 1 | 5.11 | 2.13 | 1.22 | 3.43 | 37.00 | 14 | 10.4 | 2.4 | 49 | 99 | 5.3 | 12.6 |
| 5388 | 1 | 1 | 68 | 24.00 | 2 | 2 | 150.0 | 90 | 1 | 5.29 | 4.21 | 1.21 | 2.35 | 33.00 | 57 | 8.8 | 4.4 | ## | 115 | 4.8 | 11.6 |
| 5406 | 1 | 2 | 31 | 23.67 | 2 | 2 | 110.0 | 70 | 1 | 4.99 | .61 | 1.56 | 4.03 | 27.00 | 16 | 7.3 | 1.4 | 26 | 41 | 4.4 | 11.2 |
| 5410 | 1 | 2 | 52 | 24.00 | 2 | 2 | 140.0 | 85 | 1 | 1.04 | 4.55 | 1.21 | 4.03 | 28.00 | 35 | 13.4 | 4.0 | 49 | 114 | 5.4 | 12.3 |
| 5411 | 1 | 2 | 29 | 22.33 | 2 | 2 | 110.0 | 70 | 2 | 1.05 | 1.15 | 1.58 | 4.42 | 10.00 | 41 | 6.0 | 1.3 | 27 | 120 | 5.6 | 13.8 |
| 5413 | 1 | 1 | 41 | 23.94 | 1 | 2 | 110.0 | 80 | 2 | 5.96 | .97 | .96 | 4.27 | 23.00 | 14 | 12.9 | 3.2 | 15 | 52 | 4.1 | 13.8 |
| 5416 | 1 | 1 | 45 | 20.68 | 1 | 2 | 100.0 | 70 | 1 | 1.38 | .67 | .96 | 2.95 | 30.00 | 12 | 15.9 | 2.0 | 17 | 70 | 5.2 | 11.8 |
| 5417 | 1 | 2 | 56 | 24.00 | 2 | 2 | 110.0 | 70 | 1 | 1.33 | .72 | 1.09 | 3.96 | 10.00 | 16 | 3.7 | 2.7 | 41 | 115 | 5.3 | 11.9 |
| 5425 | 1 | 1 | 48 | 23.46 | 2 | 2 | 120.0 | 80 | 1 | 4.55 | 1.31 | 1.42 | 3.08 | 16.00 | 40 | 10.6 | 3.5 | 23 | 109 | 4.4 | 14.2 |
| 5436 | 1 | 2 | 53 | 22.59 | 2 | 2 | 140.0 | 94 | 1 | 5.43 | 1.02 | 1.23 | 3.06 | 32.00 | 15 | 11.7 | 4.1 | 37 | 52 | 4.3 | 11.9 |
| 5437 | 1 | 2 | 33 | 24.00 | 1 | 2 | 110.0 | 70 | 2 | 4.02 | 1.18 | 1.40 | 1.37 | 11.00 | 29 | 5.1 | 3.4 | 44 | 47 | 5.0 | 14.3 |
| 5442 | 1 | 1 | 24 | 23.66 | 1 | 2 | 110.0 | 76 | 2 | 2.80 | 1.63 | 1.14 | 3.79 | 28.00 | 26 | 14.0 | 1.7 | 15 | 57 | 5.6 | 11.4 |
| 5446 | 1 | 1 | 41 | 22.49 | 2 | 2 | 114.0 | 80 | 2 | 2.60 | 1.33 | 1.50 | 3.71 | 43.00 | 37 | 4.9 | 2.9 | 91 | 84 | 4.6 | 14.8 |
| 5451 | 1 | 2 | 44 | 26.00 | 2 | 2 | 130.0 | 80 | 2 | 5.30 | 1.95 | 1.77 | 1.57 | 25.00 | 27 | 5.2 | 3.8 | 31 | 116 | 5.4 | 12.0 |
| 5455 | 1 | 2 | 32 | 24.00 | 2 | 2 | 110.0 | 70 | 1 | 1.10 | 1.54 | .98 | 3.94 | 26.00 | 15 | 15.3 | 2.8 | 31 | 75 | 4.6 | 15.4 |
| 5456 | 1 | 2 | 51 | 24.00 | 1 | 2 | 110.0 | 80 | 2 | 5.58 | .68 | 1.80 | 1.80 | 16.00 | 10 | 11.1 | 1.1 | 26 | 113 | 5.8 | 14.9 |
| 5457 | 1 | 1 | 37 | 22.49 | 2 | 2 | 110.0 | 80 | 1 | 1.48 | 1.38 | 1.08 | 2.69 | 23.00 | 18 | 16.2 | 3.7 | 20 | 44 | 4.6 | 14.9 |
| 5461 | 1 | 2 | 32 | 21.34 | 1 | 2 | 100.0 | 70 | 1 | 1.32 | 1.15 | 1.73 | 2.04 | 22.00 | 12 | 14.4 | 3.0 | 17 | 86 | 4.9 | 12.1 |
| 5463 | 1 | 1 | 40 | 23.74 | 2 | 2 | 110.0 | 80 | 2 | 4.86 | 1.39 | 1.61 | 3.19 | 26.00 | 37 | 17.1 | 2.5 | 9 | 92 | 5.6 | 13.8 |
| 5467 | 1 | 1 | 43 | 21.22 | 2 | 2 | 120.0 | 80 | 1 | 5.64 | 1.75 | 1.10 | 3.78 | 26.00 | 19 | 16.3 | 3.5 | 21 | 126 | 4.8 | 13.4 |
| 5476 | 1 | 1 | 24 | 22.41 | 2 | 2 | 130.0 | 80 | 1 | 5.26 | 1.58 | .96 | 1.55 | 36.00 | 15 | 14.4 | 2.7 | 41 | 56 | 4.3 | 13.4 |
| 5484 | 1 | 1 | 59 | 22.10 | 2 | 2 | 114.0 | 70 | 2 | 5.65 | 1.01 | 1.43 | 3.27 | 22.00 | 35 | 14.9 | 1.5 | 9 | 108 | 4.8 | 12.5 |
| 5532 | 1 | 1 | 32 | 21.88 | 2 | 2 | 116.0 | 70 | 2 | 4.58 | 1.67 | 1.05 | 2.49 | 7.00 | 19 | 9.4 | 3.9 | 9 | 58 | 4.2 | 14.2 |
| 5535 | 1 | 1 | 56 | 23.95 | 2 | 2 | 110.0 | 70 | 2 | 2.10 | 1.05 | 1.76 | 1.35 | 27.00 | 24 | 9.1 | 2.8 | 33 | 39 | 5.2 | 6.3 |
| 5546 | 1 | 2 | 27 | 20.52 | 2 | 2 | 100.0 | 60 | 1 | 5.49 | 1.86 | 1.02 | 1.31 | 20.00 | 20 | 12.5 | 1.5 | 17 | 93 | 4.8 | 15.4 |
| 5549 | 1 | 1 | 53 | 24.00 | 2 | 2 | 130.0 | 90 | 1 | 2.13 | 2.36 | 1.45 | 3.85 | 35.00 | 35 | 15.6 | 4.2 | 46 | 50 | 5.7 | 14.8 |
| 5559 | 1 | 1 | 57 | 24.00 | 2 | 2 | 130.0 | 80 | 2 | 6.06 | 1.99 | 1.51 | 4.26 | 25.00 | 37 | 16.5 | 1.6 | 41 | 45 | 5.3 | 12.4 |
| 5560 | 1 | 1 | 56 | 23.18 | 2 |  | 110.0 | 70 | 2 | 5.48 | .95 | .96 | 3.39 | 26.00 | 28 | 10.2 | 1.9 | 76 | 110 | 4.7 | 14.8 |
| 5566 | 1 | 2 | 55 | 23.74 | 1 | 2 | 132.0 | 80 | 2 | 5.21 | 1.27 | .70 | 2.08 | 23.00 | 28 | 8.9 | 1.1 | 35 | 77 | 4.6 | 12.5 |
| 5571 | 1 | 1 | 60 | 24.00 | 2 | 2 | 124.0 | 70 | 2 | 4.14 | 1.47 | 1.32 | 2.65 | 27.00 | 36 | 7.4 | 3.5 | 36 | 101 | 6.2 | 11.4 |
| 5575 | 1 | 1 | 52 | 23.84 | 1 | 2 | 120.0 | 76 | 2 | 3.27 | .77 | 1.36 | 1.63 | 33.00 | 20 | 10.2 | 3.2 | 44 | 95 | 5.2 | 11.3 |
| 5582 | 1 | 2 | 66 | 25.00 | 1 | 1 | 150.0 | 90 | 1 | 3.49 | 2.10 | 1.12 | 1.85 | 13.00 | 12 | 14.9 | 4.0 | 9 | 78 | 4.6 | 13.8 |
| 5592 | 1 | 1 | 23 | 24.00 | 1 | 1 | 110.0 | 70 | 2 | 1.01 | 1.19 | 1.27 | 3.67 | 20.00 | 11 | 4.5 | 2.0 | 24 | 115 | 4.5 | 15.4 |
| 5595 | 1 | 1 | 28 | 23.67 | 2 | 2 | 150.0 | 85 | 2 | 6.54 | 6.41 | 1.52 | 1.89 | 20.00 | 40 | 8.8 | 1.2 | 89 | 126 | 6.6 | 11.6 |
| 5599 | 1 | 1 | 38 | 21.97 | 2 | 2 | 100.0 | 64 | 1 | 6.53 | 1.19 | 1.34 | 4.62 | 42.00 | 16 | 13.7 | 4.3 | 13 | 80 | 5.8 | 15.9 |
| 5649 | 1 | 2 | 27 | 22.50 | 2 | 2 | 120.0 | 84 | 1 | 2.81 | .96 | 1.61 | 2.24 | 21.00 | 31 | 13.7 | 1.0 | 29 | 108 | 5.5 | 13.3 |
| 5667 | 1 | 2 | 83 | 26.00 | 2 | 2 | 150.0 | 90 | 1 | 5.24 | 4.27 | 1.46 | 2.20 | 31.00 | 37 | 14.4 | 3.3 | 53 | 81 | 4.2 | 14.3 |
| 5670 | 1 | 2 | 46 | 26.00 | 2 | 2 | 120.0 | 80 | 2 | 2.10 | .88 | 1.36 | 2.80 | 12.00 | 31 | 16.6 | 3.7 | 49 | 60 | 6.0 | 15.3 |
| 5691 | 1 | 2 | 34 | 24.00 | 1 | 2 | 100.0 | 70 | 1 | 3.56 | 1.25 | 1.32 | 2.09 | 24.00 | 27 | 5.0 | 3.9 | 45 | 84 | 4.3 | 13.7 |
| 5701 | 1 | 1 | 47 | 23.81 | 2 | 2 | 140.0 | 90 | 2 | 4.49 | 1.93 | 1.14 | 3.00 | 30.00 | 36 | 13.2 | 3.4 | ## | 99 | 5.4 | 15.5 |
| 5704 | 1 | 1 | 35 | 23.94 | 2 | 2 | 130.0 | 85 | 1 | 6.57 | 1.48 | 1.69 | 4.88 | 47.00 | 40 | 11.0 | 2.4 | 12 | 96 | 4.2 | 6.6 |
| 5705 | 1 | 1 | 27 | 23.05 | 2 | 2 | 120.0 | 80 | 2 | 6.52 | 1.70 | 1.02 | 5.10 | 49.00 | 38 | 27.0 | 7.0 | 21 | 82 | 4.4 | 15.1 |
| 5707 | 1 | 2 | 62 | 22.21 | 2 | 2 | 120.0 | 80 | 2 | 5.43 | .86 | 1.36 | 3.08 | 31.00 | 38 | 5.7 | 2.3 | 24 | 68 | 8.8 | 15.3 |
| 5736 | 1 | 1 | 71 | 26.00 | 2 | 2 | 150.0 | 90 | 1 | 2.50 | 1.15 | 1.70 | 1.80 | 39.00 | 14 | 7.1 | 2.3 | 19 | 75 | 5.9 | 15.5 |
| 5749 | 1 | 1 | 53 | 23.88 | 2 | 2 | 140.0 | 90 | 2 | 4.90 | 5.23 | 1.80 | 2.86 | 27.00 | 20 | 11.8 | 3.7 | 34 | 70 | 5.2 | 13.3 |
| 5756 | 1 | 2 | 46 | 23.73 | 2 | 2 | 120.0 | 90 | 1 | 2.71 | .68 | 1.24 | 2.25 | 15.00 | 28 | 3.6 | 3.9 | 39 | 82 | 7.4 | 19.8 |
| 5769 | 1 | 2 | 57 | 24.01 | 2 | 2 | 110.0 | 70 | 1 | 4.72 | 1.33 | 1.08 | 3.02 | 26.00 | 40 | 7.4 | 1.3 | 9 | 83 | 5.7 | 13.8 |
| 5779 | 1 | 2 | 79 | 27.00 | 2 | 2 | 140.0 | 90 | 2 | 3.07 | 1.96 | 1.41 | 2.37 | 39.00 | 47 | 6.7 | 1.2 | 29 | 66 | 5.2 | 14.4 |
| 5790 | 1 | 2 | 36 | 25.00 | 2 | 2 | 120.0 | 80 | 2 | 2.68 | 1.38 | 1.27 | 2.62 | 15.00 | 21 | 14.1 | 2.6 | 33 | 44 | 4.8 | 13.8 |
| 6358 | 1 | 1 | 55 | 25.00 | 2 | 2 | 100.0 | 76 | 1 | 2.98 | .80 | 1.83 | 2.60 | 34.00 | 69 | 14.9 | 2.4 | 34 | 42 | 4.9 | 13.1 |
| 6361 | 1 | 1 | 50 | 22.49 | 2 | 2 | 120.0 | 80 | 1 | 6.08 | .84 | 1.62 | 4.32 | 41.00 | 20 | 15.5 | 3.4 | 8 | 100 | 4.3 | 13.4 |
| 6376 | 1 | 1 | 30 | 23.74 | 2 | 2 | 120.0 | 90 | 2 | 5.55 | 1.59 | 1.26 | 1.51 | 19.00 | 12 | 13.9 | 1.3 | 24 | 42 | 5.8 | 13.5 |
| 6386 | 1 | 1 | 56 | 21.71 | 2 | 2 | 112.0 | 80 | 1 | 4.20 | .62 | 1.73 | 3.45 | 11.00 | 31 | 11.6 | 2.7 | 38 | 119 | 4.6 | 15.0 |
| 6390 | 1 | 1 | 61 | 23.03 | 2 | 2 | 120.0 | 88 | 1 | 2.67 | 3.63 | 1.24 | 3.62 | 36.00 | 34 | 4.9 | 1.0 | 18 | 100 | 5.9 | 11.2 |
| 6394 | 1 | 2 | 51 | 21.77 | 2 | 2 | 108.0 | 80 | 1 | 2.83 | 4.25 | 1.02 | 2.45 | 10.00 | 12 | 9.2 | 3.5 | 9 | 71 | 5.9 | 15.8 |
| 6400 | 1 | 1 | 61 | 25.00 |  | 2 | 120.0 | 80 | 1 | 6.21 | 6.14 | 1.72 | 3.45 | 38.00 | 24 | 13.3 | 1.7 | 27 | 105 | 7.7 | 19.3 |
| 6405 | 1 | 1 | 41 | 22.49 | 1 | 1 | 130.0 | 90 | 1 | 3.18 | 2.33 | 1.73 | 1.61 | 17.00 | 41 | 14.3 | 3.7 | 10 | 111 | 5.4 | 13.2 |
| 6409 | 1 | 1 | 61 | 22.72 | 2 | 2 | 100.0 | 70 | 1 | 5.09 | .99 | 1.26 | 1.36 | 22.00 | 9 | 8.6 | 3.0 | 10 | 121 | 6.0 | 12.8 |
| 6412 | 1 | 1 | 40 | 21.01 | 2 | 2 | 112.0 | 75 | 2 | 5.47 | 1.53 | 1.44 | 2.84 | 23.00 | 17 | 9.4 | 2.6 | ## | 113 | 5.4 | 12.1 |
| 6422 | 1 | 1 | 36 | 22.89 | 2 | 2 | 112.0 | 70 | 1 | 2.42 | 1.64 | 1.65 | 3.86 | 36.00 | 36 | 13.8 | 1.2 | 30 | 98 | 4.6 | 11.1 |
| 6427 | 1 | 1 | 34 | 22.15 | 2 | 2 | 100.0 | 70 | 1 | 1.46 | 1.64 | 1.14 | 2.85 | 28.00 | 67 | 8.9 | 2.1 | 32 | 126 | 4.4 | 15.3 |
| 6437 | 1 | 1 | 29 | 23.50 | 2 | 2 | 110.0 | 70 | 1 | 1.24 | .83 | 1.56 | 2.87 | 38.00 | 40 | 6.2 | 2.5 | 11 | 99 | 4.1 | 11.9 |
| 6447 | 1 | 1 | 28 | 21.47 | 2 | 2 | 100.0 | 70 | 1 | 3.44 | .73 | 1.04 | 1.65 | 11.00 | 22 | 5.5 | 3.8 | 64 | 71 | 5.2 | 13.8 |
| 6453 | 1 | 1 | 28 | 23.05 | 1 | 2 | 110.0 | 70 | 2 | 2.95 | 1.40 | 1.04 | 3.12 | 28.00 | 24 | 4.4 | 2.5 | 45 | 76 | 4.4 | 11.2 |
| 6454 | 1 | 1 | 52 | 23.51 | 2 | 2 | 120.0 | 76 | 2 | 5.22 | .88 | 1.45 | 4.02 | 37.00 | 24 | 5.1 | 2.4 | 55 | 49 | 5.8 | 11.2 |
| 6455 | 1 | 1 | 23 | 23.88 | 2 | 2 | 110.0 | 78 | 1 | 5.21 | 1.53 | 1.25 | 1.62 | 39.00 | 32 | 10.4 | 3.2 | 7 | 61 | 4.7 | 12.0 |
| 6458 | 1 | 1 | 43 | 22.65 | 2 | 2 | 130.0 | 90 | 2 | 3.71 | 1.66 | 1.16 | 4.46 | 38.00 | 47 | 12.8 | 2.8 | 56 | 97 | 9.5 | 29.6 |
| 6472 | 1 | 1 | 27 | 22.99 | 1 | 2 | 90.0 | 60 | 1 | 1.51 | 1.01 | 1.25 | 3.10 | 13.00 | 35 | 15.9 | 4.4 | ## | 52 | 5.4 | 14.4 |
| 6473 | 1 | 1 | 38 | 22.10 | 2 | 2 | 130.0 | 70 | 1 | 5.46 | 1.66 | 1.22 | 1.62 | 14.00 | 18 | 9.1 | 4.2 | 45 | 89 | 6.1 | 12.1 |
| 6476 | 1 | 1 | 31 | 23.05 | 2 | 2 | 110.0 | 70 | 1 | 1.46 | 1.72 | 1.83 | 3.04 | 13.00 | 43 | 16.7 | 2.8 | 17 | 66 | 5.7 | 13.0 |
| 6480 | 1 | 1 | 38 | 23.46 | 2 | 2 | 120.0 | 80 | 1 | 2.82 | 1.23 | 1.20 | 3.07 | 17.00 | 26 | 13.8 | 4.1 | 46 | 86 | 5.2 | 12.8 |
| 6482 | 1 | 2 | 38 | 23.74 | 1 | 2 | 100.0 | 70 | 1 | 3.32 | 1.13 | 1.53 | 3.15 | 17.00 | 32 | 16.5 | 2.4 | 38 | 49 | 4.5 | 15.0 |
| 6485 | 1 | 1 | 46 | 24.00 | 2 | 2 | 110.0 | 70 | 2 | 5.26 | .68 | 1.53 | 2.27 | 64.00 | 45 | 6.4 | 3.4 | ## | 62 | 4.0 | 12.5 |
| 6499 | 1 | 1 | 26 | 23.26 | 2 | 2 | 120.0 | 84 | 2 | 4.00 | 1.13 | 1.47 | 4.01 | 30.00 | 16 | 15.3 | 2.4 | 11 | 67 | 3.5 | 15.2 |
| 6515 | 1 | 2 | 37 | 23.62 | 2 | 2 | 86.0 | 60 | 1 | 2.97 | 1.49 | 1.57 | 3.00 | 11.00 | 12 | 8.4 | 1.9 | 41 | 110 | 4.9 | 12.2 |
| 7108 | 1 | 1 | 50 | 22.53 | 2 | 2 | 120.0 | 80 | 1 | 1.13 | 1.46 | 1.70 | 2.40 | 20.00 | 21 | 11.4 | 2.8 | 26 | 72 | 5.6 | 15.6 |
| 7112 | 1 | 1 | 52 | 23.38 | 2 | 2 | 130.0 | 90 | 2 | 3.45 | 1.33 | 1.64 | 4.85 | 34.00 | 57 | 15.3 | 4.2 | ## | 40 | 4.6 | 13.2 |
| 7119 | 1 | 2 | 46 | 21.01 | 1 | 1 | 120.0 | 80 | 1 | 3.39 | 2.33 | 1.62 | 1.57 | 28.00 | 15 | 14.2 | 1.8 | 38 | 52 | 5.3 | 13.0 |
| 7148 | 1 | 1 | 27 | 23.04 | 2 | 2 | 110.0 | 70 | 1 | 6.48 | 2.13 | 1.55 | 4.75 | 49.00 | 34 | 8.9 | 3.2 | 53 | 60 | 5.3 | 15.9 |
| 7730 | 1 | 1 | 26 | 25.00 | 2 | 2 | 110.0 | 80 | 1 | 6.31 | 2.04 | 1.37 | 4.46 | 50.00 | 21 | 17.2 | 3.8 | 62 | 61 | 4.0 | 14.0 |
| 7735 | 1 | 1 | 57 | 22.92 | 2 | 2 | 110.0 | 80 | 1 | 7.24 | 1.02 | 1.48 | 5.45 | 43.00 | 15 | 12.7 | 1.4 | 45 | 99 | 4.6 | 14.2 |
| 7771 | 1 | 2 | 72 | 23.73 | 2 | 2 | 120.0 | 70 | 1 | 7.63 | 1.75 | 1.69 | 3.26 | 17.00 | 11 | 10.0 | 3.4 | 33 | 53 | 5.6 | 11.8 |
| 7773 | 1 | 2 | 41 | 21.34 | 2 | 2 | 100.0 | 80 | 1 | 1.13 | 2.31 | 1.18 | 1.33 | 38.00 | 24 | 16.3 | 1.6 | 47 | 101 | 5.0 | 14.7 |
| 7778 | 1 | 2 | 53 | 23.42 | 2 | 2 | 110.0 | 70 | 1 | 1.83 | 1.05 | 1.71 | 2.37 | 37.00 | 17 | 9.5 | 1.3 | 10 | 90 | 5.5 | 12.0 |
| 7779 | 1 | 2 | 29 | 23.83 | 2 | 2 | 100.0 | 80 | 1 | 5.52 | .95 | 1.84 | 3.46 | 19.00 | 29 | 10.4 | 3.3 | 27 | 70 | 4.2 | 15.4 |
| 8328 | 1 | 2 | 30 | 23.14 | 2 | 2 | 100.0 | 60 | 1 | 5.31 | .80 | 1.31 | 1.37 | 22.00 | 24 | 7.0 | 2.5 | 49 | 125 | 4.1 | 13.0 |
| 8334 | 1 | 2 | 57 | 23.44 | 1 | 1 | 120.0 | 70 | 1 | 5.33 | .83 | 1.03 | 2.43 | 19.00 | 17 | 9.1 | 1.8 | 17 | 64 | 5.8 | 13.8 |
| 8344 | 1 | 2 | 37 | 23.95 | 2 | 2 | 130.0 | 86 | 2 | 2.09 | 1.27 | 1.84 | 2.78 | 28.00 | 28 | 4.5 | 3.4 | 15 | 78 | 4.0 | 11.6 |
| 8636 | 1 | 1 | 27 | 21.88 | 1 | 1 | 90.0 | 60 | 1 | 4.76 | 1.54 | .98 | 2.37 | 32.00 | 28 | 16.6 | 1.0 | 9 | 50 | 5.2 | 14.0 |
| 8849 | 1 | 1 | 37 | 22.41 | 2 | 2 | 130.0 | 86 | 1 | 3.55 | 2.21 | 1.54 | 1.76 | 29.00 | 19 | 7.0 | 1.0 | 49 | 71 | 4.2 | 12.8 |
| 357 | 2 | 1 | 79 | 24 | 2 | 2 | 110 | 70 |  | 2.1 | 1.27 | 1.26 | 1.53 | 37 | 30 | 11 | 3.5 | 11 | 87 | 6 | 11 |
| 733 | 2 | 1 | 22 | 18.7 | 1 | 1 | 95 | 60 |  | 1 | 1.47 | 1.61 | 3.25 | 23 | 20 | 9 | 1.3 | 37 | 95 | 6 | 12 |
| 743 | 2 | 2 | 55 | 19.4 | 2 | 2 | 120 | 80 |  | 6.8 | 0.96 | 1.2 | 2.62 | 14 | 16 | 8.8 | 2.4 | 58 | 49 | 5 | 15 |
| 746 | 2 | 1 | 27 | 21.5 | 2 | 2 | 110 | 70 |  | 2.8 | 3.99 | 1.48 | 2.89 | 14 | 27 | 13 | 3.8 | 39 | 57 | 4 | 15 |
| 760 | 2 | 2 | 41 | 24 | 2 | 2 | 110 | 70 |  | 2.3 | 1.36 | 1.57 | 2.03 | 18 | 38 | 13 | 3.8 | 33 | 41 | 5 | 14 |
| 774 | 2 | 1 | 56 | 24 | 1 | 2 | 120 | 80 |  | 4.6 | 1.35 | 1.47 | 2.46 | 31 | 17 | 14 | 3.2 | 23 | 52 | 5 | 16 |
| 851 | 2 | 1 | 24 | 23.4 | 1 | 2 | 110 | 70 |  | 4.4 | 1.67 | 1.77 | 3.49 | 17 | 19 | 9.6 | 1.3 | 48 | 61 | 4 | 15 |
| 856 | 2 | 1 | 37 | 29 | 1 | 2 | 120 | 80 |  | 2.4 | 2.84 | 1.1 | 2.33 | 7 | 36 | 4.7 | 1.5 | 32 | 45 | 5 | 11 |
| 868 | 2 | 1 | 47 | 26 | 2 | 2 | 110 | 70 |  | 3.9 | 1.6 | 1.5 | 2.83 | 69 | 22 | 16 | 1.1 | 44 | 90 | 6 | 14 |
| 872 | 2 | 1 | 28 | 29 | 2 | 2 | 120 | 80 |  | 3.9 | 1.48 | 1.81 | 1.66 | 18 | 21 | 3.5 | 1.7 | 20 | 81 | 4 | 15 |
| 873 | 2 | 2 | 34 | 18 | 2 | 2 | 90 | 60 |  | 4.3 | 1.38 | 1.63 | 3.62 | 11 | 26 | 11 | 2.3 | 16 | 113 | 4 | 15 |
| 874 | 2 | 2 | 55 | 23.5 | 2 | 2 | 110 | 70 |  | 3.9 | 0.63 | 1.76 | 3.32 | 30 | 18 | 8.1 | 2.2 | 47 | 106 | 6 | 15 |
| 890 | 2 | 1 | 52 | 23.9 | 1 | 1 | 142 | 90 |  | 3.7 | 3.85 | 1.13 | 2.07 | 22 | 13 | 8.6 | 1.1 | 77 | 90 | 5 | 14 |
| 905 | 2 | 1 | 59 | 21.5 | 2 | 2 | 112 | 70 |  | 6.3 | 1.57 | 1.09 | 4.31 | 16 | 36 | 12 | 2.5 | 42 | 100 | 5 | 15 |
| 906 | 2 | 1 | 52 | 23.7 | 2 | 2 | 140 | 85 |  | 4.9 | 1.48 | 1.21 | 2.13 | 36 | 14 | 11 | 3.8 | 22 | 42 | 5 | 15 |
| 918 | 2 | 1 | 58 | 22.5 | 1 | 2 | 130 | 80 |  | 3.2 | 0.97 | 1.65 | 2.81 | 12 | 28 | 10 | 3.1 | 15 | 122 | 5 | 13 |
| 919 | 2 | 1 | 46 | 26 | 2 | 2 | 130 | 80 |  | 2 | 0.8 | 1.06 | 2.91 | 24 | 31 | 11 | 1.1 | 17 | 72 | 5 | 14 |
| 931 | 2 | 1 | 58 | 21.2 | 1 | 2 | 110 | 70 |  | 3.5 | 1.49 | 1.19 | 2.52 | 18 | 37 | 7 | 1.7 | 45 | 96 | 6 | 14 |
| 942 | 2 | 1 | 54 | 28 | 1 | 2 | 120 | 80 |  | 1.2 | 1.12 | 1.57 | 4.11 | 32 | 37 | 8.6 | 2.8 | 12 | 39 | 5 | 15 |
| 944 | 2 | 2 | 36 | 24 | 1 | 1 | 130 | 80 |  | 2.7 | 1.68 | 1.74 | 2.48 | 12 | 47 | 3.5 | 2.6 | 36 | 96 | 4 | 12 |
| 960 | 2 | 2 | 56 | 24 | 2 | 2 | 120 | 80 |  | 4.4 | 1.63 | 1.05 | 2.93 | 12 | 18 | 7.5 | 3 | 11 | 78 | 5 | 14 |
| 1211 | 2 | 1 | 68 | 31 | 2 | 2 | 140 | 90 |  | 4.9 | 1.36 | 1.61 | 1.88 | 23 | 20 | 8.9 | 1.9 | 18 | 56 | 5 | 15 |
| 1223 | 2 | 2 | 33 | 24 | 1 | 2 | 130 | 80 |  | 5.2 | 0.95 | 1.28 | 2.75 | 40 | 26 | 17 | 2.8 | 26 | 110 | 5 | 12 |
| 1638 | 2 | 1 | 50 | 23 | 1 | 1 | 112 | 80 |  | 2.7 | 1.17 | 1.63 | 3.23 | 30 | 41 | 8.6 | 1.8 | 9 | 64 | 6 | 15 |
| 1660 | 2 | 2 | 38 | 19.3 | 2 | 2 | 90 | 60 |  | 2.9 | 1.08 | 1.51 | 3.69 | 34 | 25 | 11 | 3.7 | 19 | 79 | 6 | 16 |
| 1678 | 2 | 2 | 38 | 25 | 2 | 2 | 120 | 70 |  | 1.2 | 1.39 | 1.32 | 2.77 | 22 | 8 | 5.3 | 3.8 | 41 | 66 | 5 | 16 |
| 1682 | 2 | 1 | 38 | 25 | 1 | 2 | 110 | 80 |  | 1.6 | 1.11 | 1.21 | 3.51 | 30 | 27 | 15 | 3 | 18 | 93 | 4 | 14 |
| 2154 | 2 | 2 | 36 | 19.1 | 2 | 2 | 100 | 60 |  | 5 | 1.06 | 1.37 | 1.99 | 15 | 37 | 16 | 4 | 28 | 81 | 4 | 12 |
| 2155 | 2 | 1 | 29 | 24 | 1 | 2 | 120 | 80 |  | 1.9 | 0.9 | 1.48 | 1.76 | 35 | 19 | 12 | 1.9 | 9 | 44 | 6 | 14 |
| 2159 | 2 | 2 | 40 | 20.8 | 2 | 2 | 120 | 80 |  | 5.1 | 1.08 | 1.63 | 1.61 | 17 | 33 | 17 | 2.3 | 16 | 49 | 5 | 13 |
| 2160 | 2 | 2 | 32 | 20.6 | 2 | 2 | 110 | 70 |  | 4.7 | 0.7 | 1.04 | 4.13 | 36 | 39 | 13 | 3 | 43 | 54 | 6 | 12 |
| 2162 | 2 | 2 | 36 | 23.2 | 1 | 2 | 110 | 70 |  | 3.3 | 1.66 | 1.22 | 3.05 | 12 | 16 | 16 | 3 | 7 | 99 | 5 | 14 |
| 2163 | 2 | 2 | 38 | 21.5 | 2 | 2 | 110 | 80 |  | 3.2 | 1.32 | 1.78 | 3.24 | 30 | 36 | 7.4 | 2.3 | 48 | 84 | 5 | 12 |
| 2164 | 2 | 2 | 34 | 21.5 | 2 | 2 | 100 | 60 |  | 2.8 | 0.91 | 0.88 | 3.27 | 33 | 32 | 18 | 6.1 | 37 | 75 | 4 | 15 |
| 2165 | 2 | 2 | 43 | 18.8 | 1 | 1 | 112 | 70 |  | 4.2 | 1.62 | 1.18 | 1.94 | 14 | 38 | 19 | 6.3 | 26 | 125 | 4 | 16 |
| 2170 | 2 | 1 | 31 | 19.3 | 1 | 1 | 112 | 70 |  | 3.5 | 0.92 | 1.29 | 1.82 | 7 | 34 | 17 | 2.1 | 25 | 89 | 5 | 13 |
| 2172 | 2 | 1 | 22 | 18.8 | 2 | 2 | 112 | 70 |  | 3.9 | 1.14 | 1.67 | 2.18 | 11 | 35 | 8.5 | 4 | 64 | 42 | 5 | 13 |
| 2173 | 2 | 1 | 39 | 18.5 | 1 | 1 | 120 | 80 |  | 6 | 3.23 | 1.67 | 4.01 | 12 | 11 | 14 | 1.7 | 19 | 59 | 6 | 12 |
| 2174 | 2 | 1 | 38 | 23.9 | 1 | 2 | 110 | 70 |  | 3.7 | 4.19 | 1.44 | 4.01 | 15 | 37 | 14 | 2.2 | 24 | 72 | 5 | 11 |
| 2175 | 2 | 1 | 27 | 19.6 | 2 | 2 | 90 | 60 |  | 5.9 | 1.25 | 1.06 | 4.01 | 38 | 21 | 18 | 6.3 | 14 | 47 | 5 | 14 |
| 2178 | 2 | 2 | 41 | 20.7 | 2 | 2 | 100 | 70 |  | 3.5 | 1.36 | 1.25 | 2.54 | 18 | 19 | 13 | 3.5 | 11 | 115 | 5 | 13 |
| 2180 | 2 | 1 | 59 | 22.9 | 2 |  | 120 | 80 |  | 4.1 | 1.07 | 1.17 | 4.08 | 12 | 34 | 9.9 | 2.8 | 34 | 99 | 5 | 17 |
| 2183 | 2 | 2 | 32 | 18 | 1 | 2 | 120 | 78 |  | 2.8 | 1.57 | 1.17 | 1.84 | 36 | 15 | 18 | 6.8 | 34 | 116 | 5 | 14 |
| 2185 | 2 | 2 | 41 | 23.9 | 1 | 1 | 110 | 80 |  | 1.6 | 1.46 | 1.31 | 2.27 | 38 | 24 | 8.4 | 2.4 | 39 | 57 | 4 | 12 |
| 2186 | 2 | 2 | 54 | 20.1 | 2 | 2 | 120 | 70 |  | 2.8 | 1.36 | 1.78 | 1.99 | 16 | 11 | 17 | 4.2 | 16 | 125 | 5 | 12 |
| 2187 | 2 | 1 | 46 | 26 | 2 | 2 | 120 | 80 |  | 5.5 | 0.7 | 1.25 | 4.15 | 22 | 28 | 17 | 1.7 | 41 | 48 | 4 | 14 |
| 2192 | 2 | 1 | 48 | 25 | 1 | 2 | 120 | 80 |  | 5.9 | 1.71 | 1.32 | 4.53 | 36 | 18 | 7.1 | 1.2 | 31 | 42 | 6 | 13 |
| 2199 | 2 | 1 | 24 | 22.2 | 1 | 2 | 110 | 74 |  | 3.8 | 1.63 | 1.35 | 2.78 | 10 | 21 | 15 | 2.2 | 62 | 100 | 5 | 14 |
| 2200 | 2 | 1 | 29 | 25 | 1 | 1 | 120 | 80 |  | 1.4 | 1.41 | 1.62 | 2.94 | 23 | 21 | 27 | 10 | 29 | 65 | 5 | 13 |
| 2201 | 2 | 1 | 37 | 24 | 2 | 2 | 105 | 70 |  | 1.9 | 1.29 | 1.09 | 1.4 | 30 | 31 | 22 | 8 | 28 | 77 | 5 | 11 |
| 2207 | 2 | 1 | 40 | 24 | 1 | 2 | 120 | 70 |  | 5.6 | 1.13 | 1.03 | 3.21 | 27 | 20 | 25 | 7 | 15 | 112 | 5 | 14 |
| 2217 | 2 | 2 | 51 | 24.9 | 2 | 2 | 110 | 70 |  | 5.7 | 1.69 | 1.77 | 1.43 | 38 | 9 | 3.6 | 2.7 | 14 | 97 | 5 | 12 |
| 2226 | 2 | 1 | 63 | 25 | 2 | 2 | 140 | 90 |  | 4.3 | 1.26 | 1.65 | 1.82 | 23 | 29 | 8.6 | 1.5 | 14 | 99 | 5 | 13 |
| 2237 | 2 | 1 | 45 | 24.6 | 1 | 1 | 110 | 80 |  | 5.6 | 1.05 | 1.82 | 1.33 | 35 | 23 | 14 | 2.9 | 24 | 38 | 6 | 12 |
| 2283 | 2 | 1 | 57 | 25 | 2 | 2 | 140 | 90 |  | 5.4 | 0.62 | 1.7 | 2.61 | 11 | 19 | 5 | 4.1 | 41 | 53 | 5 | 14 |
| 2297 | 2 | 1 | 36 | 26 | 2 | 2 | 110 | 70 |  | 1.2 | 1.26 | 1.47 | 3.98 | 39 | 27 | 12 | 3.4 | 15 | 121 | 5 | 16 |
| 2304 | 2 | 1 | 40 | 21.2 | 2 | 2 | 110 | 80 |  | 4.3 | 0.91 | 1.14 | 2.93 | 17 | 34 | 12 | 2.1 | 50 | 118 | 6 | 14 |
| 2306 | 2 | 2 | 30 | 25 | 2 | 2 | 120 | 80 |  | 6.3 | 2.45 | 1.84 | 2.66 | 9 | 24 | 9.1 | 1.4 | 48 | 101 | 5 | 13 |
| 2315 | 2 | 1 | 62 | 23.1 | 2 | 2 | 120 | 80 |  | 6.3 | 0.76 | 1.82 | 2.96 | 37 | 13 | 6 | 3.3 | 24 | 73 | 6 | 12 |
| 2331 | 2 | 1 | 41 | 22.5 | 2 | 2 | 100 | 70 |  | 5.2 | 0.68 | 1.58 | 1.39 | 24 | 33 | 6 | 2.7 | 19 | 44 | 6 | 12 |
| 2354 | 2 | 1 | 50 | 26 | 2 | 2 | 110 | 70 |  | 1.7 | 3.52 | 1.64 | 2.51 | 32 | 20 | 12 | 3.3 | 10 | 62 | 5 | 14 |
| 2361 | 2 | 1 | 62 | 22.1 | 2 | 2 | 120 | 80 |  | 5.6 | 0.71 | 1.36 | 1.6 | 14 | 22 | 7 | 4 | 25 | 114 | 5 | 14 |
| 2449 | 2 | 1 | 32 | 21.5 | 2 | 2 | 110 | 70 |  | 1.6 | 1.56 | 1.84 | 1.67 | 18 | 23 | 18 | 1.3 | 16 | 53 | 6 | 13 |
| 2451 | 2 | 2 | 44 | 21.2 | 2 | 2 | 100 | 60 |  | 2.1 | 1.42 | 1.31 | 2.88 | 16 | 16 | 4.8 | 4 | 35 | 74 | 4 | 13 |
| 2458 | 2 | 2 | 43 | 22.9 | 2 | 2 | 140 | 100 |  | 4.9 | 1.57 | 1.81 | 2.73 | 18 | 45 | 5.3 | 4.4 | 45 | 64 | 5 | 16 |
| 2465 | 2 | 1 | 34 | 20.4 | 1 | 2 | 130 | 90 |  | 5.7 | 0.84 | 1.76 | 1.85 | 12 | 33 | 14 | 3.9 | 37 | 47 | 4 | 11 |
| 2469 | 2 | 1 | 58 | 20.3 | 1 | 2 | 110 | 70 |  | 3.3 | 0.72 | 1.83 | 3.08 | 22 | 16 | 16 | 1.7 | 81 | 123 | 5 | 12 |
| 2472 | 2 | 2 | 48 | 21.7 | 1 | 2 | 110 | 70 |  | 7.2 | 0.6 | 1.64 | 5.22 | 32 | 34 | 15 | 1.1 | 32 | 65 | 4 | 13 |
| 2475 | 2 | 1 | 53 | 24 | 2 | 2 | 120 | 80 |  | 5.2 | 1.26 | 1.24 | 2.36 | 17 | 38 | 17 | 3.7 | 20 | 82 | 5 | 13 |
| 2498 | 2 | 1 | 65 | 21.8 | 1 | 1 | 100 | 60 |  | 6.1 | 0.88 | 1.53 | 4.19 | 15 | 27 | 12 | 1.5 | 19 | 59 | 4 | 14 |
| 2501 | 2 | 1 | 41 | 22.9 | 2 | 2 | 100 | 70 |  | 1.3 | 0.99 | 1.24 | 1.64 | 10 | 34 | 3.9 | 2 | 13 | 46 | 5 | 12 |
| 2503 | 2 | 1 | 49 | 23.4 | 2 | 2 | 140 | 90 |  | 5.7 | 1.19 | 1.61 | 2.88 | 32 | 31 | 15 | 4.1 | 36 | 64 | 5 | 14 |
| 2508 | 2 | 1 | 33 | 25 | 2 | 2 | 112 | 80 |  | 5.4 | 0.61 | 1.57 | 2.95 | 28 | 30 | 8.9 | 2.9 | ## | 74 | 4 | 15 |
| 2534 | 2 | 1 | 23 | 25 | 2 | 2 | 105 | 70 |  | 1.5 | 0.92 | 1.28 | 3.35 | 13 | 38 | 17 | 1.9 | 22 | 91 | 6 | 14 |
| 2536 | 2 | 2 | 72 | 20.7 | 1 | 1 | 120 | 70 |  | 6.4 | 1.48 | 1.22 | 1.7 | 29 | 11 | 15 | 1 | ## | 121 | 6 | 12 |
| 2546 | 2 | 2 | 41 | 22.3 | 1 | 2 | 100 | 70 |  | 3.9 | 1.45 | 1.78 | 2.05 | 32 | 49 | 20 | 3.3 | 43 | 96 | 5 | 15 |
| 2547 | 2 | 1 | 60 | 24 | 2 | 2 | 120 | 70 |  | 4 | 0.61 | 1.84 | 3.53 | 36 | 38 | 20 | 4.2 | 44 | 55 | 5 | 12 |
| 2548 | 2 | 1 | 48 | 24 | 1 | 1 | 120 | 80 |  | 3.8 | 1.06 | 1.37 | 4.01 | 12 | 30 | 14 | 3.6 | 12 | 80 | 5 | 13 |
| 2552 | 2 | 1 | 35 | 24 | 2 | 2 | 100 | 70 |  | 6.1 | 2.63 | 1.62 | 3.48 | 26 | 29 | 13 | 3.5 | 39 | 61 | 4 | 14 |
| 2557 | 2 | 1 | 53 | 22.4 | 2 | 2 | 160 | 80 |  | 3.8 | 0.79 | 1.84 | 3.93 | 40 | 21 | 12 | 1.1 | 25 | 98 | 4 | 16 |
| 2561 | 2 | 1 | 55 | 24 | 1 | 2 | 110 | 80 |  | 3 | 1.55 | 1.63 | 2.75 | 13 | 40 | 18 | 7 | 39 | 115 | 5 | 15 |
| 2562 | 2 | 2 | 43 | 21.1 | 2 | 2 | 100 | 70 |  | 3.3 | 1.27 | 1.21 | 3.13 | 27 | 22 | 17 | 2.6 | 32 | 84 | 5 | 15 |
| 2567 | 2 | 2 | 43 | 19.8 | 2 | 2 | 120 | 70 |  | 1.8 | 1.27 | 0.91 | 3.74 | 29 | 15 | 9 | 2.3 | 15 | 56 | 4 | 12 |
| 2919 | 2 | 1 | 54 | 24 | 1 | 2 | 120 | 80 |  | 4.7 | 1.2 | 1.13 | 3.16 | 37 | 24 | 12 | 4.4 | 12 | 53 | 5 | 14 |
| 2921 | 2 | 1 | 72 | 25 | 1 | 1 | 120 | 80 |  | 2.6 | 1 | 1.47 | 3.12 | 20 | 20 | 15 | 2.8 | 49 | 46 | 4 | 16 |
| 2922 | 2 | 2 | 42 | 21.8 | 2 | 2 | 100 | 70 |  | 5.5 | 1.08 | 1.14 | 3.57 | 29 | 12 | 18 | 3.9 | 53 | 85 | 4 | 13 |
| 2927 | 2 | 2 | 42 | 22.7 | 2 | 2 | 100 | 70 |  | 4.8 | 0.78 | 1.37 | 3.36 | 29 | 23 | 5 | 2.7 | 22 | 74 | 4 | 11 |
| 2929 | 2 | 2 | 44 | 22.7 | 2 | 2 | 110 | 80 |  | 6 | 1.32 | 1.35 | 2.21 | 20 | 32 | 13 | 1.6 | 29 | 47 | 5 | 13 |
| 2933 | 2 | 2 | 45 | 21.5 | 2 | 2 | 100 | 70 |  | 1.7 | 1.57 | 1.43 | 3.58 | 32 | 38 | 8 | 1 | 31 | 95 | 5 | 16 |
| 2945 | 2 | 1 | 59 | 25 | 2 | 2 | 120 | 80 |  | 4.9 | 1.62 | 1.59 | 1.55 | 15 | 32 | 16 | 2.8 | 33 | 58 | 4 | 14 |
| 2963 | 2 | 2 | 47 | 23 | 2 | 2 | 110 | 70 |  | 5.4 | 0.62 | 1.59 | 3.06 | 28 | 18 | 13 | 3.1 | 36 | 82 | 5 | 14 |
| 2990 | 2 | 2 | 57 | 23.1 | 2 | 2 | 110 | 70 |  | 6.3 | 2.37 | 1.18 | 4.3 | 30 | 29 | 14 | 3.1 | 33 | 120 | 5 | 12 |
| 2996 | 2 | 1 | 54 | 24.2 | 2 | 2 | 130 | 90 |  | 5.9 | 0.68 | 1.22 | 2.1 | 35 | 34 | 17 | 1.9 | 88 | 86 | 7 | 12 |
| 3004 | 2 | 2 | 41 | 20.8 | 2 | 2 | 110 | 70 |  | 4.3 | 1.57 | 1.55 | 3.26 | 30 | 8 | 4.2 | 1.3 | 11 | 41 | 5 | 15 |
| 3009 | 2 | 1 | 79 | 19.6 |  | 2 | 120 | 80 |  | 3.4 | 1.11 | 0.97 | 2.73 | 12 | 35 | 12 | 3.4 | 41 | 95 | 7 | 21 |
| 3011 | 2 | 2 | 81 | 17 | 2 | 2 | 120 | 70 |  | 1.1 | 0.72 | 1.13 | 1.36 | 17 | 30 | 11 | 2.4 | 19 | 57 | 5 | 11 |
| 3012 | 2 | 2 | 55 | 23.1 | 2 | 2 | 120 | 80 |  | 4.7 | 2.03 | 1.65 | 3.78 | 34 | 31 | 16 | 1.4 | 53 | 81 | 6 | 16 |
| 3020 | 2 | 2 | 49 | 20 | 2 | 2 | 100 | 70 |  | 5.6 | 1.37 | 1.57 | 3.52 | 12 | 25 | 7.3 | 1.4 | 35 | 85 | 5 | 13 |
| 3021 | 2 | 1 | 30 | 16 | 2 | 2 | 120 | 80 |  | 4.1 | 1.53 | 1.83 | 3.5 | 27 | 26 | 5.1 | 3.1 | 29 | 75 | 6 | 13 |
| 3022 | 2 | 2 | 42 | 21.7 | 2 | 2 | 100 | 65 |  | 2 | 1.65 | 1.27 | 3.55 | 17 | 15 | 4.3 | 3.2 | 22 | 45 | 5 | 11 |
| 3028 | 2 | 2 | 57 | 18.1 | 1 | 2 | 100 | 70 |  | 7.1 | 2.16 | 1.49 | 5.32 | 35 | 35 | 12 | 3.1 | 43 | 47 | 5 | 18 |
| 3046 | 2 | 2 | 62 | 22.1 | 1 | 2 | 100 | 60 |  | 5.7 | 1.2 | 1.4 | 2.84 | 26 | 39 | 9 | 3.9 | 43 | 100 | 6 | 11 |
| 3050 | 2 | 2 | 48 | 20.6 | 2 | 2 | 110 | 70 |  | 4.5 | 0.71 | 1.37 | 1.67 | 30 | 23 | 14 | 3.4 | 21 | 111 | 6 | 14 |
| 3051 | 2 | 2 | 47 | 19.6 | 2 | 2 | 110 | 70 |  | 6.1 | 1.08 | 1.25 | 3.03 | 8 | 17 | 13 | 1.4 | 16 | 122 | 6 | 11 |
| 3439 | 2 | 2 | 46 | 20 | 2 | 2 | 100 | 70 |  | 3.7 | 1.36 | 1.32 | 3.98 | 37 | 15 | 11 | 1.7 | 47 | 72 | 4 | 14 |
| 3443 | 2 | 2 | 49 | 19.8 | 2 | 2 | 106 | 70 |  | 6 | 1.32 | 1.55 | 1.51 | 29 | 19 | 15 | 2.3 | 22 | 109 | 6 | 15 |
| 3607 | 2 | 1 | 70 | 24 | 2 | 2 | 140 | 80 |  | 3.7 | 0.79 | 0.98 | 3.07 | 10 | 9 | 20 | 1.7 | 41 | 124 | 6 | 12 |
| 3609 | 2 | 2 | 43 | 21.3 | 2 | 2 | 100 | 70 |  | 4.1 | 0.93 | 1.33 | 3.57 | 7 | 19 | 13 | 4.1 | 33 | 43 | 4 | 13 |
| 3614 | 2 | 2 | 57 | 23.1 | 2 | 2 | 120 | 84 |  | 1.3 | 1.05 | 1.81 | 1.3 | 17 | 30 | 15 | 4.3 | 24 | 102 | 5 | 14 |
| 3622 | 2 | 2 | 55 | 23.9 | 2 | 2 | 130 | 80 |  | 5.3 | 1.4 | 1.39 | 2.18 | 36 | 23 | 18 | 1.4 | 41 | 125 | 6 | 15 |
| 3629 | 2 | 2 | 54 | 22.1 | 1 | 1 | 110 | 80 |  | 2.5 | 0.95 | 1.4 | 1.79 | 12 | 27 | 16 | 3.8 | 50 | 100 | 6 | 13 |
| 3631 | 2 | 2 | 47 | 22.4 | 1 | 2 | 120 | 80 |  | 6.3 | 1.55 | 1.02 | 4.33 | 26 | 26 | 10 | 2.2 | 30 | 94 | 6 | 13 |
| 3635 | 2 | 2 | 41 | 22.3 | 2 | 2 | 120 | 80 |  | 5.1 | 1.33 | 1.51 | 3.91 | 11 | 15 | 4.6 | 3.4 | 33 | 83 | 6 | 15 |
| 3650 | 2 | 1 | 79 | 24 | 2 | 2 | 130 | 80 |  | 2.1 | 1.16 | 1.03 | 2.27 | 17 | 17 | 11 | 1.8 | 35 | 56 | 4 | 15 |
| 3653 | 2 | 2 | 49 | 19.3 | 2 | 2 | 100 | 60 |  | 1.9 | 1.36 | 1.34 | 3.12 | 31 | 32 | 6.5 | 4.2 | 49 | 40 | 6 | 14 |
| 3738 | 2 | 2 | 45 | 21.1 | 2 | 2 | 120 | 80 |  | 4.9 | 0.66 | 1.52 | 1.55 | 13 | 9 | 14 | 2.2 | 49 | 66 | 4 | 11 |
| 4011 | 2 | 2 | 58 | 22.3 | 2 | 2 | 110 | 70 |  | 1.2 | 1.48 | 0.96 | 2.28 | 14 | 39 | 6.6 | 2.9 | 33 | 113 | 6 | 12 |
| 4015 | 2 | 2 | 56 | 19.6 | 2 | 2 | 100 | 64 |  | 3.4 | 1.32 | 0.94 | 1.4 | 32 | 17 | 17 | 3.4 | 45 | 100 | 6 | 11 |
| 4017 | 2 | 2 | 58 | 19.2 | 2 | 2 | 100 | 70 |  | 3.6 | 0.97 | 1.12 | 1.66 | 16 | 38 | 9.8 | 4.1 | 23 | 119 | 4 | 14 |
| 4018 | 2 | 2 | 57 | 22.2 | 1 | 1 | 110 | 70 |  | 5.3 | 1.12 | 1.31 | 3.62 | 34 | 19 | 16 | 1.6 | 7 | 54 | 5 | 15 |
| 4020 | 2 | 2 | 54 | 22.1 | 2 | 2 | 140 | 90 |  | 5.4 | 1.84 | 1.14 | 1.72 | 25 | 32 | 4.2 | 3 | 74 | 88 | 6 | 17 |
| 4022 | 2 | 2 | 55 | 20.8 | 1 | 2 | 130 | 80 |  | 5.8 | 0.68 | 1.05 | 1.31 | 8 | 38 | 12 | 3.9 | 16 | 92 | 5 | 16 |
| 4041 | 2 | 1 | 31 | 25 | 2 | 2 | 100 | 65 |  | 3.8 | 2.01 | 1.26 | 3.87 | 12 | 37 | 11 | 3.1 | 45 | 43 | 6 | 14 |
| 4051 | 2 | 1 | 58 | 26 | 2 | 2 | 130 | 80 |  | 2.4 | 3.22 | 0.86 | 3.94 | 16 | 26 | 14 | 3.9 | 35 | 71 | 6 | 13 |
| 4055 | 2 | 1 | 51 | 22.3 | 2 | 2 | 110 | 76 |  | 4.8 | 0.68 | 1.12 | 2.6 | 19 | 20 | 14 | 2.7 | 41 | 54 | 4 | 14 |
| 4070 | 2 | 1 | 59 | 19.5 | 2 | 2 | 120 | 76 |  | 3.8 | 1.28 | 1.77 | 1.5 | 15 | 40 | 4.8 | 3.6 | 17 | 117 | 5 | 12 |
| 4073 | 2 | 2 | 53 | 27 | 2 | 2 | 120 | 84 |  | 6.6 | 1.75 | 1.45 | 5.06 | 27 | 15 | 11 | 3.2 | 21 | 107 | 4 | 13 |
| 4074 | 2 | 1 | 59 | 25 | 2 | 2 | 120 | 86 |  | 2.8 | 2.6 | 1.28 | 2.01 | 11 | 15 | 9 | 1.4 | 41 | 108 | 5 | 12 |
| 4083 | 2 | 2 | 36 | 24 | 2 | 2 | 100 | 70 |  | 2.4 | 1.29 | 1.09 | 4.05 | 17 | 37 | 18 | 7.4 | 47 | 97 | 5 | 14 |
| 4093 | 2 | 2 | 50 | 18.6 | 2 | 2 | 140 | 90 |  | 5.4 | 1.41 | 1.04 | 3.03 | 19 | 22 | 17 | 1 | 21 | 89 | 6 | 11 |
| 4095 | 2 | 1 | 59 | 25 | 2 | 2 | 130 | 80 |  | 4.8 | 0.94 | 1.47 | 3.74 | 14 | 23 | 14 | 4.2 | 50 | 49 | 6 | 15 |
| 4102 | 2 | 1 | 56 | 22.2 | 2 | 2 | 130 | 86 |  | 5.3 | 1.06 | 1.6 | 3.9 | 10 | 14 | 14 | 4.1 | 61 | 71 | 5 | 15 |
| 4107 | 2 | 1 | 25 | 25 | 2 | 2 | 100 | 70 |  | 4.8 | 2.17 | 1.39 | 2.24 | 9 | 24 | 12 | 2.6 | 26 | 68 | 5 | 15 |
| 4113 | 2 | 2 | 55 | 25 | 1 | 2 | 140 | 90 |  | 5.3 | 0.68 | 1.84 | 3.1 | 20 | 23 | 12 | 3.3 | 41 | 55 | 5 | 17 |
| 4116 | 2 | 1 | 58 | 24 | 1 | 2 | 120 | 90 |  | 2.9 | 0.67 | 1.21 | 1.51 | 30 | 36 | 16 | 3.4 | 8 | 45 | 5 | 13 |
| 4119 | 2 | 2 | 38 | 25 | 2 | 2 | 100 | 70 |  | 3.3 | 1.38 | 1.4 | 2.42 | 13 | 35 | 8.6 | 1.6 | 42 | 108 | 4 | 14 |
| 4138 | 2 | 2 | 42 | 24 | 2 | 2 | 100 | 64 |  | 1.2 | 1.58 | 1.72 | 3.15 | 18 | 20 | 15 | 3.2 | 23 | 48 | 5 | 12 |
| 4150 | 2 | 1 | 46 | 24 | 2 | 2 | 120 | 80 |  | 4.4 | 1.15 | 1.52 | 3.94 | 11 | 33 | 8.2 | 1.8 | 9 | 66 | 4 | 13 |
| 4151 | 2 | 1 | 47 | 21.6 | 2 | 2 | 100 | 65 |  | 1 | 2.58 | 1.23 | 3.46 | 15 | 25 | 6.1 | 3 | 48 | 51 | 6 | 12 |
| 4153 | 2 | 1 | 52 | 24 | 2 | 2 | 110 | 70 |  | 1.5 | 1.84 | 1.3 | 4.03 | 21 | 11 | 7 | 4.1 | 38 | 81 | 4 | 16 |
| 4159 | 2 | 2 | 28 | 25 | 2 | 2 | 110 | 70 |  | 5.6 | 2.28 | 1.42 | 3.93 | 18 | 37 | 8.4 | 4.1 | 32 | 84 | 5 | 13 |
| 4164 | 2 | 2 | 35 | 25 | 2 | 2 | 100 | 64 |  | 5.9 | 0.61 | 1.51 | 4.2 | 12 | 28 | 10 | 1.7 | 42 | 116 | 5 | 15 |
| 4180 | 2 | 2 | 33 | 24 | 2 | 2 | 120 | 80 |  | 3 | 1.34 | 1.26 | 3.25 | 37 | 28 | 4.4 | 2.8 | 26 | 70 | 5 | 15 |
| 4182 | 2 | 2 | 22 | 24 | 2 | 2 | 100 | 60 |  | 4.3 | 0.7 | 1.73 | 1.58 | 19 | 27 | 12 | 2.1 | 18 | 62 | 6 | 15 |
| 4186 | 2 | 1 | 53 | 25 | 2 | 2 | 120 | 90 |  | 2.4 | 0.8 | 1.19 | 2.73 | 11 | 27 | 17 | 1.5 | 47 | 75 | 5 | 12 |
| 4187 | 2 | 2 | 58 | 25 | 2 | 2 | 130 | 70 |  | 5.7 | 0.63 | 0.98 | 2 | 23 | 15 | 9.5 | 2.4 | 39 | 124 | 5 | 15 |
| 4188 | 2 | 2 | 44 | 27 | 2 | 2 | 110 | 70 |  | 3.2 | 1.32 | 1.2 | 1.42 | 29 | 12 | 11 | 3.7 | 29 | 39 | 4 | 14 |
| 4194 | 2 | 2 | 29 | 27 | 2 | 2 | 100 | 70 |  | 4.7 | 0.79 | 1.08 | 3.81 | 27 | 14 | 3.6 | 3.6 | 46 | 115 | 5 | 12 |
| 4198 | 2 | 2 | 55 | 21.5 | 2 | 2 | 100 | 70 |  | 2.9 | 1.08 | 1.58 | 3.6 | 37 | 23 | 16 | 1.2 | 45 | 42 | 6 | 11 |
| 4389 | 2 | 1 | 37 | 28 | 2 | 2 | 110 | 70 |  | 5.9 | 1.53 | 1.16 | 4.26 | 12 | 21 | 6.3 | 2.4 | 41 | 50 | 5 | 16 |
| 4397 | 2 | 1 | 59 | 29 | 2 | 2 | 140 | 90 |  | 5 | 0.65 | 1.42 | 2.77 | 11 | 32 | 16 | 2.1 | 23 | 102 | 5 | 16 |
| 4401 | 2 | 2 | 55 | 24 | 2 | 2 | 120 | 80 |  | 5.2 | 2.12 | 1.07 | 1.33 | 25 | 34 | 13 | 1.7 | 10 | 106 | 6 | 14 |
| 4402 | 2 | 1 | 46 | 24 | 1 | 2 | 120 | 80 |  | 6.2 | 1.86 | 1.46 | 2.69 | 39 | 12 | 21 | 3.4 | 54 | 96 | 5 | 12 |
| 4403 | 2 | 1 | 42 | 25 | 1 | 2 | 130 | 90 |  | 5.6 | 2.19 | 1.65 | 3.54 | 19 | 32 | 23 | 6.2 | 10 | 62 | 5 | 11 |
| 4404 | 2 | 1 | 42 | 20.3 | 2 | 2 | 120 | 80 |  | 3.2 | 0.62 | 1.21 | 2.36 | 21 | 46 | 17 | 8.8 | ## | 112 | 6 | 15 |
| 4408 | 2 | 2 | 26 | 25 | 2 | 2 | 120 | 80 |  | 4 | 1.27 | 1.49 | 2.17 | 38 | 16 | 7.1 | 4.2 | 40 | 58 | 6 | 15 |
| 4417 | 2 | 2 | 32 | 24 | 2 | 2 | 120 | 70 |  | 1 | 0.68 | 1.2 | 2.37 | 15 | 23 | 4.9 | 3.9 | 52 | 56 | 5 | 14 |
| 4427 | 2 | 2 | 34 | 25 | 1 | 2 | 120 | 80 |  | 5.3 | 1.18 | 1.17 | 3.52 | 9 | 31 | 13 | 1.9 | 37 | 72 | 5 | 15 |
| 4432 | 2 | 1 | 46 | 22 | 2 | 2 | 110 | 70 |  | 2.3 | 1.5 | 1.65 | 2.37 | 15 | 11 | 4.6 | 3 | 53 | 121 | 5 | 12 |
| 4446 | 2 | 2 | 54 | 20.7 | 1 | 2 | 125 | 80 |  | 3.7 | 1.21 | 1.24 | 1.42 | 28 | 16 | 11 | 1.1 | 16 | 85 | 5 | 13 |
| 4453 | 2 | 2 | 44 | 27 | 2 | 2 | 110 | 70 |  | 3.4 | 0.95 | 0.97 | 2.88 | 40 | 35 | 17 | 2.1 | 13 | 116 | 5 | 11 |
| 4491 | 2 | 1 | 59 | 25 | 2 | 2 | 120 | 80 |  | 2.7 | 1.11 | 1.09 | 1.92 | 19 | 28 | 17 | 3.3 | 17 | 55 | 5 | 11 |
| 4492 | 2 | 1 | 57 | 24 | 2 | 2 | 120 | 80 |  | 5.5 | 0.69 | 0.96 | 2.43 | 21 | 26 | 12 | 3.6 | 72 | 64 | 4 | 15 |
| 4494 | 2 | 1 | 58 | 28 | 1 | 1 | 140 | 90 |  | 6.2 | 1.54 | 1.5 | 4.33 | 14 | 25 | 14 | 1.8 | 64 | 79 | 4 | 14 |
| 4501 | 2 | 1 | 49 | 19.6 | 2 | 2 | 110 | 70 |  | 2.3 | 1.58 | 1.37 | 2.37 | 38 | 39 | 4.3 | 2.5 | 12 | 49 | 4 | 16 |
| 4503 | 2 | 1 | 58 | 23.3 | 2 | 2 | 120 | 80 |  | 7.4 | 1.93 | 0.87 | 5.07 | 14 | 31 | 7.1 | 2.4 | 44 | 109 | 6 | 13 |
| 4508 | 2 | 2 | 45 | 24.2 | 1 | 2 | 130 | 90 |  | 4.6 | 1.33 | 1.45 | 3.07 | 13 | 21 | 4.5 | 3.2 | 21 | 45 | 5 | 15 |
| 4509 | 2 | 1 | 50 | 21.3 | 2 | 2 | 110 | 70 |  | 5.4 | 0.95 | 1.78 | 3.94 | 7 | 10 | 7.7 | 3.5 | 46 | 88 | 5 | 12 |
| 4510 | 2 | 2 | 50 | 21.3 | 2 | 2 | 110 | 70 |  | 6.9 | 1.58 | 1.49 | 4.5 | 10 | 30 | 15 | 4.1 | 25 | 79 | 5 | 13 |
| 4515 | 2 | 2 | 32 | 26 | 1 | 1 | 118 | 70 |  | 4.5 | 1.64 | 1.17 | 2.71 | 38 | 20 | 8.2 | 2.2 | 20 | 65 | 6 | 11 |
| 4517 | 2 | 2 | 51 | 22.8 | 1 | 1 | 110 | 70 |  | 7.1 | 1.88 | 1.57 | 4.85 | 18 | 41 | 18 | 3 | 40 | 96 | 6 | 13 |
| 4518 | 2 | 2 | 51 | 19.9 | 2 | 2 | 108 | 70 |  | 6.5 | 0.93 | 2.15 | 4.15 | 18 | 8 | 31 | 6.4 | 28 | 45 | 4 | 13 |
| 4520 | 2 | 2 | 54 | 23.9 | 2 | 2 | 135 | 90 |  | 5.9 | 1.76 | 1.72 | 3.46 | 37 | 13 | 15 | 3.3 | 40 | 93 | 5 | 16 |
| 4522 | 2 | 2 | 50 | 19.2 | 2 | 2 | 90 | 60 |  | 5.5 | 0.8 | 1.24 | 2.57 | 30 | 9 | 15 | 2.9 | 13 | 98 | 5 | 15 |
| 4523 | 2 | 2 | 57 | 22.4 | 1 | 1 | 100 | 70 |  | 6.3 | 0.72 | 0.99 | 3.02 | 15 | 22 | 11 | 1.8 | 8 | 104 | 4 | 13 |
| 4524 | 2 | 1 | 61 | 25 | 2 | 2 | 140 | 100 |  | 3.4 | 2.45 | 1.73 | 2.17 | 33 | 57 | 8.6 | 1.2 | 73 | 97 | 5 | 17 |
| 4538 | 2 | 2 | 36 | 24 | 1 | 1 | 120 | 80 |  | 5.7 | 1.26 | 1.1 | 2.3 | 16 | 22 | 5.1 | 1.5 | 34 | 41 | 6 | 15 |
| 4542 | 2 | 1 | 28 | 25 | 1 | 2 | 110 | 80 |  | 5.9 | 1.16 | 1.11 | 3.65 | 20 | 9 | 6.4 | 3.2 | 34 | 56 | 4 | 14 |
| 4543 | 2 | 1 | 42 | 19.1 | 1 | 2 | 105 | 70 |  | 1.5 | 1.69 | 1.11 | 3.61 | 25 | 11 | 15 | 3.1 | 44 | 84 | 5 | 16 |
| 4549 | 2 | 1 | 32 | 24 | 2 | 2 | 120 | 80 |  | 5.5 | 1.08 | 1.49 | 3.79 | 15 | 15 | 18 | 1.5 | 23 | 120 | 6 | 13 |
| 4560 | 2 | 2 | 30 | 24 | 2 | 2 | 110 | 70 |  | 4.6 | 1.69 | 1.67 | 2.02 | 41 | 29 | 22 | 8.8 | 22 | 66 | 4 | 15 |
| 4564 | 2 | 1 | 46 | 23.5 | 1 | 2 | 120 | 80 |  | 6.7 | 3.17 | 1.53 | 4.66 | 12 | 24 | 19 | 4.2 | 52 | 44 | 4 | 16 |
| 4566 | 2 | 1 | 52 | 19.3 | 1 | 2 | 120 | 80 |  | 5.3 | 1.18 | 1.28 | 3.86 | 10 | 13 | 11 | 1.4 | 49 | 56 | 5 | 15 |
| 4569 | 2 | 2 | 57 | 21.5 | 2 | 2 | 110 | 70 |  | 1.7 | 1.14 | 1.37 | 2.24 | 13 | 9 | 13 | 3.9 | 43 | 52 | 6 | 12 |
| 4591 | 2 | 1 | 50 | 23.1 | 2 | 2 | 120 | 80 |  | 5.3 | 1.65 | 1.3 | 1.66 | 29 | 36 | 9.5 | 3.4 | 47 | 117 | 5 | 15 |
| 4596 | 2 | 2 | 54 | 25 | 2 | 2 | 110 | 70 |  | 2.9 | 1.07 | 1.74 | 3.1 | 18 | 28 | 12 | 1.4 | 48 | 53 | 6 | 16 |
| 4597 | 2 | 1 | 28 | 26 | 1 | 1 | 130 | 80 |  | 1.4 | 1.68 | 1.39 | 2.94 | 19 | 47 | 50 | 10 | 34 | 71 | 5 | 13 |
| 4603 | 2 | 2 | 23 | 24 | 1 | 1 | 90 | 60 |  | 5.9 | 1.03 | 1.04 | 3.15 | 31 | 21 | 17 | 1.8 | 25 | 100 | 5 | 13 |
| 4615 | 2 | 1 | 27 | 26 | 1 | 1 | 100 | 70 |  | 2.1 | 0.93 | 1.35 | 1.41 | 11 | 21 | 6.5 | 2.4 | 47 | 98 | 4 | 11 |
| 4619 | 2 | 1 | 43 | 18.4 | 2 | 2 | 120 | 84 |  | 4.5 | 1.25 | 1.39 | 2.75 | 17 | 14 | 4.2 | 1.2 | 47 | 80 | 5 | 11 |
| 4624 | 2 | 2 | 73 | 18.7 | 1 | 2 | 140 | 90 |  | 6.5 | 1.55 | 1.14 | 2.53 | 11 | 31 | 11 | 2.4 | 14 | 108 | 6 | 14 |
| 4628 | 2 | 1 | 55 | 25 | 2 | 2 | 100 | 70 |  | 3.8 | 0.82 | 1.45 | 1.82 | 37 | 14 | 17 | 7.4 | 12 | 78 | 6 | 16 |
| 4641 | 2 | 2 | 56 | 24 | 2 | 2 | 140 | 85 |  | 3.3 | 1.58 | 1.41 | 3.87 | 8 | 39 | 9 | 1.6 | 47 | 83 | 4 | 12 |
| 4642 | 2 | 1 | 56 | 22.5 | 1 | 2 | 110 | 80 |  | 5.2 | 2.06 | 0.91 | 2.49 | 26 | 20 | 11 | 2.1 | 39 | 123 | 4 | 12 |
| 4667 | 2 | 1 | 52 | 20.7 | 2 | 2 | 130 | 80 |  | 2.1 | 0.9 | 1.48 | 2.56 | 39 | 30 | 3.6 | 1.3 | 27 | 44 | 5 | 13 |
| 4673 | 2 | 1 | 25 | 25 | 1 | 2 | 110 | 70 |  | 4.9 | 1.53 | 1.02 | 3.99 | 26 | 16 | 10 | 2.9 | 13 | 55 | 4 | 14 |
| 4674 | 2 | 2 | 56 | 24 | 2 | 2 | 140 | 90 |  | 6.9 | 1.37 | 1.61 | 4.9 | 33 | 38 | 18 | 3.2 | 48 | 67 | 5 | 13 |
| 4685 | 2 | 1 | 55 | 22.6 | 1 | 2 | 120 | 70 |  | 5.7 | 0.91 | 1.78 | 2.69 | 8 | 27 | 12 | 3.6 | 13 | 101 | 4 | 14 |
| 4689 | 2 | 2 | 58 | 21.1 | 2 | 2 | 120 | 80 |  | 4.8 | 1.64 | 1.3 | 3.58 | 17 | 33 | 6 | 3.7 | 46 | 57 | 5 | 13 |
| 4695 | 2 | 2 | 54 | 20.8 | 1 | 1 | 110 | 70 |  | 3.4 | 1.66 | 0.9 | 1.3 | 32 | 21 | 12 | 4.4 | 20 | 103 | 6 | 14 |
| 4698 | 2 | 2 | 50 | 23.9 | 2 | 2 | 110 | 70 |  | 7.3 | 0.64 | 1.82 | 5.32 | 36 | 18 | 15 | 4 | 30 | 60 | 5 | 12 |
| 4700 | 2 | 2 | 52 | 23.4 | 2 | 2 | 120 | 80 |  | 7.5 | 3.02 | 1.12 | 4.92 | 19 | 41 | 3.9 | 4 | 45 | 43 | 6 | 13 |
| 4702 | 2 | 1 | 54 | 22.7 | 1 | 1 | 90 | 60 |  | 1.8 | 1.61 | 1.16 | 3.46 | 32 | 24 | 13 | 2.6 | 48 | 75 | 4 | 13 |
| 4713 | 2 | 2 | 58 | 26 | 2 | 2 | 140 | 90 |  | 3.3 | 0.78 | 1.82 | 1.44 | 31 | 20 | 11 | 1.7 | 8 | 38 | 5 | 12 |
| 4715 | 2 | 1 | 50 | 20 | 2 | 2 | 120 | 80 |  | 3.1 | 2.77 | 1.39 | 2.59 | 15 | 32 | 14 | 1.1 | 47 | 117 | 6 | 14 |
| 4716 | 2 | 1 | 57 | 21.6 | 1 | 2 | 100 | 60 |  | 2.7 | 1.29 | 1.54 | 2.86 | 13 | 30 | 13 | 3 | 45 | 57 | 4 | 12 |
| 4723 | 2 | 2 | 69 | 20.7 | 1 | 1 | 120 | 80 |  | 5.9 | 1.25 | 1.69 | 2.26 | 31 | 37 | 6.2 | 2.2 | 43 | 53 | 6 | 12 |
| 4733 | 2 | 1 | 60 | 25 | 2 | 2 | 115 | 70 |  | 5.4 | 0.96 | 1.43 | 1.98 | 8 | 20 | 6.4 | 1 | 29 | 87 | 6 | 14 |
| 4734 | 2 | 2 | 49 | 24 | 2 | 2 | 110 | 80 |  | 1.2 | 1.18 | 1.11 | 1.36 | 24 | 19 | 15 | 1.8 | 37 | 60 | 4 | 13 |
| 4749 | 2 | 1 | 59 | 25 | 2 | 2 | 140 | 95 |  | 2.8 | 0.73 | 1.61 | 3.54 | 24 | 8 | 8 | 2.3 | 19 | 62 | 5 | 14 |
| 4750 | 2 | 1 | 22 | 29 | 1 | 2 | 120 | 80 |  | 4 | 0.9 | 1.54 | 4.02 | 27 | 27 | 9.5 | 1.1 | 47 | 54 | 5 | 14 |
| 4761 | 2 | 2 | 52 | 24 | 2 | 2 | 120 | 90 |  | 6.1 | 1.25 | 1.64 | 4.13 | 20 | 26 | 3.5 | 3 | 14 | 72 | 4 | 13 |
| 4820 | 2 | 1 | 31 | 25 | 1 | 1 | 110 | 80 |  | 2.6 | 1.54 | 1.5 | 3.36 | 16 | 29 | 8.4 | 2 | 48 | 72 | 5 | 16 |
| 4823 | 2 | 1 | 25 | 26 | 2 | 2 | 110 | 70 |  | 2.1 | 0.88 | 1.56 | 3.24 | 20 | 36 | 5.2 | 1.8 | 27 | 71 | 5 | 15 |
| 4824 | 2 | 1 | 36 | 23.1 | 1 |  | 130 | 80 |  | 2.2 | 1.46 | 1 | 2.02 | 35 | 22 | 6.2 | 1.6 | 20 | 41 | 4 | 13 |
| 4825 | 2 | 1 | 31 | 21.6 | 2 | 2 | 112 | 70 |  | 1.6 | 1.34 | 1.28 | 1.72 | 44 | 27 | 9.8 | 1.3 | 34 | 43 | 5 | 16 |
| 4827 | 2 | 2 | 37 | 24 | 2 | 2 | 110 | 80 |  | 1.9 | 1.19 | 1.44 | 2.35 | 9 | 24 | 6 | 4.2 | 10 | 60 | 6 | 15 |
| 4830 | 2 | 2 | 68 | 20.2 | 1 | 1 | 110 | 80 |  | 1.3 | 2.83 | 1.12 | 3.53 | 38 | 14 | 14 | 4 | 49 | 88 | 5 | 14 |
| 4835 | 2 | 1 | 31 | 20.2 | 2 | 2 | 110 | 70 |  | 3.5 | 0.82 | 1.33 | 4.13 | 18 | 24 | 5.2 | 2.2 | 34 | 46 | 5 | 14 |
| 4838 | 2 | 1 | 32 | 24 | 1 | 1 | 105 | 70 |  | 2.8 | 1.11 | 1.84 | 3.69 | 34 | 22 | 14 | 2.5 | 13 | 76 | 4 | 11 |
| 4843 | 2 | 2 | 23 | 25 | 2 | 2 | 110 | 80 |  | 1.5 | 1.56 | 1.54 | 2.56 | 35 | 27 | 5.6 | 1.1 | 32 | 53 | 5 | 15 |
| 4882 | 2 | 1 | 27 | 20.8 | 1 | 2 | 110 | 70 |  | 1.8 | 1.09 | 1.77 | 2.83 | 39 | 32 | 12 | 1.1 | 46 | 105 | 4 | 13 |
| 4883 | 2 | 1 | 22 | 21 | 1 | 2 | 120 | 70 |  | 3.3 | 1.32 | 1.21 | 3.33 | 27 | 24 | 16 | 2.7 | 20 | 54 | 5 | 12 |
| 4885 | 2 | 1 | 32 | 20.7 | 2 | 2 | 110 | 70 |  | 2.4 | 0.93 | 1.07 | 1.85 | 12 | 19 | 3.9 | 3 | 48 | 88 | 5 | 12 |
| 4886 | 2 | 1 | 32 | 21.9 | 1 | 1 | 90 | 60 |  | 4.2 | 1.62 | 0.97 | 2.99 | 26 | 29 | 6.1 | 2.5 | 9 | 93 | 6 | 11 |
| 4896 | 2 | 1 | 24 | 24 | 1 | 1 | 120 | 82 |  | 3.6 | 1.63 | 1.46 | 1.82 | 25 | 12 | 4.3 | 4.1 | 46 | 123 | 4 | 11 |
| 4905 | 2 | 1 | 32 | 22 | 2 | 2 | 90 | 60 |  | 5.7 | 1.77 | 1.76 | 2.91 | 12 | 36 | 9.6 | 3.6 | 30 | 92 | 4 | 14 |
| 4910 | 2 | 1 | 25 | 25 | 2 | 2 | 100 | 80 |  | 3.1 | 0.77 | 1.09 | 2.39 | 27 | 36 | 5 | 1.6 | 41 | 77 | 5 | 11 |
| 4912 | 2 | 2 | 45 | 24 | 2 | 2 | 106 | 70 |  | 5.2 | 1.07 | 1.8 | 3.33 | 31 | 20 | 17 | 1.2 | 35 | 118 | 5 | 16 |
| 4915 | 2 | 2 | 60 | 21.6 | 2 | 2 | 120 | 80 |  | 1.2 | 1.31 | 1.08 | 4.09 | 36 | 35 | 4.8 | 3.3 | 12 | 97 | 5 | 14 |
| 4918 | 2 | 1 | 29 | 24 | 2 | 2 | 120 | 70 |  | 5.5 | 2.42 | 1.51 | 1.95 | 30 | 30 | 17 | 2 | 38 | 76 | 6 | 11 |
| 4930 | 2 | 1 | 32 | 21.2 | 2 | 2 | 120 | 84 |  | 2 | 0.89 | 1.64 | 2.11 | 20 | 20 | 9.7 | 2.5 | 49 | 89 | 5 | 13 |
| 4933 | 2 | 1 | 31 | 21.5 | 2 | 2 | 140 | 90 |  | 4 | 0.85 | 1.76 | 2.87 | 44 | 43 | 4 | 3.9 | 8 | 82 | 6 | 15 |
| 4934 | 2 | 2 | 56 | 22.2 | 2 | 2 | 140 | 90 |  | 2.1 | 1.55 | 1.56 | 3.56 | 26 | 32 | 4.4 | 3.6 | 21 | 92 | 6 | 13 |
| 4936 | 2 | 1 | 25 | 24 | 2 | 2 | 110 | 70 |  | 3.3 | 1.9 | 1.56 | 3.66 | 28 | 24 | 14 | 3.7 | 20 | 69 | 5 | 14 |
| 4937 | 2 | 2 | 26 | 26 | 2 | 2 | 110 | 70 |  | 3.7 | 1.62 | 1.23 | 2.67 | 28 | 19 | 7 | 1.5 | 11 | 84 | 6 | 12 |
| 4955 | 2 | 1 | 30 | 22.8 | 1 | 2 | 110 | 80 |  | 2.2 | 1.2 | 1.84 | 3.77 | 37 | 15 | 4.9 | 1.3 | 35 | 92 | 4 | 14 |
| 4962 | 2 | 1 | 33 | 21.8 | 2 | 2 | 110 | 70 |  | 3.5 | 1.5 | 1.45 | 3.35 | 25 | 12 | 11 | 3.3 | 30 | 91 | 6 | 15 |
| 4969 | 2 | 1 | 30 | 21 | 2 | 2 | 110 | 80 |  | 4.3 | 1.51 | 1.23 | 1.81 | 8 | 14 | 6.5 | 1.9 | 18 | 51 | 6 | 12 |
| 4974 | 2 | 1 | 34 | 18.8 | 2 | 2 | 120 | 80 |  | 3.1 | 0.84 | 1.26 | 1.94 | 19 | 33 | 8 | 2.9 | 25 | 55 | 4 | 13 |
| 5059 | 2 | 1 | 28 | 21.3 | 1 | 1 | 100 | 70 |  | 2.4 | 1.35 | 1.17 | 1.44 | 10 | 37 | 17 | 1.2 | 36 | 115 | 5 | 13 |
| 5069 | 2 | 1 | 26 | 21.6 | 2 | 2 | 106 | 70 |  | 1.3 | 1.37 | 1.4 | 3.84 | 17 | 39 | 4.5 | 1.4 | 10 | 110 | 4 | 15 |
| 5070 | 2 | 1 | 29 | 19.4 | 2 | 2 | 120 | 80 |  | 5.4 | 1.04 | 1.27 | 2.04 | 41 | 33 | 12 | 3.3 | 50 | 70 | 5 | 14 |
| 5078 | 2 | 2 | 50 | 24 | 1 | 2 | 110 | 70 |  | 5.5 | 1.46 | 1.09 | 4.02 | 40 | 12 | 10 | 4.2 | 34 | 106 | 5 | 13 |
| 5083 | 2 | 1 | 24 | 19.5 | 2 | 2 | 90 | 60 |  | 5.7 | 1.38 | 1.2 | 2.55 | 20 | 19 | 5.8 | 3.2 | 24 | 56 | 4 | 12 |
| 5085 | 2 | 1 | 29 | 21.1 | 2 | 2 | 100 | 70 |  | 1 | 1.19 | 1.42 | 1.69 | 8 | 35 | 14 | 1.3 | 48 | 55 | 5 | 15 |
| 5086 | 2 | 1 | 33 | 22 | 1 | 2 | 110 | 78 |  | 4.6 | 0.76 | 1.47 | 1.5 | 23 | 13 | 12 | 3 | 41 | 75 | 5 | 16 |
| 5093 | 2 | 1 | 32 | 21.4 | 2 | 2 | 100 | 70 |  | 2.4 | 0.61 | 1.81 | 3.8 | 16 | 32 | 7.3 | 3.9 | 17 | 95 | 4 | 13 |
| 5100 | 2 | 2 | 32 | 24 | 2 | 2 | 100 | 70 |  | 2.5 | 1.29 | 1.06 | 4.04 | 19 | 13 | 9.4 | 1.8 | 41 | 86 | 5 | 12 |
| 5101 | 2 | 2 | 34 | 26 | 2 | 2 | 110 | 80 |  | 5.5 | 1.33 | 1.67 | 3.85 | 20 | 29 | 9.1 | 1 | 11 | 44 | 6 | 12 |
| 5102 | 2 | 1 | 35 | 18 | 2 | 2 | 120 | 84 |  | 2.9 | 0.7 | 1.6 | 1.7 | 14 | 22 | 10 | 3.2 | 44 | 65 | 5 | 15 |
| 5104 | 2 | 1 | 45 | 24 | 2 | 2 | 130 | 80 |  | 4 | 1.41 | 1 | 1.47 | 33 | 13 | 17 | 2.9 | 53 | 46 | 6 | 14 |
| 5105 | 2 | 1 | 27 | 20.3 | 2 | 2 | 110 | 70 |  | 3.9 | 1.23 | 1.59 | 1.77 | 32 | 13 | 11 | 4.3 | 31 | 62 | 4 | 15 |
| 5106 | 2 | 1 | 31 | 23.1 | 1 | 2 | 110 | 80 |  | 5.6 | 1.4 | 1.13 | 1.36 | 16 | 16 | 16 | 3.3 | 14 | 125 | 5 | 14 |
| 5113 | 2 | 1 | 33 | 27 | 2 | 2 | 110 | 70 |  | 1.9 | 1.64 | 1.15 | 2.18 | 14 | 15 | 11 | 1 | 8 | 103 | 5 | 13 |
| 5124 | 2 | 2 | 66 | 20.1 | 1 | 2 | 130 | 80 |  | 6.7 | 2.85 | 1.58 | 2.11 | 24 | 36 | 4.2 | 2.7 | 20 | 97 | 6 | 16 |
| 5125 | 2 | 2 | 54 | 19.7 | 2 | 2 | 100 | 70 |  | 4.8 | 0.67 | 1.6 | 1.86 | 17 | 27 | 6.7 | 1.2 | 16 | 120 | 6 | 15 |
| 5130 | 2 | 2 | 64 | 25 | 2 | 2 | 120 | 90 |  | 5.5 | 0.84 | 1.24 | 1.59 | 35 | 22 | 9.6 | 2.5 | 22 | 118 | 6 | 16 |
| 5131 | 2 | 2 | 63 | 24 | 2 | 2 | 140 | 94 |  | 5.3 | 1.6 | 1.18 | 3.36 | 37 | 28 | 9.7 | 1.4 | 43 | 44 | 6 | 11 |
| 5182 | 2 | 1 | 52 | 25 | 2 | 2 | 140 | 90 |  | 1.9 | 1.29 | 1.68 | 2.96 | 26 | 20 | 15 | 1.6 | 13 | 95 | 6 | 12 |
| 5183 | 2 | 1 | 45 | 24 | 2 | 2 | 100 | 70 |  | 3.9 | 0.84 | 1.6 | 2.68 | 20 | 19 | 17 | 3.5 | 36 | 121 | 4 | 15 |
| 5227 | 2 | 1 | 40 | 21.6 | 1 | 2 | 110 | 84 |  | 5 | 1.14 | 1.11 | 2.27 | 27 | 12 | 11 | 2.3 | 39 | 53 | 5 | 11 |
| 5231 | 2 | 1 | 40 | 22.5 | 2 | 2 | 110 | 70 |  | 3.7 | 1.38 | 1.08 | 3.8 | 17 | 38 | 7.9 | 3 | 26 | 103 | 6 | 13 |
| 5233 | 2 | 1 | 29 | 22.8 | 2 | 2 | 90 | 60 |  | 2.4 | 0.89 | 1.37 | 4.08 | 13 | 32 | 4.6 | 2.2 | 40 | 121 | 6 | 14 |
| 5239 | 2 | 1 | 27 | 24 | 2 | 2 | 110 | 70 |  | 1.2 | 1.4 | 1.16 | 1.86 | 38 | 19 | 16 | 6.1 | 12 | 85 | 4 | 14 |
| 5245 | 2 | 2 | 55 | 24 | 2 | 2 | 120 | 72 |  | 3.8 | 1.24 | 1.82 | 2.33 | 28 | 10 | 15 | 1.3 | 26 | 89 | 6 | 13 |
| 5248 | 2 | 2 | 50 | 20.8 | 1 | 2 | 110 | 80 |  | 5.6 | 0.75 | 1.53 | 3.46 | 19 | 21 | 7 | 2.2 | ## | 119 | 5 | 15 |
| 5305 | 2 | 1 | 68 | 22.9 | 1 | 1 | 130 | 80 |  | 3.9 | 1.6 | 1.8 | 2.24 | 38 | 16 | 16 | 4.1 | 19 | 125 | 4 | 16 |
| 5311 | 2 | 2 | 56 | 21.4 | 2 | 2 | 120 | 80 |  | 6.9 | 1.18 | 1.37 | 3.45 | 8 | 30 | 5.7 | 3 | 16 | 96 | 4 | 11 |
| 5321 | 2 | 2 | 50 | 24 | 2 | 2 | 105 | 70 |  | 5.1 | 1.08 | 1.05 | 2.3 | 34 | 23 | 8.1 | 3.7 | 47 | 122 | 6 | 14 |
| 5327 | 2 | 2 | 51 | 18.6 | 1 | 2 | 112 | 70 |  | 6 | 1.57 | 1.21 | 4.23 | 15 | 40 | 8.9 | 3.7 | 25 | 45 | 5 | 16 |
| 5340 | 2 | 2 | 34 | 24 | 2 | 2 | 100 | 70 |  | 6.1 | 1.56 | 1.55 | 3.26 | 34 | 32 | 8.4 | 1.9 | 25 | 72 | 6 | 15 |
| 5345 | 2 | 2 | 56 | 20.6 | 2 | 2 | 120 | 80 |  | 5.3 | 1.06 | 1.54 | 1.59 | 36 | 17 | 17 | 1 | 38 | 111 | 5 | 14 |
| 5347 | 2 | 1 | 52 | 21.3 | 2 | 2 | 130 | 90 |  | 5.9 | 1.94 | 1.6 | 1.75 | 34 | 39 | 21 | 3.6 | 24 | 70 | 4 | 11 |
| 5359 | 2 | 1 | 31 | 24 | 2 | 2 | 100 | 70 |  | 1.1 | 1.57 | 1.19 | 3.3 | 18 | 40 | 22 | 7.8 | 24 | 43 | 6 | 16 |
| 5360 | 2 | 1 | 40 | 24 | 1 | 2 | 110 | 70 |  | 6.2 | 2.6 | 0.89 | 4.49 | 17 | 23 | 13 | 1.7 | 14 | 79 | 6 | 12 |
| 5361 | 2 | 2 | 56 | 24 | 1 | 2 | 100 | 70 |  | 2.5 | 0.79 | 1.12 | 1.66 | 26 | 36 | 14 | 3.3 | 22 | 114 | 4 | 15 |
| 5365 | 2 | 1 | 42 | 22.5 | 1 | 2 | 110 | 70 |  | 1.2 | 0.96 | 1.3 | 1.83 | 39 | 40 | 7.8 | 4.1 | 45 | 40 | 6 | 15 |
| 5366 | 2 | 2 | 53 | 21 | 1 | 2 | 110 | 80 |  | 5.1 | 1.6 | 1.38 | 2.16 | 37 | 29 | 12 | 2.4 | 32 | 52 | 5 | 12 |
| 5369 | 2 | 1 | 36 | 19.4 | 2 | 2 | 116 | 70 |  | 1.2 | 1.02 | 1.44 | 3.9 | 19 | 34 | 6.6 | 2.4 | 37 | 71 | 5 | 11 |
| 5371 | 2 | 1 | 30 | 16 | 1 | 1 | 111 | 70 |  | 4.7 | 1.46 | 1.52 | 1.37 | 41 | 13 | 10 | 3.5 | 28 | 104 | 5 | 12 |
| 5381 | 2 | 2 | 41 | 24 | 1 | 2 | 110 | 70 |  | 5.4 | 1.17 | 1.11 | 2.21 | 14 | 14 | 3.5 | 3.7 | ## | 81 | 5 | 12 |
| 5402 | 2 | 2 | 53 | 20.2 | 2 | 2 | 105 | 70 |  | 2.9 | 0.95 | 1.72 | 1.35 | 34 | 36 | 16 | 1.2 | 11 | 42 | 5 | 12 |
| 5405 | 2 | 2 | 53 | 19.2 | 1 | 2 | 120 | 80 |  | 4.5 | 1.53 | 1.43 | 1.8 | 17 | 27 | 13 | 4.2 | 53 | 66 | 4 | 14 |
| 5418 | 2 | 1 | 39 | 22.7 | 2 | 2 | 110 | 80 |  | 3.4 | 1.43 | 1.17 | 2.41 | 33 | 37 | 14 | 1.1 | 44 | 58 | 6 | 15 |
| 5421 | 2 | 1 | 35 | 23.1 | 2 | 2 | 120 | 80 |  | 5.8 | 0.83 | 1.1 | 2.67 | 19 | 36 | 14 | 2.7 | 11 | 92 | 5 | 14 |
| 5424 | 2 | 1 | 56 | 20.7 | 2 | 2 | 116 | 70 |  | 4.3 | 0.74 | 1.17 | 2.34 | 32 | 41 | 12 | 2.7 | 31 | 56 | 6 | 16 |
| 5431 | 2 | 2 | 53 | 24 | 2 | 2 | 120 | 80 |  | 5.3 | 0.7 | 1.82 | 2.77 | 40 | 12 | 5.6 | 3.1 | 47 | 108 | 6 | 11 |
| 5434 | 2 | 1 | 42 | 23.2 | 2 | 2 | 110 | 70 |  | 5.2 | 1.87 | 1.35 | 2.59 | 14 | 9 | 14 | 1.7 | 26 | 125 | 4 | 14 |
| 5438 | 2 | 2 | 22 | 18.9 | 2 | 2 | 100 | 70 |  | 4.7 | 0.67 | 1.26 | 1.34 | 11 | 12 | 11 | 3.4 | 8 | 42 | 4 | 15 |
| 5440 | 2 | 1 | 29 | 24 | 2 | 2 | 120 | 80 |  | 5.8 | 1.71 | 1.75 | 3.19 | 34 | 26 | 16 | 2.9 | 19 | 57 | 5 | 12 |
| 5444 | 2 | 1 | 26 | 27 | 2 | 2 | 110 | 70 |  | 1.1 | 0.81 | 1.61 | 3.08 | 19 | 39 | 8.5 | 1.4 | 46 | 96 | 5 | 14 |
| 5453 | 2 | 2 | 21 | 21.5 | 1 | 1 | 90 | 60 |  | 3.1 | 1.21 | 1.31 | 2.25 | 37 | 40 | 5 | 4.3 | 12 | 93 | 5 | 12 |
| 5454 | 2 | 2 | 28 | 19.7 | 2 | 2 | 120 | 80 |  | 1 | 0.87 | 1.57 | 2.66 | 22 | 34 | 11 | 1.8 | 21 | 104 | 6 | 15 |
| 5459 | 2 | 2 | 23 | 20.1 | 2 | 2 | 110 | 70 |  | 5.1 | 1.22 | 1.46 | 2.22 | 10 | 28 | 10 | 3.8 | 46 | 126 | 5 | 15 |
| 5470 | 2 | 1 | 30 | 20.2 | 2 | 2 | 110 | 80 |  | 5.4 | 1.46 | 1.4 | 3.07 | 10 | 18 | 6.3 | 4.1 | 26 | 53 | 6 | 11 |
| 5481 | 2 | 1 | 37 | 23.3 | 2 | 2 | 100 | 70 |  | 4.2 | 0.99 | 1.63 | 3.15 | 8 | 36 | 6.8 | 4.3 | 33 | 43 | 6 | 16 |
| 5483 | 2 | 1 | 62 | 24 | 2 | 2 | 110 | 70 |  | 5 | 1.6 | 1 | 2.77 | 31 | 12 | 11 | 3 | 48 | 60 | 5 | 13 |
| 5490 | 2 | 1 | 31 | 21.1 | 2 | 2 | 100 | 70 |  | 5.9 | 1.11 | 1.6 | 4.16 | 37 | 17 | 16 | 1.6 | 39 | 58 | 6 | 14 |
| 5526 | 2 | 1 | 32 | 27 | 2 | 2 | 130 | 80 |  | 5.8 | 0.71 | 1.03 | 4.33 | 12 | 17 | 6.3 | 2.7 | 35 | 104 | 5 | 16 |
| 5534 | 2 | 1 | 52 | 20.5 | 2 | 2 | 120 | 75 |  | 3.5 | 1.51 | 1.6 | 3.06 | 28 | 31 | 10 | 3.1 | 19 | 42 | 6 | 15 |
| 5545 | 2 | 2 | 27 | 20.3 | 2 | 2 | 90 | 60 |  | 2.5 | 1.27 | 1.09 | 3.9 | 32 | 25 | 3.6 | 3.5 | 13 | 89 | 4 | 13 |
| 5553 | 2 | 1 | 64 | 21.1 | 1 | 2 | 120 | 80 |  | 6.7 | 1.28 | 0.97 | 5.16 | 17 | 17 | 6.7 | 1 | 8 | 68 | 5 | 12 |
| 5561 | 2 | 1 | 35 | 21.8 | 2 | 2 | 120 | 70 |  | 4.7 | 0.88 | 1.07 | 1.62 | 17 | 14 | 17 | 3.1 | 46 | 95 | 5 | 13 |
| 5562 | 2 | 1 | 60 | 19.7 | 1 | 2 | 130 | 80 |  | 2.2 | 0.61 | 1.42 | 2.5 | 21 | 13 | 12 | 4 | 10 | 88 | 4 | 13 |
| 5581 | 2 | 2 | 41 | 25 | 2 | 2 | 110 | 80 |  | 5.3 | 1.09 | 1.49 | 1.86 | 11 | 23 | 14 | 3.9 | 25 | 54 | 4 | 15 |
| 5584 | 2 | 1 | 45 | 29 | 2 | 2 | 116 | 70 |  | 5.8 | 0.99 | 1.04 | 4.06 | 32 | 18 | 10 | 2.4 | 16 | 98 | 4 | 13 |
| 5591 | 2 | 1 | 58 | 22.5 | 1 | 1 | 120 | 80 |  | 3.6 | 1 | 1.3 | 2.59 | 37 | 24 | 11 | 3.2 | 23 | 119 | 4 | 12 |
| 5607 | 2 | 2 | 41 | 24 | 2 | 2 | 102 | 70 |  | 3.2 | 0.84 | 1.64 | 1.37 | 7 | 18 | 7.2 | 1.5 | 42 | 83 | 5 | 12 |
| 5633 | 2 | 2 | 56 | 25 | 2 | 2 | 120 | 70 |  | 1.5 | 0.96 | 1.42 | 1.49 | 15 | 17 | 27 | 9.8 | 24 | 99 | 5 | 12 |
| 5636 | 2 | 2 | 27 | 24 | 2 | 2 | 100 | 70 |  | 1.1 | 1.64 | 1.13 | 1.45 | 28 | 32 | 12 | 3.2 | 50 | 76 | 5 | 14 |
| 5646 | 2 | 2 | 50 | 24 | 1 | 1 | 140 | 90 |  | 5.4 | 1.07 | 1.36 | 3.87 | 33 | 41 | 15 | 2.6 | 31 | 63 | 4 | 12 |
| 5654 | 2 | 1 | 50 | 26 | 1 | 2 | 150 | 104 |  | 2.7 | 1.15 | 1.45 | 2.43 | 10 | 22 | 14 | 1.1 | 37 | 77 | 6 | 13 |
| 5655 | 2 | 1 | 21 | 25 | 2 | 2 | 130 | 80 |  | 4 | 1.49 | 0.93 | 1.36 | 12 | 11 | 9.5 | 1.5 | 16 | 48 | 4 | 14 |
| 5656 | 2 | 2 | 48 | 24 | 2 | 2 | 120 | 76 |  | 6.2 | 0.67 | 1.46 | 4.47 | 31 | 34 | 16 | 3.3 | 44 | 61 | 6 | 12 |
| 5659 | 2 | 1 | 22 | 27 | 1 | 2 | 110 | 70 |  | 1.2 | 1.19 | 1.67 | 3.53 | 26 | 11 | 11 | 3.2 | 20 | 81 | 6 | 13 |
| 5661 | 2 | 1 | 30 | 23 | 2 | 2 | 110 | 70 |  | 1.9 | 0.7 | 1.78 | 2.25 | 18 | 11 | 8.6 | 1.6 | 28 | 104 | 5 | 12 |
| 5662 | 2 | 1 | 56 | 24 | 2 | 2 | 130 | 90 |  | 3.4 | 0.61 | 0.9 | 1.95 | 38 | 12 | 11 | 3.5 | 7 | 97 | 4 | 12 |
| 5663 | 2 | 1 | 57 | 20.9 | 1 | 2 | 150 | 100 |  | 5.9 | 1.32 | 1.79 | 4.16 | 13 | 8 | 8.7 | 3.2 | 18 | 115 | 5 | 12 |
| 5666 | 2 | 1 | 22 | 24 | 1 | 2 | 126 | 84 |  | 4.6 | 1 | 0.88 | 2.45 | 23 | 37 | 3.6 | 1.7 | 20 | 114 | 6 | 11 |
| 5674 | 2 | 1 | 41 | 26 | 1 | 2 | 130 | 80 |  | 5.6 | 0.7 | 1.34 | 2.33 | 34 | 9 | 9.8 | 3.7 | 15 | 69 | 5 | 15 |
| 5676 | 2 | 1 | 30 | 27 | 2 | 2 | 110 | 70 |  | 1 | 1.26 | 1.53 | 3.28 | 10 | 27 | 16 | 3.9 | 14 | 57 | 6 | 14 |
| 5679 | 2 | 1 | 36 | 23 | 2 | 2 | 130 | 80 |  | 5.1 | 1.55 | 1.47 | 2.93 | 11 | 18 | 8.9 | 1.4 | 48 | 92 | 6 | 16 |
| 5692 | 2 | 1 | 59 | 24 | 2 | 2 | 126 | 80 |  | 5.3 | 1.74 | 1.66 | 1.56 | 37 | 19 | 10 | 1 | 33 | 101 | 5 | 15 |
| 5699 | 2 | 1 | 58 | 31 | 2 | 2 | 140 | 80 |  | 1.8 | 0.99 | 1.59 | 2.79 | 10 | 47 | 12 | 3.9 | 81 | 97 | 5 | 12 |
| 5703 | 2 | 1 | 51 | 25 | 1 | 2 | 150 | 96 |  | 2.9 | 0.69 | 1.81 | 2.57 | 21 | 22 | 4.7 | 1 | 36 | 76 | 4 | 13 |
| 5708 | 2 | 1 | 56 | 22.2 | 2 | 2 | 150 | 106 |  | 3.1 | 1.42 | 1.77 | 1.74 | 17 | 23 | 14 | 3.4 | 34 | 42 | 6 | 13 |
| 5710 | 2 | 2 | 51 | 24 | 2 | 2 | 120 | 70 |  | 6 | 1.28 | 1.3 | 3.8 | 34 | 9 | 7.5 | 1.3 | 19 | 51 | 4 | 14 |
| 5712 | 2 | 1 | 58 | 22.2 | 2 | 2 | 130 | 80 |  | 1.9 | 2.25 | 1.45 | 1.54 | 9 | 18 | 8.6 | 2.5 | 8 | 74 | 6 | 14 |
| 5717 | 2 | 1 | 56 | 22 | 2 | 2 | 130 | 90 |  | 1.2 | 1.65 | 1.22 | 3.86 | 29 | 23 | 8.2 | 3.2 | 29 | 51 | 6 | 16 |
| 5732 | 2 | 2 | 33 | 24 | 2 | 2 | 110 | 70 |  | 1.7 | 1.33 | 1.01 | 1.75 | 12 | 13 | 12 | 2.2 | 39 | 57 | 6 | 11 |
| 5735 | 2 | 1 | 49 | 27 | 1 | 2 | 120 | 80 |  | 4.1 | 2.19 | 1.52 | 3.73 | 56 | 59 | 12 | 2.3 | 32 | 103 | 4 | 13 |
| 5747 | 2 | 1 | 28 | 25 | 1 | 1 | 110 | 74 |  | 1.6 | 1.27 | 1.72 | 2.01 | 11 | 10 | 11 | 1.6 | 25 | 47 | 5 | 14 |
| 5748 | 2 | 1 | 53 | 24 | 2 | 2 | 126 | 84 |  | 5.3 | 0.63 | 1.22 | 3.78 | 10 | 35 | 15 | 2.6 | 33 | 53 | 5 | 14 |
| 5750 | 2 | 1 | 40 | 25 | 2 | 2 | 116 | 70 |  | 5.4 | 3.6 | 1.43 | 1.93 | 14 | 26 | 14 | 3.8 | 8 | 123 | 5 | 12 |
| 5754 | 2 | 1 | 41 | 24 | 1 | 1 | 120 | 70 |  | 1.6 | 1.39 | 1.78 | 2.01 | 9 | 33 | 13 | 2.2 | 12 | 54 | 4 | 11 |
| 5763 | 2 | 2 | 46 | 28 | 2 | 2 | 130 | 84 |  | 3.8 | 0.96 | 1.71 | 1.86 | 12 | 17 | 15 | 1.1 | 39 | 47 | 5 | 12 |
| 5766 | 2 | 2 | 49 | 25 | 2 | 2 | 105 | 70 |  | 4.8 | 1.27 | 1.61 | 2.24 | 19 | 36 | 5.7 | 3 | 45 | 125 | 6 | 13 |
| 5768 | 2 | 1 | 65 | 21 | 2 | 2 | 140 | 85 |  | 1.7 | 1.79 | 1.59 | 1.84 | 15 | 16 | 7.7 | 1.2 | 44 | 66 | 4 | 12 |
| 5771 | 2 | 1 | 53 | 24 | 2 | 2 | 134 | 94 |  | 6.1 | 1.32 | 1.68 | 4.15 | 13 | 15 | 9.4 | 1.5 | 41 | 70 | 6 | 15 |
| 5782 | 2 | 2 | 48 | 27 |  | 2 | 130 | 85 |  | 5.6 | 1.03 | 1.68 | 3.68 | 13 | 27 | 8.4 | 3.2 | 20 | 78 | 6 | 13 |
| 5789 | 2 | 1 | 52 | 24 | 2 | 2 | 130 | 90 |  | 6 | 1.07 | 1.23 | 4.19 | 18 | 19 | 14 | 3.2 | 83 | 48 | 5 | 11 |
| 6344 | 2 | 1 | 60 | 22.2 | 2 | 2 | 130 | 88 |  | 1.3 | 1.1 | 1.6 | 2.03 | 24 | 27 | 7.9 | 2.5 | 40 | 111 | 6 | 12 |
| 6348 | 2 | 1 | 39 | 20.1 | 2 | 2 | 90 | 60 |  | 5.7 | 0.92 | 1.18 | 1.42 | 29 | 26 | 15 | 3.9 | 24 | 110 | 6 | 14 |
| 6365 | 2 | 1 | 25 | 24 | 2 | 2 | 120 | 70 |  | 2.7 | 1.26 | 1.03 | 2.85 | 40 | 23 | 16 | 2.5 | 41 | 55 | 6 | 15 |
| 6402 | 2 | 1 | 69 | 19.7 | 1 |  | 120 | 80 |  | 5.5 | 1.61 | 1.16 | 3.57 | 17 | 13 | 14 | 3.8 | 34 | 76 | 5 | 15 |
| 6417 | 2 | 1 | 72 | 24 | 1 | 2 | 118 | 80 |  | 3.1 | 1.32 | 1.53 | 2.65 | 73 | 63 | 8.6 | 2.8 | ## | 46 | 6 | 14 |
| 6436 | 2 | 2 | 43 | 26 | 2 | 2 | 90 | 66 |  | 3.9 | 1.62 | 1.18 | 2.72 | 26 | 12 | 6.6 | 3.8 | 8 | 52 | 5 | 14 |
| 6459 | 2 | 2 | 66 | 25 | 2 | 2 | 130 | 70 |  | 5.1 | 1.13 | 1.68 | 3.46 | 7 | 21 | 3.4 | 2 | 41 | 92 | 4 | 12 |
| 6486 | 2 | 2 | 60 | 25 | 2 | 2 | 125 | 70 |  | 5 | 0.94 | 1.47 | 3.73 | 21 | 38 | 8.8 | 1.8 | 28 | 56 | 5 | 11 |
| 6491 | 2 | 1 | 26 | 24 | 2 | 2 | 100 | 70 |  | 4.4 | 1.67 | 1.42 | 1.66 | 28 | 27 | 5.1 | 2.3 | 19 | 114 | 4 | 12 |
| 6509 | 2 | 1 | 37 | 24 | 2 | 2 | 100 | 70 |  | 5.8 | 1.57 | 1.4 | 3.23 | 30 | 11 | 6.9 | 1.5 | 33 | 41 | 6 | 14 |
| 6519 | 2 | 1 | 75 | 24 | 2 | 2 | 140 | 70 |  | 5.5 | 0.66 | 1.21 | 3.18 | 22 | 27 | 7 | 2.6 | 38 | 96 | 7 | 18 |
| 6520 | 2 | 1 | 68 | 18.5 | 2 | 2 | 120 | 80 |  | 4.8 | 1.04 | 0.99 | 2.49 | 21 | 15 | 6.6 | 3.7 | 18 | 89 | 5 | 13 |
| 6523 | 2 | 2 | 37 | 24 | 2 | 2 | 110 | 74 |  | 3.8 | 1 | 1.49 | 3.46 | 33 | 31 | 16 | 3.9 | 30 | 46 | 4 | 15 |
| 7095 | 2 | 1 | 61 | 21 | 2 | 2 | 120 | 80 |  | 3.2 | 0.8 | 1.36 | 1.68 | 36 | 32 | 14 | 1.4 | 40 | 74 | 6 | 16 |
| 7104 | 2 | 2 | 80 | 23.2 | 1 | 2 | 130 | 80 |  | 5.6 | 0.81 | 1.67 | 3.33 | 22 | 10 | 7.1 | 1.9 | 15 | 102 | 5 | 11 |
| 7137 | 2 | 1 | 62 | 22 | 1 | 2 | 173 | 90 |  | 1.7 | 2.05 | 1.54 | 2.84 | 24 | 19 | 15 | 4.2 | 50 | 85 | 4 | 16 |
| 7146 | 2 | 2 | 37 | 24 | 2 | 2 | 110 | 70 |  | 2.4 | 1.07 | 0.9 | 3.29 | 8 | 36 | 17 | 6.1 | 32 | 53 | 6 | 12 |
| 7745 | 2 | 1 | 62 | 20.6 | 2 | 2 | 110 | 80 |  | 2.7 | 1.7 | 1.69 | 3.16 | 29 | 8 | 5.5 | 1.1 | 44 | 55 | 5 | 15 |
| 7750 | 2 | 1 | 25 | 25.1 | 2 | 2 | 120 | 80 |  | 2.8 | 0.66 | 1.7 | 1.93 | 16 | 21 | 22 | 7.6 | 18 | 111 | 5 | 12 |
| 7755 | 2 | 1 | 43 | 20.8 | 1 | 2 | 106 | 70 |  | 5 | 1.37 | 1.49 | 2.14 | 37 | 30 | 3.9 | 4.1 | 35 | 96 | 5 | 12 |
| 7782 | 2 | 1 | 24 | 26.7 | 1 | 1 | 110 | 70 |  | 2.1 | 1.69 | 1.6 | 2.38 | 14 | 14 | 11 | 3.5 | 38 | 118 | 6 | 13 |
| 7789 | 2 | 1 | 52 | 26.1 | 2 | 2 | 105 | 80 |  | 5.5 | 0.83 | 1.5 | 2.31 | 39 | 12 | 13 | 1.5 | 49 | 77 | 5 | 11 |
| 8316 | 2 | 1 | 47 | 22.1 | 2 | 2 | 110 | 70 |  | 3.5 | 1.24 | 1.59 | 3.04 | 34 | 15 | 15 | 3.3 | ## | 58 | 11 | 30 |
| 8336 | 2 | 1 | 42 | 26 | 2 | 2 | 110 | 80 |  | 3.4 | 1.05 | 1.55 | 2.42 | 8 | 31 | 10 | 1.9 | 47 | 47 | 6 | 15 |
| 8337 | 2 | 1 | 48 | 24 | 2 | 2 | 120 | 70 |  | 3.6 | 1.78 | 1.16 | 2.97 | 21 | 40 | 9.8 | 2.5 | 50 | 118 | 5 | 15 |
| 8346 | 2 | 1 | 61 | 23.2 | 2 | 2 | 110 | 76 |  | 4.9 | 0.65 | 1.39 | 3.72 | 12 | 8 | 3.9 | 3.1 | 31 | 121 | 4 | 12 |
| 8350 | 2 | 2 | 47 | 26 | 1 | 2 | 120 | 84 |  | 6 | 1.33 | 1.07 | 4.19 | 18 | 19 | 6.2 | 3.5 | 11 | 116 | 6 | 12 |
| 8352 | 2 | 1 | 47 | 24 | 2 | 2 | 150 | 84 |  | 4.9 | 1.56 | 1.06 | 3.06 | 37 | 30 | 14 | 4.2 | 29 | 97 | 6 | 15 |
| 8359 | 2 | 2 | 41 | 26 | 2 | 2 | 110 | 70 |  | 6.3 | 1.12 | 1.03 | 4.46 | 28 | 18 | 10 | 4.3 | 14 | 101 | 5 | 14 |
| 8366 | 2 | 2 | 75 | 26 | 2 | 2 | 130 | 86 |  | 5.5 | 2.46 | 1.58 | 2.86 | 11 | 51 | 13 | 2.5 | 28 | 124 | 6 | 14 |
| 8367 | 2 | 1 | 75 | 26 | 2 | 2 | 130 | 80 |  | 6.9 | 2.87 | 1.42 | 1.63 | 18 | 15 | 7.9 | 2.1 | 37 | 42 | 5 | 11 |
| 8629 | 2 | 1 | 42 | 25 | 2 | 2 | 120 | 90 |  | 4.1 | 1 | 1.43 | 3.13 | 21 | 21 | 16 | 1.6 | 74 | 69 | 6 | 13 |
| 8639 | 2 | 1 | 28 | 26 | 2 | 2 | 110 | 80 |  | 3.4 | 0.76 | 1.15 | 2.78 | 28 | 26 | 15 | 1.9 | 17 | 54 | 4 | 16 |
| 8642 | 2 | 1 | 55 | 25 | 2 | 2 | 130 | 90 |  | 4.2 | 0.85 | 0.97 | 1.51 | 19 | 17 | 4.4 | 4.2 | 32 | 89 | 6 | 16 |
| 8645 | 2 | 1 | 80 | 20.6 | 2 | 2 | 100 | 60 |  | 3.8 | 1.66 | 1.29 | 2.19 | 12 | 11 | 11 | 4.1 | 34 | 92 | 6 | 12 |
| 8853 | 2 | 1 | 25 | 24 | 2 | 2 | 130 | 80 |  | 3.6 | 0.61 | 0.92 | 1.55 | 38 | 17 | 15 | 2.2 | 43 | 85 | 4 | 14 |
| 8862 | 2 | 1 | 31 | 24 | 1 | 1 | 120 | 70 |  | 2.1 | 0.87 | 1.03 | 1.51 | 40 | 37 | 7.4 | 4.4 | 35 | 67 | 4 | 14 |
| 8887 | 2 | 1 | 60 | 21 | 2 | 2 | 120 | 80 |  | 3.4 | 1.53 | 1.54 | 3.97 | 8 | 26 | 11 | 1.3 | 48 | 78 | 5 | 13 |
